# Ready access to 7,8-dihydroindolo[2,3-*d*][1]benzazepine-6(5*H*)-one scaffold and analogues via early-stage Fischer ring-closure reaction

**DOI:** 10.3762/bjoc.18.15

**Published:** 2022-01-26

**Authors:** Irina Kuznetcova, Felix Bacher, Daniel Vegh, Hsiang-Yu Chuang, Vladimir B Arion

**Affiliations:** 1 Institute of Inorganic Chemistry of the University of Vienna, Währinger Strasse 42, 1090 Vienna, Austria; 2 Institute of Organic Chemistry, Catalysis and Petrochemistry, Department of Organic Chemistry, Slovak Techmical University of Technology in Bratislava, Radlinského 9, SK-81237 Bratislava, Slovak Republic; 3 Institute of Organic Chemistry of the University of Vienna, Währinger Strasse 38, 1090 Vienna, Austria

**Keywords:** anticancer, Fischer indole synthesis, Heck reaction, heterocyclic compounds, indolobenzazepines, latonduines, paullones

## Abstract

Paullone isomers are known as inhibitors of tubulin polymerase and cyclin dependent kinases (Cdks), which are potential targets for cancer chemotherapy. Herein we report an efficient and clean pathway to the fourth isomer, which remained elusive so far, namely 7,8-dihydroindolo[2,3-*d*][1]benzazepin-6(5*H*)-one. Moreover, we demonstrate the generality of our pathway by synthesizing two closely related analogues, one containing a bromo substituent and the other one incorporating an 8-membered instead of a 7-membered ring. The key transformation in this four-step synthesis, with an overall yield of 29%, is the Fischer indole reaction of 2-nitrophenylacetyl acetoacetate with 1-benzyl-1-phenylhydrazine in acetic acid that delivers methyl 2-(1-benzyl-3-(2-nitrophenyl)-1*H*-indol-2-yl)acetate in 55% yield.

## Introduction

**Figure 1:**
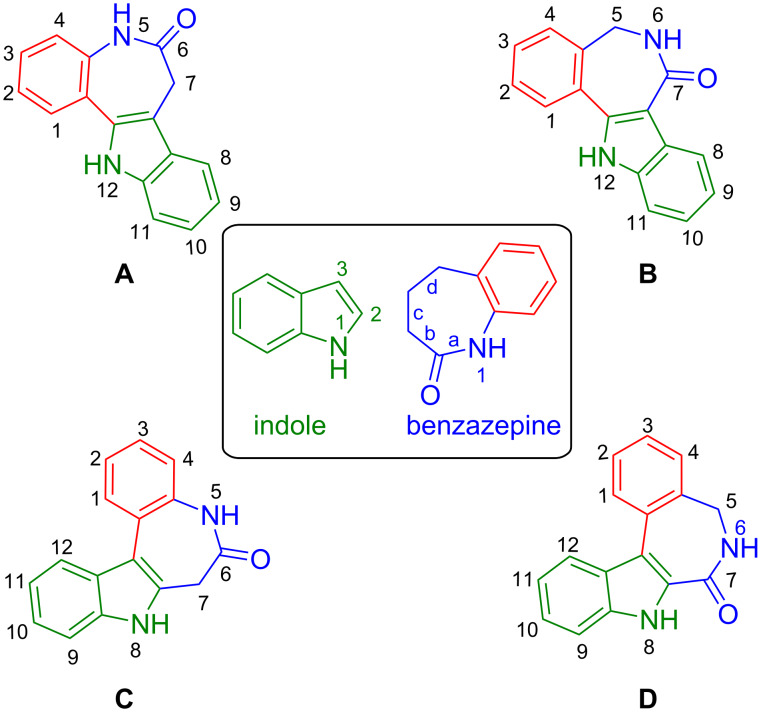
Paullone related indolobenzazepinone isomers. 7,12-Dihydroindolo[3,2-*d*][1]benzazepin-6(5*H*)-one or paullone (**A**), 5,12-dihydroindolo[3,2-*d*][2]benzazepin-7(6*H*)-one (**B**), 7,8-dihydroindolo[2,3-*d*][1]benzazepin-6(5*H*)-one (**C**), and 5,8-dihydroindolo[2,3-*d*][2]benzazepin-7(6*H*)-one (**D**).

Indolobenzazepines are fused heterocyclic scaffolds with versatile medicinal properties, including anti-Alzheimer, anti-inflammatory, anticancer, antidiabetic, and antileishmanial activity [1]. Since the first synthesis of paullones (scaffold **A** in Figure 1) in 1992 [2], and disclosure of their Cdk inhibiting potential, several other analogues were designed and prepared [3], with the hope of developing more efficient anticancer drugs with either improved Cdk targeting or with a different mechanism of action [4–5]. The isomers **B** and **D** (Figure 1) are synthetic derivatives of paullones, in which either the lactam unit is shifted (**B**) or both the lactam unit is shifted and the indole ring is flipped (**D**). Isomer **D** has been shown to be a potent tubulin-polymerase inhibitor, while **B** was only mildly cytotoxic towards cancer cells at a concentration of 1 µM [5–6]. Backbone **C**, despite being very similar to the other three scaffolds, remained a synthetic challenge and practical synthesis is still missing in the literature. Its accessibility may enrich the arsenal of available tools for enzyme inhibitor design by increasing the number of hydrogen bonding donor and acceptor groups at the same side of the backbone, which may result in a tight binding with enzyme active sites and/or improved selectivity [7].

One of the main drawbacks of paullones is their poor aqueous solubility. Therefore, in an attempt to overcome this shortcoming, the paullone backbone **A** was decorated with functional groups and coordinated to metal ions. Ruthenium(II), osmium(II), and copper(II) complexes prepared revealed an enhanced aqueous solubility and bioavailability indeed, along with very high cytotoxicity [8–21]. Moreover, the metal-free ligands and copper(II) complexes derived from backbone **D** revealed cytotoxicity in the nanomolar concentration range and selectivity for cancer cells over normal ones [22]. The synthesis of core structures **A**, **B**, and **D** is well-documented in the literature [2,6]. Herein we describe the first synthesis of 7,8-dihydroindolo[2,3-*d*][1]benzazepin-6(5*H*)-one (**C**) (Figure 1).

## Results and Discussion

**Scheme 1:**
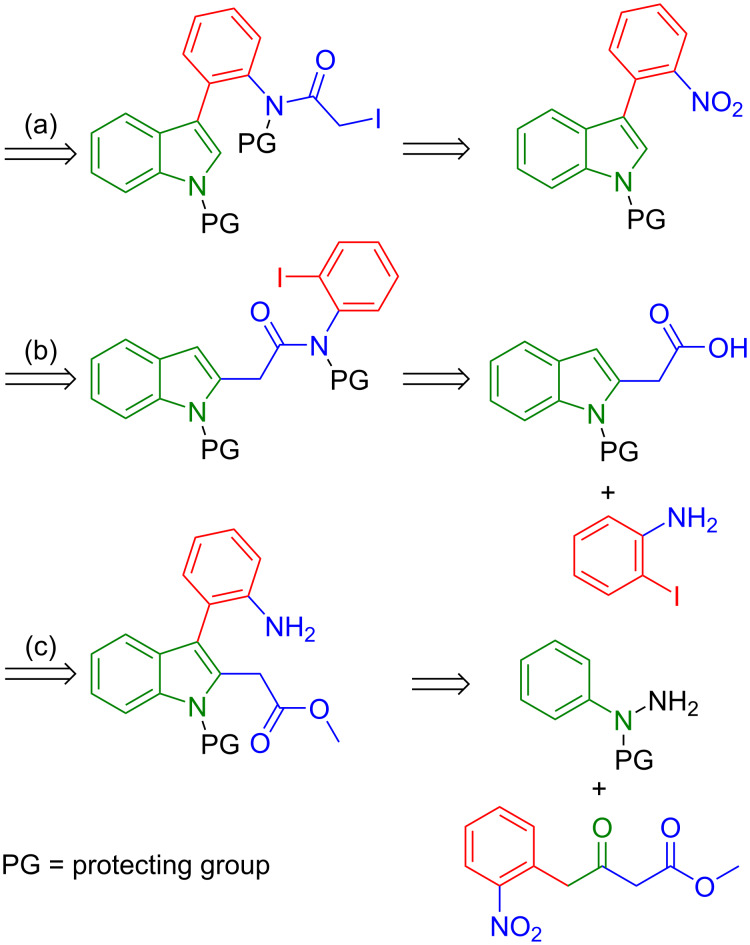
Investigated retrosynthetic pathways to scaffold **C**.

From a retrosynthetic point of view, we followed three main pathways, in order to accomplish the synthesis of scaffold **C**. The first retrosynthetic route (a) started with an alkyl halide precursor, which was expected to afford scaffold **C** after ring-closure reaction at position 2 of the indole ring [23]. The *N*-protected 3-(2-nitrophenyl)indole was considered an important intermediate in this synthetic route (Scheme 1). The second retrosynthetic pathway (b) involved cyclization at position 3 of the indole ring, with a halo-aryl precursor [24]. In this case *N*-protected indole-2-acetic acid was regarded as the key intermediate.

The third pathway (c) was centered around a ring-closure reaction via lactam-bond formation from a precursor that contains a carboxylic ester in position 2 and an *o*-aniline moiety in position 3 of the indole ring by Fischer indole synthesis from methyl 4-(2-nitrophenyl)-3-oxobutanoate (Scheme 1).

**Scheme 2:**
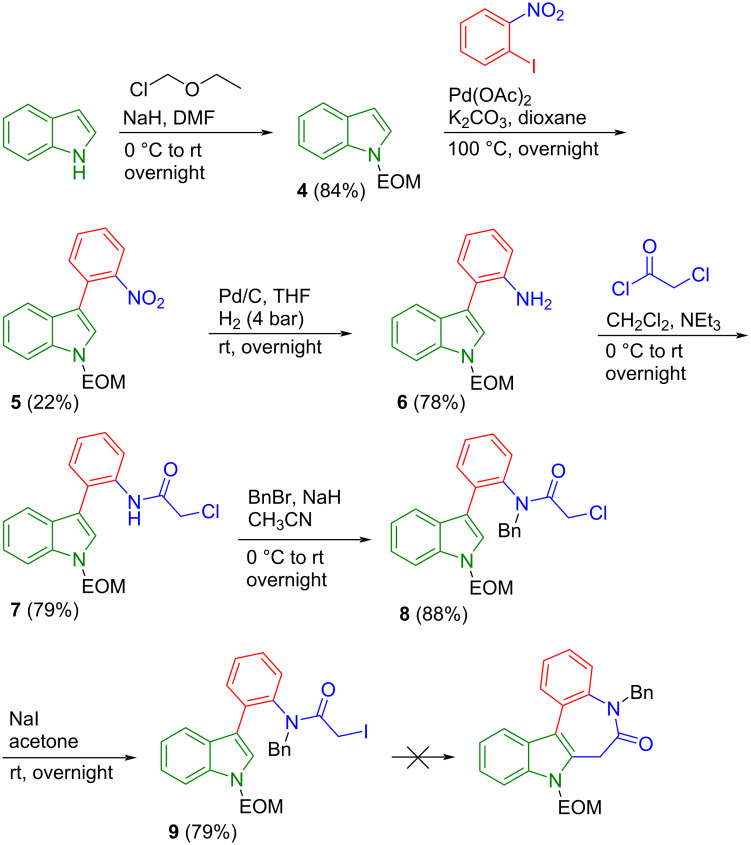
Attempted synthesis of scaffold **C** by route (a).

To construct scaffold **C** by route (a) we first tried to synthesize 3-(2-nitrophenyl)indole by Heck reaction of indole and *o*-iodo-nitrobenzene [25]. However, in this case we obtained an isomeric mixture of 3- and 2-(2-nitrophenyl)indole in a 3:1 molar ratio, quite difficult to separate by column chromatography. Protection of the indole nitrogen by an ethoxymethyl group (Scheme 2) and the use of protected indole **4** in the subsequent Heck reaction resulted in a significantly lower conversion of 22% vs 64% for the unprotected indole. However, the protection of the indole nitrogen atom led exclusively to the desired isomer **5** with the nitrophenyl group in position 3, most likely due to the steric effect. Then, the nitro group in **5** was reduced by hydrogenation under 4 bar using Pd/C as catalyst to yield the desired amine **6** in 78% yield. Subsequently, the desired chloroacetyl derivative **7** was generated in 79% yield by treatment of amine **6** with chloroacetyl chloride. Next, the aminoacetyl group in **7** was benzyl-protected to give **8** in 88% yield. Halogen exchange reaction using excess sodium iodide in acetone gave the desired iodo-alkyl derivative **9** in 79% yield. Lastly, our attempt to accomplish organotin-mediated cyclization [23] of **9** led to a complex mixture of compounds, in which we have not identified the desired product. Therefore, we did not further pursue this route. Attempts to perform cyclization via intramolecular alkyl halide Heck reaction [26] also failed. This is most likely a result of the relatively high lability of the CH_2_ protons in the alkyl halide moiety of the starting molecule, which might lead to enolization instead of oxidative addition of palladium under strongly basic conditions needed in this type of reaction. Thus, we came to the conclusion that pathway (a) is not suitable for the synthesis of scaffold **C**. Nevertheless, a detailed description of the synthesis and spectral data for the intermediate species are given in Supporting Information File 1.

**Scheme 3:**
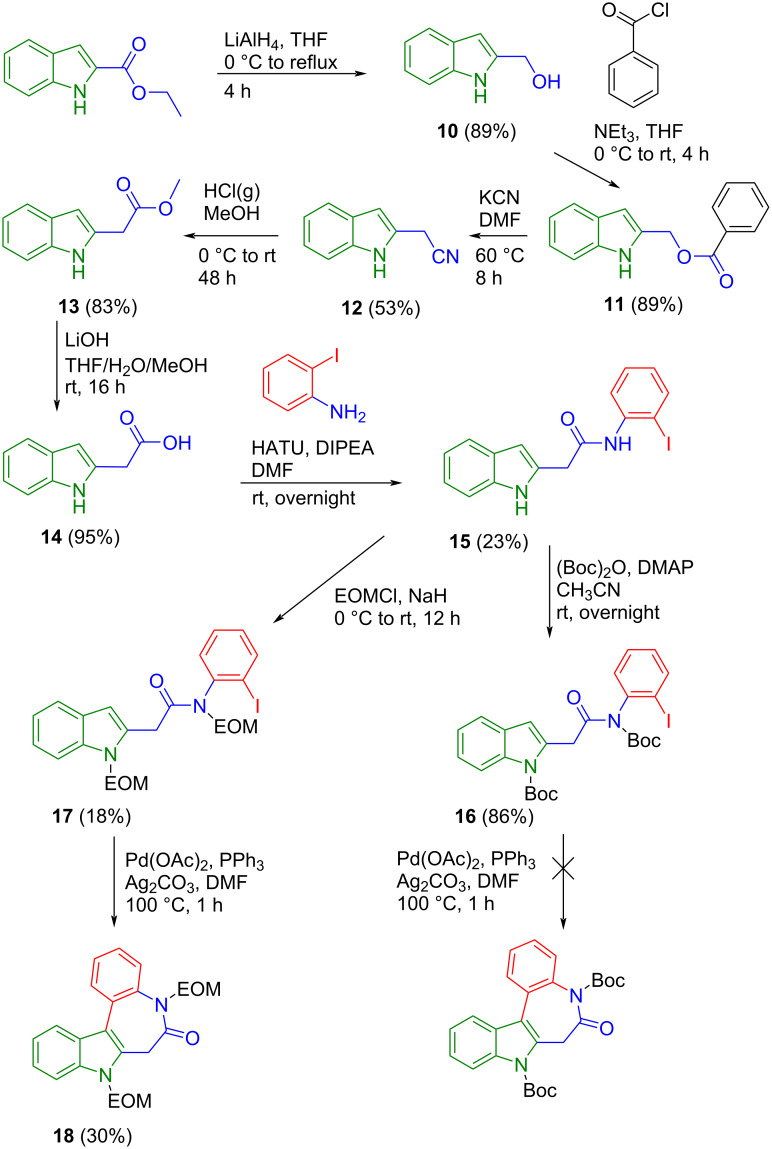
Attempted synthesis of **C** by route (b).

Pathway (b) started with the synthesis of indole-2-acetic acid, which in turn was prepared in five steps as shown in Scheme 3. First, reduction of commercially available indole-2-carboxylate with lithium aluminum hydride in dry tetrahydrofuran gave (1*H*-indole-2-yl)methanol (**10**) in 89% yield. The obtained alcohol was exposed to benzoyl chloride and triethylamine to furnish benzoate **11**, which was further reacted with potassium cyanide to yield nitrile **12** [27]. Saponification of the nitrile was performed by exploiting the Pinner reaction with saturated HCl gas in methanol. This led to methyl indol-2-ylacetate (**13**) in 83% yield [28]. The hydrolysis of ester **13** was performed in the presence of lithium hydroxide monohydrate [29] to give the desired indole-2-acetic acid (**14**) in 95% yield. The peptide coupling reaction [30] of indole-2-acetic acid (**14**) and 2-iodoaniline afforded **15** in 23% yield (Scheme 3). Subsequent protection of both the indole and the amide nitrogen with *tert*-butyloxycarbonyl groups in one step produced **16** in 86% yield. However, attempts to perform the ring-closure reaction of the Boc-protected amide **16** by an intramolecular Heck reaction failed and led to degradation of the starting material. Most likely, the electron-withdrawing *tert*-butyloxycarbonyl protecting group hampered this transformation [24]. Therefore, the same synthetic way was repeated with ethoxymethyl ether as the protecting group to give **17**.

The ESI mass spectrum provided evidence that cyclization occurred with formation of **18**. However, protection with chloromethyl ethyl ether was achieved only in 18% yield. This is most likely due to the strongly basic conditions (NaH) needed for the attachment of the ethoxymethyl protecting group, which might lead to deprotonation at the CH_2_ group (C7) followed by the formation of undesired side products. Being disappointed by the inefficiency of this route with at least two low-yielding transformations we did not further pursue this pathway (Scheme 3).

**Scheme 4:**
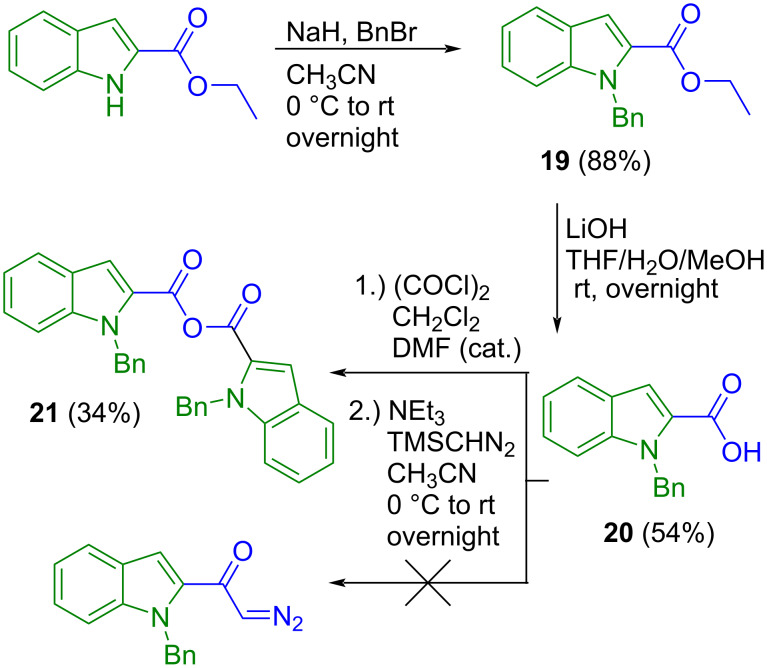
Attempted synthesis of *N*-benzylated indole-2-acetic acid.

**Scheme 5:**
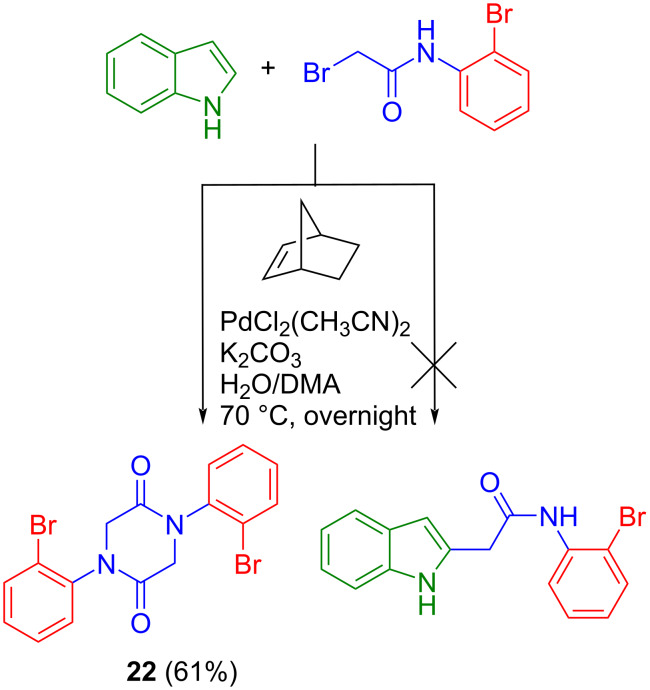
Attempt to obtain open-chain precursor *N*-(2-bromophenyl)-2-(1*H*-indol-2-yl)acetamide.

As an alternative approach to obtain indole-2-acetic acid with a protecting group at the indole nitrogen the application of conditions documented for Arndt–Eistert synthesis [31] was prompted from benzyl-protected indole-2-carboxylic acid **20** (Scheme 4) [32]. While the acid chloride of unsubstituted indole-2-carboxylic acid was reported to be highly unstable [33], we did not experience that issue with the benzyl-protected compound. Quantitative acid chloride formation using oxalyl chloride in dry dichloromethane was observed by TLC. However, the acid chloride reacted very sluggishly with (CH_3_)_3_SiCHN_2_, which is a safe alternative to the highly explosive diazomethane [34]. Another attempt to synthesize the open-chain precursor from indole and 2-bromo-*N*-(2-bromophenyl)acetamide by regioselective palladium-catalyzed norbornene-mediated C–H activation [35] failed and gave 1,4-bis(2-bromophenyl)piperazine-2,5-dione (**22**, Scheme 5) as the sole product in 61% yield, which to our knowledge is not documented in the literature yet.

**Scheme 6:**
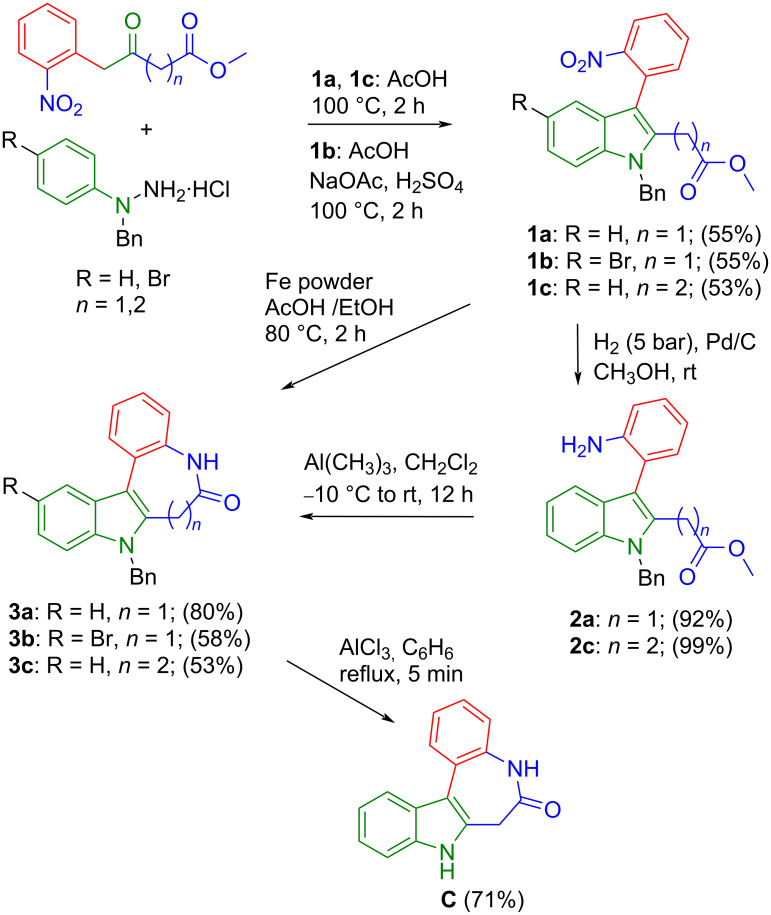
Synthesis of scaffold **C** and analogues by route (c).

**Figure 2:**
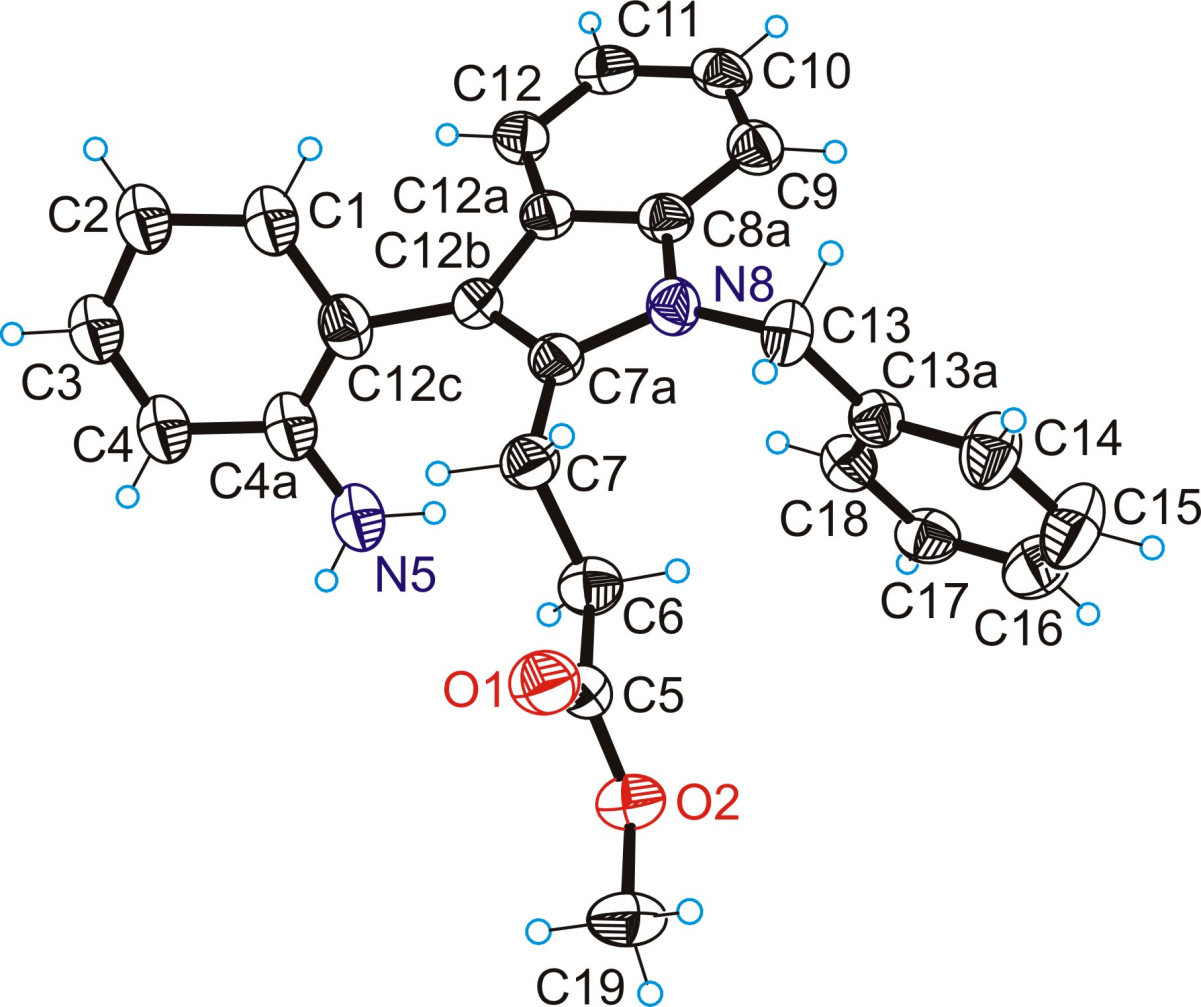
ORTEP view of **1a** with thermal ellipsoids drawn at the 50% probability level.

**Figure 3:**
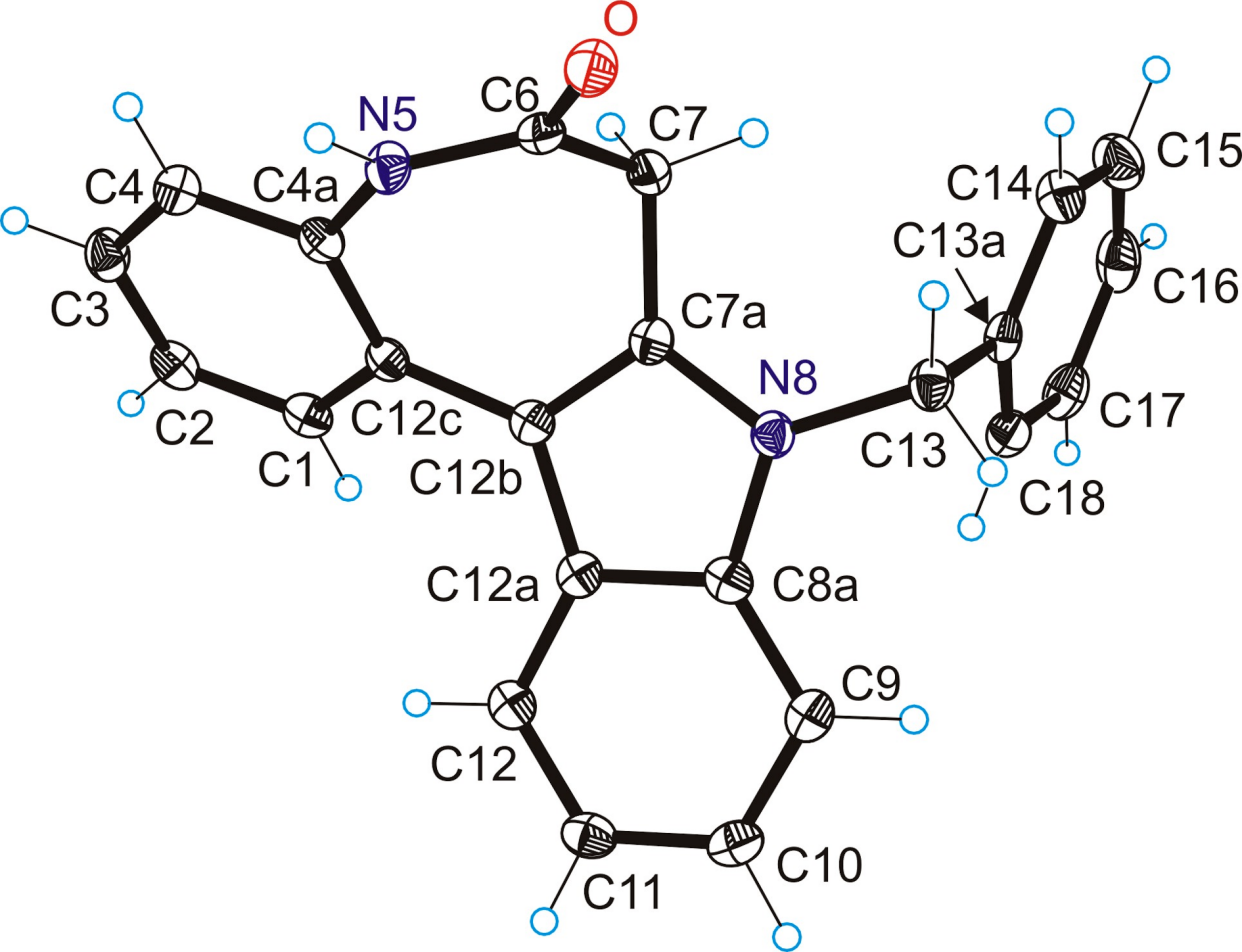
ORTEP view of **3a** with thermal ellipsoids drawn at the 50% probability level.

Synthetic pathway (c) started from methyl 4-(2-nitrophenyl)-3-oxobutanoate prepared in two steps by following known protocols [36]. Fischer indole reaction of this precursor with 1-benzyl-1-phenylhydrazine in acetic acid gave **1a** in 55% yield (Scheme 6). The protecting group on the hydrazine moiety is necessary in this case, otherwise amide bond formation would occur after the initial Schiff base reaction to give undesired 5-(2-nitrobenzyl)-2-phenyl-1,2-dihydro-3*H*-pyrazol-3-one [37]. Note that hypothetically two Fischer indole products could arise from this reaction, namely **1a** and methyl 1-benzyl-2-(2-nitrobenzyl)-1*H*-indole-3-carboxylate, where the ester and nitroaryl moieties are interchanged. However, to our delight only the formation of the desired species **1a** occurred, as evidenced by two-dimensional NMR measurements (for atom labeling scheme used for assignment of resonances see Figure S1 in Supporting Information File 1) and confirmed by SC-XRD (Figure 2), being in agreement with previous observations [38]. Reduction of the nitro group by palladium-catalyzed hydrogenation in dry methanol gave **2a** in 92% yield. Interestingly compound **2a** undergoes ring-closure reaction spontaneously at room temperature to give trace amounts of **3a** (59 mg, 2%) after column chromatography, when a 3.1 g batch of **1a** was reduced. It is likely that **2a** can be cyclized by base catalysis, or by using common peptide coupling reagents (e.g., EDCI, HATU) upon saponification of the ester group. However, we opted for a trimethylaluminum-mediated amidation reaction [4,39] to give rise to 8-benzyl-7-hydroindolo[2,3-*d*][1]benzazepin-6(5*H*)-one (**3a**). This procedure delivered analytically pure **3a** in 80% yield as also confirmed by SC-XRD (Figure 3). The final step to structure **C** required removal of the benzyl group. Debenzylation of amines is commonly performed by palladium-catalyzed hydrogenation [40]. However, this method is not viable for *N*-benzylated indoles, where reduction in liquid ammonia with sodium is usually used as a standard protocol instead, even though successful reports are rare [41]. Other methods involve strongly basic and oxidative conditions [42], which are not tolerated by the CH_2_ group in the lactam ring of **3a**. Gratifyingly, we obtained scaffold **C** in 71% yield by refluxing **3a** with aluminum chloride in benzene (Scheme 6) [43]. For comparison, debenzylation of paullone with sodium in liquid ammonia gave **A** in 40% yield [23].

In addition, the generality of synthetic pathway (c) (Scheme 1) was confirmed by the successful preparation of two closely related analogues, namely **3b**, bearing a bromo substituent at position 11 of the backbone **C** (Scheme 6), and **3c**, with an 8-membered azocinone ring instead of the 7-membered one. The synthesis of **3b** started with a Fischer indole synthesis from methyl 4-(2-nitrophenyl)-3-oxobutanoate [36] and 1-benzyl-1-(4-bromophenyl)hydrazine in the presence of sodium acetate and sulfuric acid in glacial acetic acid affording **1b** in 55% yield. It is of note that the treatment of **1b** with hydrogen in the presence of Pd/C as catalyst led not only to reduction of the nitro group but also to cyclization and the reductive elimination of bromine to afford **3a**. The synthesis of **3b** could be realized in 58% yield by using iron powder under acidic conditions. Reaction of methyl 5-(2-nitrophenyl)-4-oxopentanoate [44] and 1-benzyl-1-phenylhydrazine [36] hydrochloride in glacial acetic acid at 100 °C gave **1c** in 53% yield. Compound **2c** was obtained by palladium-catalyzed reduction of **1c** with hydrogen in an excellent yield and its structure was confirmed by SC-XRD (Figure S4 in Supporting Information File 1). The following ring-closure reaction of **2c** with trimethylaluminum delivered **3c** in 53% yield (Scheme 6).

**Scheme 7:**
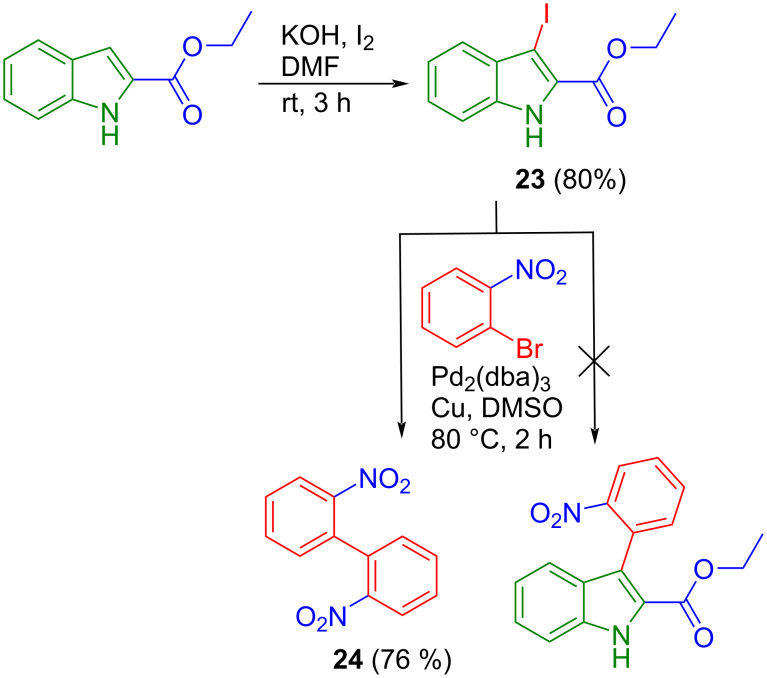
Attempted Ullmann cross-coupling of **23** with *o*-bromo-nitrobenzene.

In an alternative approach we tried to synthesize non-benzylated species **1a** from methyl indol-2-ylacetate. Iodination at position 3 of the indole backbone in the presence of *N*-iodosuccinimide [45] followed by Ullmann cross-coupling with *o*-bromo-nitrobenzene [46] was expected to give non-benzylated **1a**. However, iodination of methyl indol-2-ylacetate led to polymerization reactions involving the CH_2_ protons. In order to investigate the general viability of this synthetic way, we performed iodination at position 3 of ethyl indole-2-carboxylate, which does not contain labile CH_2_ protons. This afforded the iodinated compound **23** in 80% yield. However, Ullmann cross-coupling with *o*-bromo-nitrobenzene gave only trace amounts of the desired product, which we could not separate by chromatographic methods from the major homo-coupling species **24** of this reaction (Scheme 7).

## Conclusion

In summary, a concise, simple, clean and efficient synthesis of paullone isomer **C** was achieved in 4 steps with a crucial transformation, the Fischer indole ring-closure reaction, at the early stage of the synthesis (Scheme 6). The synthetic utility of the elaborated route was validated by gram-scale synthesis with only one step requiring chromatographic purification and inexpensive starting materials. In addition, two failed routes (a) and (b) are described as well, providing useful information for organic chemists and enlarging the scope of this work (Scheme 1). We believe that accessibility to isomer **C** and a number of closely related derivatives with modified electronic and steric properties, e.g., bromo-substituted species, backbone with 8-membered azocinone ring, will trigger the design and synthesis of new exciting anticancer drug candidates, including metal-based, by targeting particular enzymes. Preliminary results indicate that scaffold **C** shows good affinity to proto-oncogene tyrosine-protein kinase Src, a well-known anticancer target [47], with IC_50_ = 0.26 µM and is a suitable candidate for further structural optimization.

## Supporting Information

CCDC 2072772−2072777 and 2103781 contain the supplementary crystallographic data for this paper. These data can be obtained free of charge via http://www.ccdc.cam.ac.uk/data_request/cif, or by emailing data_request@ccdc.cam.ac.uk, or by contacting The Cambridge Crystallographic Data Centre, 12 Union Road, Cambridge CB2 1EZ, UK; fax: +44 1223 336033.

File 1 Experimental procedures, characterization data and ^1^H and ^13^C NMR spectra of compounds, NMR numbering schemes and X-ray crystallography data.
