# Supplementary material for: Ready access to 7,8-dihydroindolo[2,3-d][1]benzazepine-6(5H)-one scaffold and analogues via early-stage Fischer ring-closure reaction
Source: Beilstein J Org Chem. 2022 Jan 26;18:143–51. doi: 10.3762/bjoc.18.15 (PMC8805037; doi:10.3762/bjoc.18.15)
Supplement: File 1 — Experimental procedures, characterization data and 1H and 13C NMR spectra of compounds, NMR numbering schemes and X-ray crystallography data. [file Beilstein_J_Org_Chem-18-143-s001.pdf]

BEILSTEIN JOURNAL OF ORGANIC CHEMISTRY

Supporting Information

for

Ready access to 7,8-dihydroindolo[2,3-*d*][1]benzazepine-6(5*H*)-one scaffold and analogues via early-stage Fischer ring-closure reaction

Irina Kuznetcova, Felix Bacher, Daniel Vegh, Hsiang-Yu Chuang and Vladimir B. Arion

Beilstein J. Org. Chem. **2022,** *18,* 143–151. doi:10.3762/bjoc.18.15

Experimental procedures, characterization data and ^{1}H and ^{13}C NMR spectra of compounds, NMR numbering schemes and X-ray crystallography data

License and Terms: This is a supporting information file under the terms of the Creative Commons Attribution License (https://creativecommons.org/licenses/by/4.0). Please note that the reuse, redistribution and reproduction in particular requires that the author(s) and source are credited and that individual graphics may be subject to special legal provisions.
The license is subject to the *Beilstein Journal of Organic Chemistry* terms and conditions: (https://www.beilstein-journals.org/bjoc/terms)

Table of contents

NMR numbering schemes S2
Synthesis S3
X-ray crystallography data S21
NMR spectra S22

NMR numbering schemes

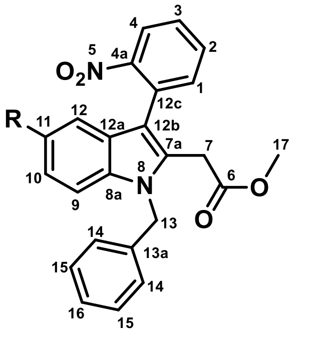

1a: R=H
1b: R=Br

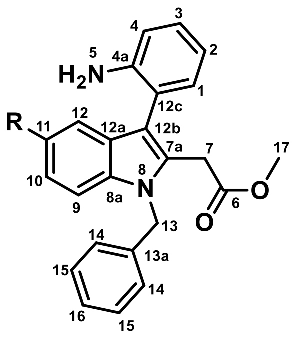

2a: R=H
2b: R=Br

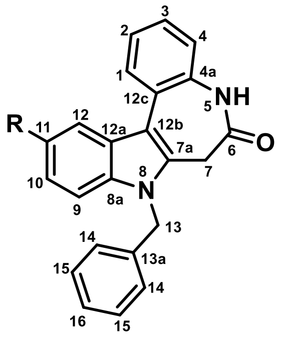

3a: R=H
3b: R=Br

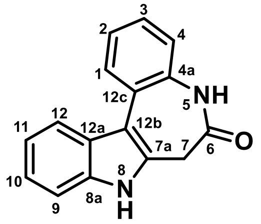

C

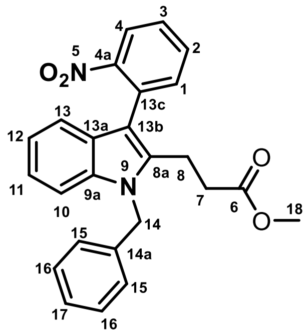

1c

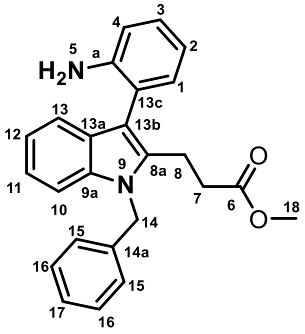

2c

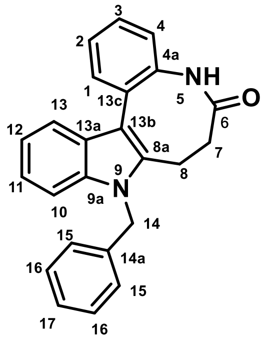

3c

Figure S1: Atom numbering schemes for assignment of resonances in ^{1}H and ^{13}C NMR spectra of **1a, 1b, 1c, 2a, 2c, 3a, 3b, 3c** and **C**.

Synthesis

General Information. NMR spectra were recorded on Bruker AV700, AV600 or AV500 spectrometers; ^{1}H and ^{13}C NMR chemical shifts (δ) are given in ppm relative to TMS, using the residual solvent signals as references and converting the chemical shifts to the TMS scale. ESI-MS spectra were recorded on a Bruker amaZon speed ETD spectrometer (3D-ion trap). High resolution ESI spectra were recorded on a Bruker maXis UHR-TOF spectrometer. Single crystal X-ray diffraction analysis: X-ray diffraction data collections were performed on Bruker X8 APEX-II CCD (**14**), Bruker D8 Venture (**1**, **3**, **21**, **22**, **24**) and STOE (**2c**) diffractometers by using Mo Kα (**1**, **3**, **14**, **21**, **24**) or Cu Kα (**2c**, **22**) radiation. The structures were solved by direct methods and refined by full-matrix least-squares techniques. Non-H atoms were refined with anisotropic displacement parameters. H atoms were inserted in calculated positions and refined with a riding model. The following computer programs and hardware were used: structure solution, *SHELXS-2014* and refinement, *SHELXL-2014*;[1] molecular diagrams, *ORTEP*; computer, Intel CoreDuo.

Methyl 4-(2-nitrophenyl)-3-oxobutanoate was synthesized according to literature protocols [2]. 2-Bromo-*N*-(2-bromophenyl)acetamide was prepared by a known procedure [3]. Chloromethyl ethyl ether, methyl 4-chloro-4-oxobutyrate, 2-fluoronitrobenzene, bis(acetonitrile)palladium(II) dichloride and tris(dibenzylideneacetone)dipalladium(0) were purchased from TCI Europe. Sodium hydride, palladium(II) acetate, palladium on activated charcoal (10% Pd), lithium hydroxide monohydrate, 2-nitrophenylacetic acid, methyl acetoacetate, sodium methylate, aluminum chloride, trimethylaluminum, 2-iodoaniline, *tert*-butanol, iron powder, *N*,*N*-diisopropylethylamine, di-*tert*-butyl dicarbonate, 4-dimethylaminopyridine, bicyclo[2.2.1]hept-2-ene (norbornene), and sodium bisulfite were purchased from Sigma-Aldrich. *o*-Iodo-nitrobenzene, *o*-bromo-nitrobenzene, 1-[bis(dimethylamino)methylene]-1*H*-1,2,3-triazolo[4,5-*b*]pyridinium 3-oxide hexafluorophosphate (HATU), 4-bromophenyl hydrazine, 1-benzyl-1-phenylhydrazine hydrochloride, silver carbonate, and indole-2-carboxylate were from abcr. Potassium carbonate, thionyl chloride, chloroacetyl chloride, benzyl bromide, triethylamine, sodium iodide, trimethylsilyl diazomethane, pyridine, sodium methoxide, 2,2-dimethyl-1,3-dioxan-4,6-dione, sodium thiosulfate, dry

dimethylformamide, dry dioxane, dry acetonitrile, and potassium hydroxide were from Fisher Scientific GmbH. Lithium aluminum hydride, benzoyl chloride, potassium cyanide, and iodine were purchased from Merck. Triphenylphosphine, oxalyl chloride, and copper powder were from Alfa Aesar. Gaseous hydrogen chloride was obtained by dropwise addition of sulfuric acid to sodium chloride. Solvents were dried using standard procedures [4]. Reactions in dry solvents were carried out under argon, if not mentioned otherwise.

Synthesis of compounds C, 3b, and 3c

*Methyl 2-(1-benzyl-3-(2-nitrophenyl)-1H-indol-2-yl)acetate (**1a**).* A suspension of methyl 4-(2-nitrophenyl)-3-oxobutanoate (0.50 g, 2.11 mmol) and 1-benzyl-1-phenylhydrazine hydrochloride (0.48 g, 2.06 mmol) in glacial acetic acid (10 mL) was stirred at 100 °C for 2 h. After cooling to room temperature water (25 mL) was added and the mixture was stored at 4 °C overnight. The supernatant liquid was decanted from a yellow oily residue. The oil was washed with saturated aqueous sodium bicarbonate solution and extracted with ethyl acetate (3 × 5 mL). The combined organic layers were dried over sodium sulfate and concentrated under reduced pressure. Recrystallization from methanol gave **1a** as a bright yellow solid. Yield: 0.45 g, 55%. ^{1}H NMR (600 MHz, DMSO-d_6), δ, ppm: 8.06 (dd, J = 8.1, 1.1 Hz, 1H, H^{4}), 7.79 (tt, J = 5.6, 2.8 Hz, 1H, H^{2}), 7.66 (td, J = 8.1, 1.4 Hz, 1H, H^{3}), 7.57 (dd, J = 7.6, 1.3 Hz, 1H, H^{1}), 7.42 (d, J = 8.3 Hz, 1H, H^{9}), 7.30 (t, J = 7.5 Hz, 2H, H^{15}), 7.24 (t, J = 7.3 Hz, 1H, H^{16}), 7.14 (ddd, J = 8.2, 7.0, 1.1 Hz, 1H, H^{10}), 7.09 (d, J = 7.8 Hz, 1H, H^{12}), 7.04 – 6.98 (m, 3H, H^{14}, H^{11}), 5.54 (dd, J = 38.1, 17.3 Hz, 2H, H^{13}), 3.78 (dd, J = 47.7, 16.9 Hz, 2H, H^{7}), 3.43 (s, 3H, H^{17}). ^{13}C{H} NMR (151 MHz, DMSO-d_6), δ, ppm: 169.42 (Cq, C^{6}), 150.25 (Cq, C^{4a}), 137.65 (Cq, C^{13a}), 136.14 (Cq, C^{8a}), 133.50 (CH, C^{1}), 132.99 (CH, C^{2}), 130.83 (Cq, C^{7a}), 128.72 (CH, C^{3}), 128.52 (2CH, C^{15}), 128.05 (Cq, C^{12c}), 127.15 (CH, C^{16}), 126.44 (Cq, C^{12a}), 126.03 (2CH, C^{14}), 124.27 (CH, C^{4}), 122.07 (CH, C^{10}), 120.11 (CH, C^{11}), 117.78 (CH, C^{12}), 111.24 (Cq, C^{12b}), 110.54 (CH, C^{9}), 51.96 (CH_3, C^{17}), 46.38 (CH_2, C^{13}), 30.76 (CH_2, C^{7}). HRMS (ESI) m/z: $[M + Na]^+$ calcd for $C_{24}H_{20}N_2O_4Na$ 423.1315; found 423.1317. X-ray diffraction quality single crystals were obtained by slow evaporation of a methanolic solution of **1a**.

Methyl 2-(1-benzyl-5-bromo-3-(2-nitrophenyl)-1H-indol-2-yl)acetate (***1b***). A suspension of methyl 4-(2-nitrophenyl)-3-oxobutanoate (0.50 g, 2.11 mmol), 1-benzyl-1-(4-bromophenyl)hydrazine (0.87 g, 3.17 mmol), and sodium acetate (0.69 g, 8.29 mmol) in glacial acetic acid (10 mL) was stirred at 100 °C for 1 h. Then the mixture was cooled to room temperature, concentrated sulfuric acid (0.4 mL) was added and the solution was stirred at 100 °C for 1 h. After cooling to room temperature, the mixture was poured into a sat. aqueous sodium bicarbonate solution and extracted with ethyl acetate (3 × 70 mL). The organic phase was dried over magnesium sulfate and evaporated under reduced pressure. The residue was purified on a silica column using a mixture of hexane/ethyl acetate 9:1 as eluent to afford **1b** as a yellow solid. Yield: 0.54 g, 55%. ^{1}H NMR (600 MHz, DMSO-d_6), δ, ppm: 8.09 (d, J = 8.1 Hz, 1H, H^{4}), 7.80 (t, J = 7.6 Hz, 1H, H^{2}), 7.70 – 7.68 (m, 1H, H^{3}), 7.56 (d, J = 8.8 Hz, 1H, H^{1}), 7.43 (d, J = 8.8 Hz, 1H, H^{9}), 7.32 – 7.21 (m, 5H, H^{10}, H^{12}, H^{15}, H^{16}), 6.98 (d, J = 7.4 Hz, 2H, H^{14}), 5.56 (d, J = 10.5 Hz, 2H, H^{13}), 3.81 (d, J = 17.0 Hz, 1H, H^{7}), 3.72 (d, J = 17.0 Hz, 1H, H^{7}), 3.40 (s, 3H, H^{17}). ^{13}C{H} NMR (126 MHz, DMSO-d_6) δ, ppm: 169.11 (Cq, C^{6}) 150.15 (Cq, C^{4a}), 137.29 (Cq, C^{13a}), 134.92 (Cq, C^{8a}), 133.60 (CH, C^{1}), 133.17 (CH, C^{2}), 132.55 (Cq, C^{7a}), 129.13(CH, C^{3}), 128.59 (2CH, C^{15}), 128.29 (Cq, C^{12c}), 127.27 (CH, C^{16}), 126.01 (2CH, C^{14}), 124.57 (CH, C^{10}), 124.39 (CH, C^{4}), 120.08 (CH, C^{12}), 112.76 (CH, C^{9}), 112.73 (Cq, C^{11}), 110.90 (Cq, C^{12b}), 52.00 (CH_3, C^{17}), 46.59 (C_2, C^{13}), 30.77 (CH_2, C^{7}). HRMS (ESI) m/z: [M + H]$^{+}$ calcd for $C_{24}H_{20}N_2O_4Br$ 479.0601; found 479.0603.

Methyl 3-(1-benzyl-3-(2-nitrophenyl)-1H-indol-2-yl)propanoate (***1c***)*.* A suspension of methyl 5-(2-nitrophenyl)-4-oxopentanoate [5] (2.55 g, 10.15 mmol) and 1-benzyl-1-phenylhydrazine hydrochloride (2.34 g, 9.95 mmol) in glacial acetic acid (50 mL) was stirred at 100 °C for 2 h. After cooling to room temperature distilled water (125 mL) was added and the pH was adjusted to about 7 by adding sat. aqueous sodium bicarbonate solution. The resulting mixture was extracted with ethyl acetate (3 × 120 mL). The organic phase was dried over magnesium sulfate and evaporated under reduced pressure. The residue was purified on a silica column using a mixture of hexane/ethyl acetate 7:1 as eluent. Yield: 2.19 g, 53%. ^{1}H NMR (600 MHz, DMSO-d_6), δ, ppm: 8.05 (dd, J = 8.1, 1.1 Hz, 1H, H^{4}), 7.79 (td, J = 7.5, 1.3 Hz, 1H, H^{2}), 7.66 (td, J = 8.0, 1.4 Hz, 1H, H^{1}), 7.61 (dd, J = 7.6, 1.3 Hz, 1H, H^{3}), 7.38 (d, J = 8.3 Hz, 1H, H^{10}), 7.31 (t, J = 7.4 Hz, 2H, H^{16}), 7.24

(t, J = 7.3 Hz, 1H, H^{17}), 7.09 (ddd, J = 8.2, 7.0, 1.2 Hz, 1H, H^{11}), 7.05 (d, J = 7.6 Hz, 1H, H^{13}), 7.00 – 6.97 (m, 3H, H^{12}, H^{15}), 5.62 – 5.51 (m, 2H, H^{14}), 3.46 (s, 3H, H^{18}), 3.00 (ddd, J = 15.5, 9.7, 6.2 Hz, 1H, H^7), 2.94 – 2.86 (m, 1H, H^7), 2.44 – 2.35 (m, 2H, H^8). ^{13}C{H} NMR (151 MHz, DMSO), δ, ppm: 171.85 (Cq, C^6), 150.46 (Cq, C^{4a}), 138.15 (Cq, C^{14a}), 136.54 (Cq, C^{8a}), 136.11 (Cq, C^{9a}), 133.70 (CH, C^1), 132.94 (CH, C^2), 128.67 (2CH, C^{16}), 128.63 (CH, C^3), 128.55 (Cq, C^{13c}), 127.18 (CH, C^{17}), 126.82 (Cq, C^{13a}), 125.83 (2CH, C^{15}), 124.14 (CH, C^4), 121.69 (CH, C^{11}), 120.03 (CH, C^{12}), 117.50 (CH, C^{13}), 110.39 (CH, C^{10}), 109.55 (Cq, C^{13b}), 51.40 (CH_3, C^{18}), 46.05 (CH_2, C^{14}), 32.97 (CH_2, C^8), 19.86 (CH_2, C^7). HRMS (ESI) *m/z*: $[M + H]^+$ calcd for $C_{25}H_{23}N_2O_4$ 415.1652; found 415.1650.

*Methyl 2-(3-(2-aminophenyl)-1-benzyl-1H-indol-2-yl)acetate (**2a**)*. Methyl 2-(1-benzyl-3-(2-nitrophenyl)-1*H*-indol-2-yl)acetate (**1a**, 3.10 g, 7.75 mmol) and palladium (10%) on activated charcoal (825 mg, 0.78 mmol Pd) were suspended in dry methanol in a pressurization vessel. The vessel was flushed with hydrogen gas (3 × 5 bar) and then filled with H_2 to a pressure of 5 bar. The slurry was stirred at room temperature overnight. The next day, the reaction mixture was filtered over Celite®, followed by methanol washing. The filtrate was concentrated and the residue was purified on a silica column with hexane/ethyl acetate 4:1 as eluent to give **2a**. Yield: 2.64 g, 92%. A small amount of **3a** (59 mg, 2%) was obtained from the second collected fraction when hexane/ethyl acetate 1:1 was used as eluent. ^{1}H NMR (700 MHz, DMSO-d_6), δ, ppm: 7.39 (d, J = 8.2 Hz, 1H, H^9), 7.30 (t, J = 7.4 Hz, 2H, H^{15}), 7.27 – 7.22 (m, 2H, H^{16}, H^{12}), 7.12 (t, J = 7.5 Hz, 1H, H^{10}), 7.11 – 7.01 (m, 5H, H^{14}, H^1, H^3, H^{11}), 6.80 (d, J = 7.9 Hz, 1H, H^4), 6.65 (t, J = 7.2 Hz, 1H, H^2), 5.53 – 5.40 (m, 2H, H^{13}), 4.54 (s, 2H, H^5), 3.78 – 3.67 (m, 2H, H^7), 3.45 (s, 3H, H^{17}). ^{13}C{H} NMR (176 MHz, DMSO-d_6), δ, ppm: 170.66 (Cq, C^6), 146.99 (Cq, C^{4a}), 138.41 (Cq, C^{13a}), 137.14 (Cq, C^{8a}), 131.89 (CH, C^1), 131.11 (Cq, C^{7a}), 129.01 (2CH, C^{15}), 128.49 (CH, C^9), 127.58 (CH, C^{16}), 127.03 (Cq, C^{12a}), 126.76 (2CH, C^{14}), 122.17 (CH, C^{10}), 119.96 (CH, C^{11}), 119.66 (CH, C^{12}), 118.39 (Cq, C^{12c}), 116.78 (CH, C^2), 115.17 (CH, C^4), 113.25 (Cq, C^{12b}), 110.75 (CH, C^9), 52.37 (CH_3, C^{17}), 47.01 (CH_2, C^{13}), 31.42 (CH_2, C^7). HRMS (ESI) *m/z*: $[M + H]^+$ calcd for $C_{24}H_{23}N_2O_2$ 371.1754; found 371.1757.

*Methyl 3-(3-(2-aminophenyl)-1-benzyl-1H-indol-2-yl)propanoate (**2c**).* Compound **1c** (380 mg, 0.92 mmol) and palladium (10%) on activated charcoal (100 mg, 0.09 mmol Pd)

were suspended in dry methanol in a pressurization vessel. The vessel was flushed with hydrogen gas (3 × 4 bar) and then filled to a pressure of 5 bar. The reaction mixture was stirred at room temperature overnight. The next day, the reaction mixture was filtered over Celite®, followed by methanol washing. The filtrate was concentrated and the residue was purified on a silica column with hexane/ethyl acetate 6:1 as eluent. Yield: 350 mg, 99%. ^{1}H NMR (600 MHz, DMSO-d_6), δ, ppm: 7.35 (d, J = 8.2 Hz, 1H, H^{10}), 7.32 – 7.30 (m, 2H, H^{16}), 7.26 – 7.23 (m, 1H, H^{17}), 7.16 (d, J = 7.8 Hz, 1H, H^{13}), 7.11 – 7.03 (m, 4H, H^3, H^{11}, H^{15}), 7.03 – 6.97 (m, 2H, H^1, H^{12}), 6.79 (dd, J = 8.0, 0.9 Hz, 1H, H^4), 6.64 (td, J = 7.4, 1.1 Hz, 1H, H^2), 5.52 (s, 2H, H^{14}), 4.51 (s, 2H, H^5), 3.47 (s, 3H, H^{18}), 2.99 – 2.87 (m, 2H, H^7), 2.38 (ddd, J = 18.9, 10.1, 6.3 Hz, 2H, H^8). ^{13}C{H} NMR (151 MHz, DMSO), δ, ppm: 172.11 (Cq, C^6), 146.62 (Cq, C^{13a}), 138.46 (Cq, C^{14a}), 136.49 (Cq, C^{8a}), 136.39 (Cq, C^{9a}), 131.46 (CH, C^1), 128.65 (2CH, C^{16}), 127.93 (CH, C^3), 127.08 (CH, C^{17}), 127.06 (Cq, C^{13a}), 126.01 (2CH, C^{15}), 121.16 (CH, C^{11}), 119.32 (CH, C^{12}), 118.68 (CH, C^{13}), 118.52 (Cq, C^{13c}), 116.25 (CH, C^2), 114.53 (CH, C^4), 111.00 (Cq, C^{13b}), 110.06 (Cq, C^{10}), 51.31 (CH$_3$, C^{18}), 46.08 (CH$_2$, C^{14}), 33.09 (CH$_2$, C^8), 20.31 (CH$_2$, C^7). HRMS (ESI) *m/z*: [M + H]$^+$ calcd for $C_{25}H_{25}N_2O_2$ 385.1911; found 385.1911. X-ray diffraction quality crystals were obtained by slow evaporation of ethyl acetate solution of **2c**.

8-Benzyl-7-hydroindolo[2,3-d][1]benzazepin-6(5H)-one ***(3a)*****.** A solution of trimethylaluminum (2 M in toluene, 14.2 mL, 28.4 mmol) in dry dichloromethane (60 mL) under argon atmosphere was cooled to −10 °C by using a 3:1 mixture of ice and sodium chloride. A solution of methyl 2-(3-(2-aminophenyl)-1-benzyl-1*H*-indol-2-yl)acetate (**2a**, 2.63 g, 7.10 mmol) in dry dichloromethane (10 mL) was added dropwise. Then the mixture was allowed to slowly reach room temperature and stirred overnight. On the next day, the reaction mixture was poured onto a mixture of ice and 1 M HCl (200 mL). The organic layer was separated, and the aqueous phase was extracted with dichloromethane (2 × 200 mL). The combined organic layers were washed with saturated bicarbonate solution and brine, dried over magnesium sulfate, and evaporated. The residue was crystallized from methanol (15 mL) to give the product as white needles. Yield: 1.91 g, 80%. ^{1}H NMR (600 MHz, DMSO-d_6), δ, ppm: 10.15 (s, 1H, H^5), 7.92 – 7.87 (m, 1H, H^1), 7.85 (d, J = 7.5 Hz, 1H, H^{12}), 7.57 (d, J = 8.0 Hz, 1H, H^9), 7.33 – 7.25 (m, 5H, H^{15}, H^3, H^2, H^4), 7.23 (t, J

= 7.3 Hz, 1H, H^{16}), 7.21 – 7.14 (m, 4H, H^{14}, H^{10}, H^{11}), 5.60 (s, 2H, H^{13}), 3.65 (s, 2H, H^{7}). ^{13}C{H} NMR (151 MHz, DMSO-d_6), δ, ppm: 169.15 (Cq, C^{6}), 137.79 (Cq, C^{13a}), 137.04 (Cq, C^{8a}), 134.37 (Cq, C^{4a}), 132.21 (Cq, C^{7a}), 128.55 (2CH, C^{15}) 127.82 (CH, C^{1}), 127.27 (CH, C^{16}), 126.67 (2CH, C^{14}), 125.98 (CH, C^{3}), 125.88 (Cq, C^{12c}), 124.45 (Cq, C^{12a}), 124.13 (CH, C^{4}), 122.53 (CH, C^{2}), 121.76 (CH, C^{10}), 120.55 (CH, C^{11}), 118.19 (CH, C^{12}), 111.04 (CH, C^{9}), 109.98 (Cq, C^{12b}), 45.81 (CH_2, C^{13}), 32.96 (CH_2, C^{7}). HRMS (ESI) *m/z*: $[M + Na]^+$ calcd for $C_{23}H_{18}N_2ONa$ 361.1311; found 361.1307. X-ray diffraction quality crystals were obtained by slow evaporation of a methanolic solution of **3a**.

8-Benzyl-11-bromo-7-hydroindolo[2,3-d][1]benzazepin-6(5H)-one (***3b***). Methyl 2-(1-benzyl-5-bromo-3-(2-nitrophenyl)-1*H*-indol-2-yl)acetate (370 mg, 0.77 mmol) was dissolved in a 6:1 mixture of ethanol and acetic acid (16.3 mL) and iron powder (218 mg, 3.85 mmol) was added. The mixture was heated at 80 °C for 2 h. The solution was cooled to room temperature, poured into ice water, and the pH was adjusted to about 10 by addition of sat. aqueous potassium carbonate solution. The mixture was filtered over Celite®, washed with water (100 mL) and ethyl acetate (100 mL). The organic layers were combined, dried over magnesium sulfate, and concentrated under reduce pressure. The residue was purified on a silica column using a mixture of hexane/ethyl acetate 6:1 as eluent. Yield: 185 mg, 58%. ^{1}H NMR (600 MHz, DMSO-d_6), δ, ppm: 10.20 (s, 1H, H^{5}), 7.95 (d, J = 1.8 Hz, 1H, H^{12}), 7.86 (d, J = 9.3 Hz, 1H, H^{1}), 7.57 (d, J = 8.7 Hz, 1H, H^{9}), 7.34 – 7.20 (m, 7H, H^{2}, H^{3}, H^{4}, H^{10}, H^{15}, H^{16}), 7.15 (d, J = 7.1 Hz, 2H, H^{14}), 5.62 (s, 2H, H^{13}), 3.68 (s, 2H, H^{7}). ^{13}C{H} NMR (126 MHz, DMSO-d_6) *δ,* ppm: δ 169.05 (Cq, C^{6}), 137.49 (Cq, C^{13a}), 135.85 (Cq, C^{8a}), 134.51 (Cq, C^{4a}), 133.78 (Cq, C^{7a}), 128.65 (2CH, C^{15}), 127.86 (CH, C^{1}), 127.42 (CH, C^{16}), 126.65 (2CH, C^{14}), 126.42 (CH, C^{3}), 126.12 (Cq, C^{12a}), 125.16 (Cq, C^{12c}), 124.39 (CH, C^{4}), 124.29 (CH, C^{10}), 122.61 (CH, C^{2}), 120.39 (CH, C^{12}), 113.29 (CH, C^{11}), 113.22 (CH, C^{9}), 109.70 (Cq, C^{12b}), 46.00 (CH_2, C^{13}), 33.05 (CH_2, C^{7}). HRMS (ESI) *m/z*: $[M + Na]^+$ calcd for $C_{23}H_{17}N_2OBrNa$ 439.0416; found 439.0409.

*9-Benzyl-7,8-dihydroindolo[2,3-e][1]benzazocin-6(5H)-one (**3c**)*. A solution of trimethyl aluminum (2 M in toluene, 2.28 mL, 4.55 mmol) in dry dichloromethane (10 mL) under argon atmosphere was cooled to −10 °C using a 3:1 mixture of ice and sodium chloride.

Methyl 3-(3-(2-aminophenyl)-1-benzyl-1*H*-indol-2-yl)propanoate (**2c**, 350 mg, 0.91 mmol) was dissolved in dry dichloromethane (10 mL) and added dropwise. Then the mixture was allowed to slowly reach room temperature and stirred overnight. On the next day, the mixture was poured on a mixture of ice and 1 M HCl. The organic layer was separated, and the aqueous layer was extracted with dichloromethane (2 × 50 mL). The combined organic layers were washed with saturated bicarbonate solution and brine, dried over magnesium sulfate, and evaporated. The residue was purified on a silica column using a mixture of hexane/ethyl acetate 3:1 as eluent. Yield: 171 mg, 53%. ^{1}H NMR (600 MHz, DMSO-d_6): δ 9.67 (s, 1H, H^5), 7.54 (dd, J = 7.3, 1.9 Hz, 1H, H^1), 7.45 (d, J = 8.2 Hz, 1H, H^{10}), 7.41 – 7.35 (m, 2H, H^4, H^{17}), 7.30 (t, J = 7.3 Hz, 2H, H^{16}), 7.28 – 7.25 (m, 2H, H^3, H^{13}), 7.23 – 7.20 (m, 1H, H^2), 7.14 – 7.10 (m, 1H, H^{11}), 7.04 (d, J = 8.1 Hz, 2H, H^{15}), 7.03 – 6.99 (m, 1H, H^{12}), 5.50 (q, J = 17.2 Hz, 2H, H^{14}), 3.27 – 3.23 (m, 1H, H^7), 3.13 – 3.05 (m, 1H, H^7), 2.82 – 2.79 (m, 1H, H^8), 2.17 – 2.11 (m, 1H, H^8). ^{13}C{H} NMR (151 MHz, DMSO-d_6), δ, ppm: δ 173.26 (Cq, C^6), 137.98 (Cq, C^{14a}), 136.56 (Cq, C^{9a}), 136.26 (Cq, C^{4a}), 135.46 (Cq, C^{8a}), 132.42 (CH, C^1), 131.09 (Cq, C^{13c}), 128.65 (2CH, C^{16}), 127.91 (Cq, C^{13a}), 127.16 (CH, C^{17}), 127.08 (CH, C^3), 126.21 (2CH, C^{15}), 125.97 (CH, C^4), 125.55 (CH, C^2), 121.75 (CH, C^{11}), 119.77 (CH, C^{12}), 118.18 (CH, C^{13}), 109.87 (Cq, C^{13b}), 109.78 (CH, C^{10}), 45.74 (CH_2, C^{14}), 28.53 (CH_2, C^8), 23.92 (CH_2, C^7). HRMS (ESI) *m/z*: $[M + Na]^+$ calcd for $C_{24}H_{20}N_2ONa$ 375.1468; found 375.1458.

*7,8-Dihydroindolo[2,3-d][1]benzazepin-6(5H)-one (**C**).* A suspension of 8-benzyl-7-hydroindolo[2,3-*d*][1]benzazepin-6(5*H*)-one (**3a**, 635 mg, 1.88 mmol) in dry benzene (32 mL) was added dropwise to a suspension of aluminum chloride (1.25 g, 9.38 mmol) in dry benzene (32 mL) at boiling temperature. Then the reaction mixture was stirred under reflux for 5 min and cooled to room temperature. The resulting mixture was poured on a mixture of ice and 2 M aqueous hydrochloric acid (ca. 150 mL) and extracted with dichloromethane (4 × 100 mL). The organic phase was washed with saturated sodium bicarbonate solution and dried over magnesium sulfate. The organic phase was concentrated to a volume of ca. 30 mL and allowed to stand at room temperature for 1 h. Then, the white precipitate was filtered off, washed with benzene, and dried *in vacuo*. Yield: 333 mg, 71%. ^{1}H NMR (500 MHz, DMSO-d_6) δ 11.58 (s, 1H, H^8), 10.09 (s, 1H, H^5),

7.88 – 7.84 (m, 1H, H^1), 7.81 (d, J = 7.7 Hz, 1H,H^{12}), 7.45 (d, J = 7.8 Hz, 1H, H^9), 7.30 – 7.22 (m, 3H, H^4, H^3, H^2), 7.20 – 7.08 (m, 2H, H^{10}, H^{11}), 3.55 (s, 2H, H^7). ^{13}C{H} NMR (126 MHz, DMSO-d_6) δ 170.02 (Cq, C^6), 136.64 (Cq, C^{8a}), 134.62 (Cq, C^{4a}), 132.01 (Cq, C^{7a}), 127.94 (CH, C^1), 126.72 (Cq, C^{12c}), 126.09 (CH, C^3), 125.68 (Cq, C^{12a}), 124.58 (CH, C^4), 123.06 (CH, C^2), 121.85 (CH, C^{10}), 120.48 (CH, C^{11}), 118.44 (CH, C^{12}), 112.25 (CH, C^9), 109.53 (Cq, C^{12b}), 35.53 (CH_2, C^7). HRMS (ESI) *m*/*z*: $[M + Na]^+$ calcd for $C_{16}H_{12}N_2ONa$ 271.0842; found 271.0839.

Synthesis of compounds 4–24

1-Benzyl-1-(4-bromophenyl)hydrazine. 4-Bromophenyl hydrazine (4.47 g, 20 mmol) was suspended in dry acetonitrile (66 mL) and cooled to 0 °C. Sodium hydride (60% dispersion in mineral oil, 1.68 g, 42 mmol) was added and the mixture was stirred for 1.5 h at 0 °C. Benzyl bromide (2.53 mL, 21.3 mmol) was mixed with dry acetonitrile (33 mL) and added dropwise at 0 °C. After complete addition, the solution was allowed to slowly reach room temperature and stirred overnight. On the next day the reaction mixture was poured on water (100 mL) and extracted with dichloromethane (3 × 150 mL). The organic phase was dried over magnesium sulfate and evaporated under reduced pressure. The residue was purified on a silica column using a mixture of hexane/ethyl acetate 9:1 as eluent. Yield: 2.32 g, 42%. ^{1}H NMR (500 MHz, DMSO) δ, ppm: δ 7.33 – 7.29 (m, 2H, H_{Ar}), 7.28 – 7.20 (m, 5H, H_{Ar}), 6.91 (d, J = 9.1 Hz, 2H, H_{Ar}), 4.62 (s, 2H, CH_2), 4.45 (s, 2H, NH_2). HRMS (ESI) *m/z*: $[M + H]^+$ calcd for $C_{13}H_{14}N_2Br$ 277.0335; found 277.0327. Preparation of 1-Benzyl-1-(4-bromophenyl)hydrazine by a different method has been described in the literature; NMR data was reported in $CDCl_3$.[6]

*1-(Ethoxymethyl)-1H-indole (**4**).* A solution of 1*H*-indole (1.24 g, 10.6 mmol) in dry dimethylformamide (21 mL) was cooled to 0 °C and sodium hydride (60% dispersion in mineral oil, 636 mg, 15.9 mmol) was added in portions. The mixture was stirred at 0 °C for 10 min and at room temperature for 1 h. Afterwards, chloromethyl ethyl ether (1.97 mL, 21.2 mmol) was added dropwise and the resulting mixture was stirred at room temperature overnight. On the next day, the solvent was removed under reduced pressure. The residue was taken up in water (20 mL) and extracted with dichloromethane

(3 × 20 mL). The organic phase was dried over magnesium sulfate and evaporated under reduced pressure. The residue was purified on a silica column using a mixture of hexane/ethyl acetate 95:5 as eluent. Yield: 1.56 g, 84%. ^{1}H NMR (500 MHz, DMSO-d_6), δ, ppm: 7.59 – 7.53 (m, 2H, H_{Ar}), 7.46 (d, J = 3.2 Hz, 1H, H_{Ar}), 7.16 (ddd, J = 8.2, 7.1, 1.0 Hz, 1H, H_{Ar}), 7.06 (ddd, J = 7.9, 7.1, 0.9 Hz, 1H, H_{Ar}), 6.48 (dd, J = 3.2, 0.7 Hz, 1H, H_{Ar}), 5.55 (s, 2H, CH_2OCH$_2$CH$_3$), 3.38 (q, J = 7.0 Hz, 2H, CH$_2$OCH_2CH$_3$), 1.04 (t, J = 7.0 Hz, 3H, CH$_2$OCH$_2$CH_3). Preparation of compound **4** by a slightly different method has been described in the literature; however, NMR data was reported in CDCl$_3$.[7]

*1-(Ethoxymethyl)-3-(2-nitrophenyl)-1H-indole (**5**).* A mixture of 1-(ethoxymethyl)-1*H*-indole (**4**, 6.27 g, 35.8 mmol), *o*-iodo-nitrobenzene (8.92 g, 35.8 mmol), palladium(II) acetate (804 mg, 3.58 mmol), and potassium carbonate (14.84 g, 107.4 mmol) in dry dioxane was stirred at 100 °C overnight. After cooling to room temperature, the mixture was filtered over Celite® and the filtrate was evaporated. The residue was purified on a silica column using a mixture of hexane/ethyl acetate 9:1 as eluent. Yield: 2.35 g, 22%. ^{1}H NMR (500 MHz, DMSO-d_6) , δ, ppm: 8.15 (dd, J = 8.2, 1.2 Hz, 1H, H_{Ar}), 7.88 – 7.83 (m, 1H, H_{Ar}), 7.79 – 7.75 (m, 1H, H_{Ar}), 7.73 – 7.68 (m, 1H, H_{Ar}), 7.66 (dd, J = 8.3, 0.6 Hz, 1H, H_{Ar}), 7.59 (d, J = 7.8 Hz, 1H, H_{Ar}), 7.24 (ddd, J = 8.3, 7.1, 1.2 Hz, 1H, H_{Ar}), 7.15 – 7.11 (m, 1H, H_{Ar}), 6.55 (d, J = 0.5 Hz, 1H, H_{Ar}), 5.34 (s, 2H, CH_2OCH$_2$CH$_3$), 3.29 (q, J = 7.0 Hz, 2H, CH$_2$OCH_2CH$_3$), 0.96 (t, J = 7.0 Hz, 3H, CH$_2$OCH$_2$CH_3).

*2-(1-(Ethoxymethyl)-1H-indol-3-yl)aniline (**6**).* A suspension of 1-(ethoxymethyl)-3-(2-nitrophenyl)-1*H*-indole (**5**, 1.00 g, 3.37 mmol) and palladium (10%) on activated charcoal (359 mg, 0.34 mmol Pd) in dry tetrahydrofuran was transferred into a pressurization vessel. The vessel was flushed with hydrogen (3 × 4 bar) and then filled with hydrogen (4 bar). The reaction mixture was stirred at room temperature overnight. On the next day, the suspension was filtered over Celite® and the filtrate was evaporated. The residue was purified on a silica column using a mixture of hexane/ethyl acetate 9:1 as eluent. Yield: 700 mg, 78%. ^{1}H NMR (500 MHz, DMSO-d_6) , δ, ppm: 7.59 – 7.53 (m, 2H, H_{Ar}), 7.21 – 7.06 (m, 4H, H_{Ar}), 6.79 (d, J = 7.9 Hz, 1H, H_{Ar}), 6.65 (t, J = 7.4 Hz, 1H, H_{Ar}), 6.47 (s, 1H,

H_{Ar}), 5.36 (s, 2H, $CH_2OCH_2CH_3$), 4.92 (s, 2H, NH_2), 3.18 (q, J = 7.0 Hz, 2H, $CH_2OCH_2CH_3$), 0.89 (t, J = 7.0 Hz, 3H, $CH_2OCH_2CH_3$).

*2-Chloro-N-(2-(1-(ethoxymethyl)-1H-indol-3-yl)phenyl)acetamide (**7**).* A solution of 2-(1-(ethoxymethyl)-1*H*-indol-3-yl)aniline (**6**, 226 mg, 0.85 mmol) and triethylamine (165 µL, 1.19 mmol) in dry dichloromethane (5 mL) was cooled to 0 °C and a solution of chloroacetyl chloride (95 µL, 1.19 mmol) in dry dichloromethane (5 mL) was added dropwise. Afterwards, the reaction mixture was allowed to slowly reach room temperature and stirred for 48 h. The reaction was quenched with water (10 mL) and extracted with dichloromethane (3 × 10 mL). The combined organic phases were dried over magnesium sulfate and evaporated. The residue was purified on a silica column using a mixture of hexane/ethyl acetate first 9:1 then 8:2 as eluent. Yield: 230 mg, 79%. ^{1}H NMR (500 MHz, DMSO-d_6), δ, ppm: 9.41 (s, 1H, NH), 7.84 (d, J = 8.0 Hz, 1H, H_{Ar}), 7.63 – 7.58 (m, 2H, H_{Ar}), 7.52 – 7.46 (m, 2H, H_{Ar}), 7.34 (dd, J = 10.9, 4.1 Hz, 1H, H_{Ar}), 7.25 – 7.20 (m, 1H, H_{Ar}), 7.13 (dd, J = 11.0, 4.0 Hz, 1H, H_{Ar}), 6.58 (s, 1H, H_{Ar}), 5.29 (s, 2H, $CH_2OCH_2CH_3$), 4.17 (s, 2H, CH_2Cl), 3.21 (q, J = 7.0 Hz, 2H, $CH_2OCH_2CH_3$), 0.93 (t, J = 7.0 Hz, 3H, $CH_2OCH_2CH_3$).

*N-Benzyl-2-chloro-N-(2-(1-(ethoxymethyl)-1H-indol-3-yl)phenyl)acetamide (**8**).* A solution of 2-chloro-*N*-(2-(1-(ethoxymethyl)-1*H*-indol-3-yl)phenyl)acetamide (**7**, 230 mg, 0.67 mmol) in dry acetonitrile (5 mL) was cooled to 0 °C and sodium hydride (60% dispersion in mineral oil, 38 mg, 0.94 mmol) was added. The resulting mixture was stirred at 0 °C for 10 min and a solution of benzyl bromide (88 µL, 0.74 mmol) in dry acetonitrile (5 mL) was added dropwise. The reaction mixture was allowed to slowly reach room temperature and stirred overnight. On the next day, the reaction was quenched with saturated aqueous ammonium chloride solution (20 mL) and extracted with dichloromethane (3 × 20 mL). The combined organic layers were dried over magnesium sulfate and evaporated. The residue was purified on a silica column using a mixture of hexane/ethyl acetate first 9:1 then 8:2 as eluent. Yield: 255 mg, 88%. ^{1}H NMR (500 MHz, DMSO-d_6), δ, ppm: 7.71 – 7.64 (m, 2H, H_{Ar}), 7.61 (d, J = 7.8 Hz, 1H, H_{Ar}), 7.55 (td, J = 7.6, 1.1 Hz, 1H, H_{Ar}), 7.44 (td, J = 7.8, 1.5 Hz, 1H, H_{Ar}), 7.26 – 7.08 (m, 8H, H_{Ar}), 6.55 (s,

1H, H_{Ar}), 5.46 (d, J = 11.0 Hz, 1H, $CH_2OCH_2CH_3$), 5.37 (d, J = 11.0 Hz, 1H, $CH_2OCH_2CH_3$), 5.08 (d, J = 14.7 Hz, 1H, $CH_2C_6H_5$), 4.30 (d, J = 13.7 Hz, 1H, CH_2Cl), 4.11 (d, J = 13.7 Hz, 1H, CH_2Cl), 3.79 (d, J = 14.7 Hz, 1H, $CH_2C_6H_5$), 3.51 – 3.40 (m, 2H, $CH_2OCH_2CH_3$ (overlapped water peak)), 0.98 (t, J = 7.0 Hz, 3H, $CH_2OCH_2CH_3$).

*N-Benzyl-N-(2-(1-(ethoxymethyl)-1H-indol-3-yl)phenyl)-2-iodoacetamide (**9**).* A suspension of *N*-benzyl-2-chloro-*N*-(2-(1-(ethoxymethyl)-1*H*-indol-3-yl)phenyl)acetamide (**8**, 216 mg, 0.50 mmol) and sodium iodide (748 mg, 4.99 mmol) in acetone (6 mL) was stirred at room temperature overnight. On the next day, the reaction mixture was diluted with ethyl acetate (50 mL) and washed with 10% aqueous sodium thiosulfate solution (2 × 20 mL). The organic phase was dried over magnesium sulfate and evaporated. The residue was purified on a silica column using a mixture of hexane/ethyl acetate first 9:1 then 8:2 as eluent. Yield: 208 mg, 79%. ^{1}H NMR (500 MHz, DMSO-d_6), δ, ppm: 7.70 – 7.64 (dd, J = 12.5, 4.8 Hz, 2H, H_{Ar}), 7.60 (d, J = 7.8 Hz, 1H, H_{Ar}), 7.55 (td, J = 7.6, 1.2 Hz, 1H, H_{Ar}), 7.45 (td, J = 7.7, 1.5 Hz, 1H, H_{Ar}), 7.28 – 7.21 (m, 4H, H_{Ar}), 7.18 – 7.08 (ddd, J = 10.5, 9.3, 4.3 Hz, 4H, H_{Ar}), 6.60 (s, 1H, H_{Ar}), 5.43 (d, J = 11.0 Hz, 1H, $CH_2OCH_2CH_3$), 5.36 (d, J = 11.0 Hz, 1H, $CH_2OCH_2CH_3$), 5.09 (d, J = 14.7 Hz, 1H, $CH_2C_6H_5$), 3.82 – 3.75 (m, 2H, $CH_2C_6H_5$, CH_2I), 3.63 (d, J = 10.5 Hz, 1H, CH_2I), 3.48 – 3.40 (m, 2H, $CH_2OCH_2CH_3$), 0.99 (t, J = 7.0 Hz, 3H, $CH_2OCH_2CH_3$).

*(1H-Indole-2-yl)methanol (**10**).* The synthesis was carried out by following a modified literature procedure [8]. A solution of indole-2-carboxylate (6.00 g, 31.71 mmol) in dry tetrahydrofuran (100 mL) was added dropwise to a suspension of lithium aluminum hydride (1.80 g, 47.56 mmol) in dry tetrahydrofuran (42 mL) at 0 °C. The resulting mixture was refluxed for 4 h, then cooled to 0 °C, and water (5 mL) was added slowly, followed by a solution of sodium hydroxide (0.75 g, 18.75 mmol) in water (5 mL). The mixture was stirred at room temperature for 1 h. Then, the precipitate was filtered off and washed with tetrahydrofuran. The filtrate was dried over magnesium sulfate and concentrated under reduced pressure. Yield: 3.60 g, 89%. ^{1}H NMR (500 MHz, DMSO-d_6), δ, ppm: 10.99 (s, 1H, NH), 7.44 (d, J = 7.8 Hz, 1H, H_{Ar}), 7.31 (dd, J = 8.1, 0.7 Hz, 1H, H_{Ar}), 7.05 – 6.98 (m, 1H, H_{Ar}), 6.95 – 6.90 (m, 1H, H_{Ar}), 6.25 (d, J = 1.1 Hz, 1H, H_{Ar}), 5.23 (t, J = 5.5 Hz, 1H,

CH_2OH), 4.59 (d, *J* = 5.2 Hz, 2H, CH_2OH). Preparation of compound **10** by a slightly different method has been described in the literature; however, NMR data was reported in $CDCl_3$.[9]

*(1H-Indol-2-yl)methyl benzoate (**11**).* The compound was prepared by following a reported procedure [10]. Yield: 6.79 g, 89%. ^1H NMR (500 MHz, DMSO-d_6), *δ*, ppm: 11.33 (s, 1H, N*H*), 8.01 (dd, *J* = 8.3, 1.2 Hz, 2H, H_{Ar}), 7.70 – 7.64 (m, 1H, H_{Ar}), 7.54 – 7.52 (m, 3H, H_{Ar}), 7.40 – 7.35 (m, 1H, H_{Ar}), 7.14 – 7.07 (m, 1H, H_{Ar}), 7.02 – 6.96 (m, 1H, H_{Ar}), 6.55 (d, *J* = 1.3 Hz, 1H, H_{Ar}), 5.46 (s, 2H, CH_2). Preparation of compound **11** has been described in the literature; however, NMR data was reported in $CDCl_3$.[10]

*2-(1H-Indol-2-yl)acetonitrile (**12**).* The compound was synthesized as reported in the literature [11]. Yield: 2.24 g, 53%. ^1H NMR (500 MHz, DMSO-d_6), *δ*, ppm: 11.29 (s, 1H, N*H*), 7.49 (d, *J* = 7.8 Hz, 1H, H_{Ar}), 7.34 (dd, *J* = 8.1, 0.7 Hz, 1H, H_{Ar}), 7.13 – 7.04 (m, 1H, H_{Ar}), 7.03 – 6.93 (m, 1H, H_{Ar}), 6.37 (d, *J* = 1.1 Hz, 1H, H_{Ar}), 4.18 (d, *J* = 0.5 Hz, 2H, CH_2). Preparation of compound **12** by a slightly different method has been described in the literature; however, NMR data was reported in $CDCl_3$.[11]

*Methyl indol-2-ylacetate (**13**).* The synthesis was carried out by following a modified literature procedure [12]. A solution of 2-(1*H*-indol-2-yl)acetonitrile (**12**, 1.10 g, 7.04 mmol) in dry methanol (40 mL) was cooled to 0 °C and a stream of gaseous hydrogen chloride was bubbled through the solution for 20 min. Then the mixture was stirred at room temperature for 48 h. Afterwards, the mixture was neutralized using saturated aqueous sodium bicarbonate solution and extracted with dichloromethane (5 × 60 mL). The combined organic layers were dried over magnesium sulfate and concentrated under reduced pressure. The residue was purified on a silica column by using a mixture of hexane/ethyl acetate 3:1 as eluent. Yield: 1.10 g, 83%. ^1H NMR (500 MHz, DMSO-d_6), *δ*, ppm: 11.03 (s, 1H, N*H*), 7.44 (d, *J* = 7.9 Hz, 1H, , H_{Ar}), 7.31 (dd, *J* = 8.1, 0.8 Hz, 1H, H_{Ar}), 7.03 (ddd, *J* = 8.1, 7.1, 1.2 Hz, 1H, H_{Ar}), 6.94 (td, *J* = 7.5, 1.0 Hz, 1H, H_{Ar}), 6.26 (d, *J* = 1.2 Hz, 1H, H_{Ar}), 3.84 (s, 2H, CH_2), 3.65 (s, 3H, CH_3). Preparation of compound **13** by a different method was described in the literature; NMR data was reported in $CDCl_3$ [13].

*2-(1H-Indol-2-yl)acetic acid (**14**).* The compound was prepared by adapting the procedure reported for a similar acid [14]. Methyl indol-2-ylacetate (**13**, 4.08 g, 21.56 mmol) was dissolved in a mixture of tetrahydrofuran/water/methanol 1:1:1 (120 mL) and lithium hydroxide monohydrate (1.69 g, 41.96 mmol) was added. The resulting reaction mixture was stirred at room temperature for 16 h and then diluted with water (80 mL), acidified with 1 M hydrochloric acid to pH ≈ 1 and extracted with ethyl acetate (3 × 60 mL). The combined organic phases were washed with water and brine, concentrated under reduced pressure to give a pale-beige solid. Yield: 3.60 g, 95%. ^{1}H NMR (500 MHz, DMSO-d_6), δ, ppm: 12.50 (s, 1H, COO*H*), 10.99 (s, 1H, N*H*), 7.43 (d, *J* = 7.8 Hz, 1H, H_{Ar}), 7.30 (dd, *J* = 8.0, 0.8 Hz, 1H, H_{Ar}), 7.01 (ddd, *J* = 8.1, 7.1, 1.2 Hz, 1H, H_{Ar}), 6.95 – 6.91 (m, 1H, H_{Ar}), 6.24 (d, *J* = 1.2 Hz, 1H, H_{Ar}), 3.72 (s, 2H, CH_2). Analytical data are in accordance with those reported in the literature.[13]

*2-(1H-Indol-2-yl)-N-(2-iodophenyl)acetamide (**15**).* The compound was prepared by adapting a procedure reported for a similar amide [15]. Under argon atmosphere 1-[bis(dimethylamino)methylene]-1*H*-1,2,3-triazolo[4,5-*b*]pyridinium 3-oxide hexafluoro-phosphate (HATU, 1.65 g, 4.34 mmol) was added to a solution of *N*,*N*-diisopropylethylamine (DIPEA, 1,35 mL, 7.81 mmol) and 2-(1*H*-indol-2-yl)acetic acid (**14**, 0.66 g, 3.77 mmol) in dry dimethylformamide (5 mL). After 6 min, 2-iodoaniline (0.75 g, 3.47 mmol) was added. The reaction mixture was stirred at room temperature overnight. On the next day the reaction mixture was diluted with water (5 mL) and extracted with chloroform (5 × 20 mL). The organic phases were combined, dried over magnesium sulfate, and concentrated under reduced pressure. The residue was purified on a silica column by using a mixture of hexane/ethyl acetate 3:1 as eluent. Yield: 0.32 g, 23%. ^{1}H NMR (500 MHz, DMSO-d_6), δ, ppm: 11.04 (s, 1H, N*H*), 9.51 (s, 1H, CON*H*), 7.87 (d, *J* = 7.0 Hz, 1H, H_{Ar}), 7.52 (d, *J* = 6.8 Hz, 1H, H_{Ar}), 7.45 (d, *J* = 7.8 Hz, 1H, H_{Ar}), 7.38 (t, *J* = 7.1 Hz, 1H, H_{Ar}), 7.33 (d, *J* = 8.1 Hz, 1H, H_{Ar}), 7.05 – 7.00 (m, 1H, H_{Ar}), 6.95 (ddd, *J* = 14.9, 10.9, 3.9 Hz, 2H, H_{Ar}), 6.36 (s, 1H, H_{Ar}), 3.87 (s, 2H, CH_2).

*tert-Butyl 2-(2-((tert-butoxycarbonyl)(2-iodophenyl)amino)-2-oxoethyl)-1H-indole-1-carboxylate (**16**).* To a solution of 2-(1*H*-indol-2-yl)-*N*-(2-iodophenyl)acetamide (**15**, 550 mg, 1.46 mmol) in dry acetonitrile (27 mL) under argon atmosphere 4-dimethylaminopyridine (178 mg, 1.46 mmol) and di-*tert*-butyl dicarbonate (573 mg, 2.63 mmol) were added. The mixture was stirred at room temperature for 12 h. Then the solvent was removed under reduced pressure. The crude solid was washed with water and extracted with ethyl acetate (3 × 20 mL). The organic phases were combined, dried over magnesium sulfate, and concentrated under reduced pressure. The residue was purified on a silica column by using a mixture of hexane/ethyl acetate 3:1 as eluent. Yield: 723 mg, 86%. ^{1}H NMR (500 MHz, DMSO-d_6), δ, ppm: 7.98 (d, J = 8.6 Hz, 1H, H_{Ar}), 7.91 (dd, J = 7.9, 1.3 Hz, 1H, H_{Ar}), 7.53 (d, J = 7.3 Hz, 1H, H_{Ar}), 7.45 (td, J = 7.7, 1.4 Hz, 1H, H_{Ar}), 7.26 – 7.23 (m, 2H, H_{Ar}), 7.22 – 7.18 (m, 1H, H_{Ar}), 7.11 (td, J = 7.7, 1.5 Hz, 1H, H_{Ar}), 6.69 (s, 1H, H_{Ar}), 4.82 (d, J = 18.1 Hz, 1H, CH_2), 4.56 (d, J = 18.2 Hz, 1H, CH_2), 1.63 (s, 9H, (CH_3)$_3$), 1.38 (s, 9H, (CH_3)$_3$).

*N-(Ethoxymethyl)-2-[1-(ethoxymethyl)-1H-indole-2-yl]-N-(2-iodophenyl)acetamide (**17**).* To a solution of 2-(1*H*-indol-2-yl)-*N*-(2-iodophenyl)acetamide (**15**, 20 mg, 0.05 mmol) in dry tetrahydrofuran (0.5 mL) at 0 °C sodium hydride (60% dispersion in mineral oil, 3 mg, 0.068 mmol) was added. The mixture was stirred at 0 °C for 30 min. Then chloromethyl ethyl ether (10 µL, 0.108 mmol) was added. The ice–water bath was removed and the mixture was stirred at room temperature overnight. On the next day, the solvent was removed under reduced pressure. The residue was taken up in water (1 mL) and extracted with dichloromethane (3 × 5 mL). The organic phase was dried over magnesium sulfate and evaporated under reduced pressure. The residue was purified on a silica column by using a mixture of hexane/ethyl acetate 95:5 as eluent. Yield: 3.34 mg, 18%. ^{1}H NMR (500 MHz, DMSO-d_6), δ, ppm: 8.08 (dd, J = 7.9, 1.3 Hz, 1H, H_{Ar}), 7.54 – 7.50 (m, 2H, H_{Ar}), 7.48 – 7.42 (m, 2H, H_{Ar}), 7.24 – 7.22 (m, 1H, H_{Ar}), 7.11 (t, J = 7.1 Hz, 1H, H_{Ar}), 7.02 (t, J = 7.5 Hz, 1H, H_{Ar}), 6.28 (s, 1H, H_{Ar}), 5.48 (d, J = 10.5 Hz, 1H, CH_2OCH$_2$CH$_3$), 5.42 (d, J = 2.8 Hz, 1H, CH_2CO), 4.43 (d, J = 10.5 Hz, 1H, CH$_2$OCH_2CH$_3$), 3.67 – 3.62 (m, J = 1.8 Hz, 2H, CH_2OCH$_2$CH$_3$), 3.58 – 3.53 (m, 1H, CH$_2$OCH_2CH$_3$), 3.47 (d, J = 16.9 Hz, 1H, CH$_2$OCH_2CH$_3$), 3.37 (s, 1H, CH$_2$OCH_2CH$_3$), 3.24 – 3.22 (m, 1H,

$CH_2OCH_2CH_3$), 1.12 (t, *J* = 8.3, 5.7 Hz, 3H, $CH_2OCH_2CH_3$), 0.95 (t, *J* = 7.0 Hz, 3H, $CH_2OCH_2CH_3$).

*5,8-Bis(ethoxymethyl)-7,8-dihydro[2,3-d][1]benzazepin-6(5H)-one (**18**).* A mixture of *N*-(ethoxymethyl)-2-[1-(ethoxymethyl)-1*H*-indole-2-yl]-*N*-(2-iodophenyl)acetamide (**17**, 20 mg, 0.04 mmol), triphenylphosphine (5.3 mg, 0.02 mmol), palladium(II) acetate (2.25 mg, 0.01 mmol), and silver carbonate (27.5 mg, 0.10 mmol) was vigorously stirred in dry dimethylformamide (2 mL) under argon atmosphere at 100 °C for 1 h. Then the mixture was cooled to room temperature, the solvent was removed under reduced pressure, the residue was taken up in dichloromethane, and filtered over Celite®, followed by dichloromethane washing. The solvent was evaporated under reduced pressure, and the crude product was purified on a silica column by using a mixture of hexane/ethyl acetate 3:1 as eluent. Yield: 3 mg, 30%. ^{1}H NMR (500 MHz, $CDCl_3$), *δ*, ppm: 7.94 – 7.90 (m, 2H, H_{Ar}), 7.86 (dd, *J* = 7.8, 1.7 Hz, 1H, H_{Ar}), 7.56 (d, *J* = 8.1 Hz, 1H, H_{Ar}), 7.40 – 7.37 (m, 2H, H_{Ar}), 7.32 – 7.29 (m, 1H, H_{Ar}), 7.28 – 7.26 (m, *J* = 1.0 Hz, 1H, $H_{Ar,}$ (overlapped solvent residual peak)), 5.72 (d, *J* = 11.6 Hz, 1H, CH_2), 5.60 (d, *J* = 11.6 Hz, 1H, CH_2), 5.41 (d, *J* = 9.9 Hz, 1H, $CH_2OCH_2CH_3$), 4.70 (d, *J* = 9.9 Hz, 1H, $CH_2OCH_2CH_3$), 4.01 (d, *J* = 13.9 Hz, 1H, $CH_2OCH_2CH_3$), 3.68 – 3.63 (m, 1H, $CH_2OCH_2CH_3$), 3.54 – 3.46 (m, 2H, $CH_2OCH_2CH_3$), 3.38 (d, *J* = 13.9 Hz, 2H, $CH_2OCH_2CH_3$), 1.22 – 1.19 (m, 3H, $CH_2OCH_2CH_{3,}$ overlapped *n*-hexane peak)), 0.85 – 0.82 (m, 3H, $CH_2OCH_2CH_{3,}$ overlapped *n*-hexane peak). ESI-MS (acetonitrile/methanol + 1% water), positive: *m/z* 387.26 $[M + Na]^+$.

*Ethyl 1-benzyl-1H-indole-2-carboxylate (**19**).* A suspension of ethyl 1*H*-indole-2-carboxylate (6.00 g, 31.7 mmol) in dry acetonitrile was cooled to 0 °C and sodium hydride (60% dispersion in mineral oil, 1.77 g, 44.4 mmol) was added in small portions. The resulting mixture was stirred at 0 °C for 10 min and a solution of benzyl bromide (4.52 mL, 38.0 mmol) in dry acetonitrile (50 mL) was added dropwise. Afterwards, the reaction mixture was allowed to slowly reach room temperature and stirred overnight. On the next day, the reaction was quenched with saturated aqueous ammonium chloride solution (100 mL) and extracted with dichloromethane (3 × 150 mL). The combined organic layers

were dried over magnesium sulfate and evaporated. The residue was purified on a silica column by using a mixture of hexane/ethyl acetate 9:1 as eluent. Yield: 7.80 g, 88%. ^{1}H NMR (500 MHz, DMSO-d_6), δ, ppm: 7.72 (d, J = 8.0 Hz, 1H), 7.57 (d, J = 8.5 Hz, 1H), 7.38 (d, J = 0.4 Hz, 1H), 7.33 – 7.29 (m, 1H, H_{Ar}), 7.26 (t, J = 7.4 Hz, 2H, H_{Ar}), 7.19 (t, J = 7.3 Hz, 1H, H_{Ar}), 7.14 (t, J = 7.4 Hz, 1H, H_{Ar}), 7.02 (d, J = 7.2 Hz, 2H, H_{Ar}), 5.87 (s, 2H, $CH_2C_6H_5$), 4.31 – 4.25 (m, 2H, CH_2CH_3), 1.28 (t, J = 7.1 Hz, 3H, CH_2CH_3). Preparation of compound **19** by slightly different methods has been described in the literature; however, NMR data in DMSO-d_6 was not reported.[8],[16],[17],[18],[19],[20]

*1-Benzyl-1H-indole-2-carboxylic acid (**20**).* A suspension of ethyl 1-benzyl-1*H*-indole-2-carboxylate (**19**, 3.00 g, 10.75 mmol) and lithium hydroxide monohydrate in tetrahydrofuran/methanol/water 1:1:1 (54 mL) was stirred at room temperature overnight. On the next day, water (150 mL) was added and the resulting mixture was acidified with 1 M hydrochloric acid to pH ≈ 2. The resulting mixture was extracted with ethyl acetate (2 × 200 mL). The combined organic layers were dried over magnesium sulfate and evaporated. The raw product was recrystallized from ethyl acetate (20 mL). Yield: 1.45 g, 54%. ^{1}H NMR (500 MHz, DMSO-d_6), δ, ppm: 13.00 (s, 1H, COOH), 7.70 (d, J = 8.0 Hz, 1H, H_{Ar}), 7.53 (d, J = 8.5 Hz, 1H, H_{Ar}), 7.33 (s, 1H, H_{Ar}), 7.30 – 7.23 (m, 3H, H_{Ar}), 7.19 (t, J = 7.3 Hz, 1H, H_{Ar}), 7.12 (t, J = 7.4 Hz, 1H, H_{Ar}), 7.02 (d, J = 7.2 Hz, 2H, H_{Ar}), 5.88 (s, 2H, $CH_2C_6H_5$). X-ray diffraction quality crystals were obtained by slow evaporation of a solution of **20** in ethyl acetate. Preparation of compound **20** by slightly different methods was described in the literature; however, NMR data was reported in a mixture of DMSO-d_6 and $CDCl_3$.[21]

*1-Benzyl-1H-indole-2-carboxylic anhydride (**21**).* (From an attempt to synthesize 1-(1-benzyl-1*H*-indol-2-yl)-2-diazoethan-1-one). A few drops of dry dimethylformamide were added to a suspension of 1-benzyl-1*H*-indole-2-carboxylic acid (500 mg, 1.99 mmol) in dry dichloromethane (5 mL). The mixture was cooled to 0 °C and a solution of oxalyl chloride (256 µL, 2.99 mmol) in dry dichloromethane (5 mL) was added dropwise. The resulting mixture was stirred at room temperature and the reaction was monitored by TLC. After 3 h complete conversion was observed, and the solvent was removed under

reduced pressure. Trimethylsilyl diazomethane (2 M in hexane, 2.08 mL, 4.16 mmol) was diluted with dry acetonitrile and triethylamine (303 µL, 2.19 mmol) was added. The solution was cooled to 0 °C and a solution of the acid chloride in dry acetonitrile (5 mL) was added dropwise. The resulting mixture was allowed to slowly reach room temperature and stirred overnight. On the next day, the solvent was removed under reduced pressure and the residue was taken up in saturated aqueous sodium bicarbonate solution (30 mL). The resulting mixture was extracted with dichloromethane (3 × 30 mL). The combined organic layers were dried over magnesium sulfate and evaporated. The residue was purified on a silica column by using a mixture of hexane/ethyl acetate 9:1 as eluent. Yield: 166 mg, 34%. ^{1}H NMR (500 MHz, DMSO-d_6), δ, ppm: 7.81 (d, J = 8.1 Hz, 2H, H_{Ar}), 7.74 (s, 2H, H_{Ar}), 7.68 (d, J = 8.6 Hz, 2H, H_{Ar}), 7.43 (t, J = 7.4 Hz, 2H, H_{Ar}), 7.28 (t, J = 7.4 Hz, 4H, H_{Ar}), 7.25 – 7.19 (m, 4H, H_{Ar}), 7.06 (d, J = 7.3 Hz, 4H, H_{Ar}), 5.88 (s, 4H, $CH_2C_6H_5$). X-ray diffraction quality crystals of **21** were obtained by recrystallization from ethanol. Compound **21** has been reported previously; however, we were unable to retrieve any information regarding its preparation or spectral data from the patent in Japanese language.[22]

*1,4-bis(2-Bromophenyl)piperazine-2,5-dione (**22**).* (From an attempt to synthesize *N*-(2-bromophenyl)-2-(1*H*-indol-2-yl)acetamide, by a known protocol) [23]. A suspension of 2-bromo-*N*-(2-bromophenyl)acetamido [3] (586 mg, 2,00 mmol), 1*H*-indole (117 mg, 1.00 mmol), norbornene (188 mg, 2.00 mmol), potassium carbonate (276 mg, 2.00 mmol), and bis(acetonitrile)palladium(II) dichloride (26 mg, 0.10 mmol) in 0.5 M water/dimethylacetamide (5 mL) was degassed 3 times by the freeze-pump-thaw method and stirred overnight at 70 °C. After cooling to room temperature, the reaction mixture was diluted with diethyl ether and filtered. The filtrate was evaporated and the residue was crystallized from ethyl acetate to give **22** as a beige solid. Yield: 259 mg, 61%. ^{1}H NMR (500 MHz, DMSO-d_6), δ, ppm: 7.79 (d, J = 7.9 Hz, 2H, H_{Ar}), 7.53 (dd, J = 5.8, 3.6 Hz, 4H, H_{Ar}), 7.41 – 7.35 (m, 2H, H_{Ar}), 4.70 – 4.04 (m, 2H, CH_2). ESI-MS (acetonitrile/methanol + 1% water), positive: *m/z* 424.92 $[M + H]^+$, 446.89 $[M + Na]^+$. X-ray diffraction quality crystals were obtained by slow evaporation of a solution of **22** in ethyl acetate.

*Ethyl 3-iodo-1H-indole-2-carboxylate (**23**)* [24]. To indole-2-carboxylate (1.0 g, 5.29 mmol) in dry dimethylformamide (20 mL) powdered potassium hydroxide (0.89 g, 15.86 mmol) and iodine (1.48 g, 5.53 mmol) in dry dimethylformamide (1 mL) were added. Then, the reaction mixture was stirred at room temperature for 3 h. After completion of the reaction as indicated by TLC, the reaction mixture was quenched with 10% sodium bisulfite solution and extracted with ethyl acetate (3 × 50 mL). The ethyl acetate extract was repeatedly washed with 10% sodium thiosulfate and water. The combined organic phases were dried and concentrated under reduced pressure to afford the pure product. Yield: 1.33 g, 80%. ^{1}H NMR (500 MHz, $CDCl_3$), δ, ppm: 9.30 (s, 1H, N*H*), 7.57 (dd, J = 8.1, 0.8 Hz, 1H, H_{Ar}), 7.39 (d, J = 0.6 Hz, 2H, H_{Ar}), 7.23 (ddd, J = 8.0, 6.1, 1.8 Hz, 1H, H_{Ar}), 1.65 (s, 2H, CH_2CH_3), 1.47 (t, J = 7.1 Hz, 3H, CH_2CH_3). Preparation of compound **23** by a different method was described in the literature; NMR data was reported in $CDCl_3$.[25]

*2,2'-Dinitrobiphenyl (**24**).* (In an attempt to synthesize ethyl 3-(2-nitrophenyl)-1*H*-indole-2-carboxylate) [26]. A mixture of ethyl 3-iodo-1*H*-indole-2-carboxylate (**23**, 1.33 g, 4.22 mmol) and *o*-bromo-nitrobenzene (0.85 g, 4.22 mmol) in dimethyl sulfoxide (25 mL) was treated with tris(dibenzylideneacetone)dipalladium(0) (125.9 mg, 0.138 mmol) and copper powder (1.33 g, 20.76 mmol, 0.01 g-atom). The resulting mixture was vigorously stirred at 80 °C for 2 h and then cooled to room temperature, diluted with diethyl ether (80 mL), and filtered over Celite®. The filtrate was washed with water and brine, dried over magnesium sulfate, and evaporated under reduced pressure. The residue was purified on a silica column by using hexane/ethyl acetate 4:1 as eluent. Yield: 391 mg, 76%. ^{1}H NMR (500 MHz, DMSO-d_6), δ, ppm: 8.23 (dd, J = 7.9, 1.2 Hz, 2H, H_{Ar}), 7.84 (dd, J = 8.1, 1.5 Hz, 2H, H_{Ar}), 7.73 (m, 2H, H_{Ar}), 7.50 (td, J = 7.7, 1.5 Hz, 2H, H_{Ar}). X-ray diffraction quality crystals were grown by slow evaporation of a solution of **24** in ethyl acetate. Preparation of **24** by slightly different methods was described in the literature; NMR data was reported in $CDCl_3$.[27] The X-ray diffraction crystal structure of **24** at room temperature was reported previously.[28]

X-ray crystallography data

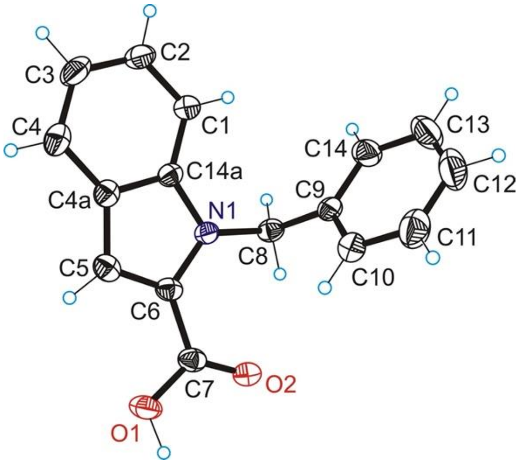

Figure S2: ORTEP view of **14** with thermal ellipsoids drawn at 50% probability level.

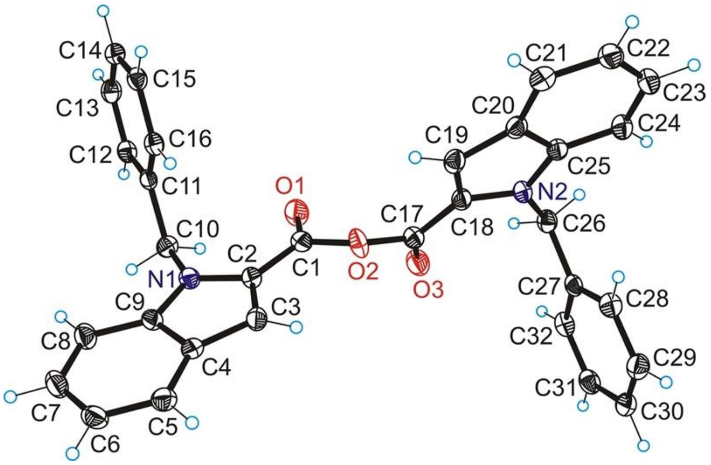

Figure S3: ORTEP view of **21** with thermal ellipsoids drawn at 50% probability level.

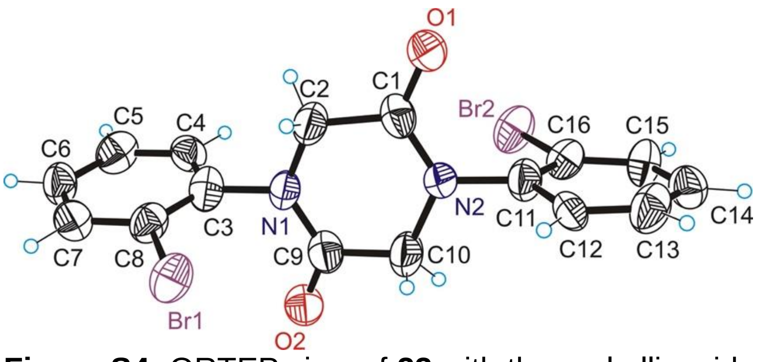

Figure S4: ORTEP view of **22** with thermal ellipsoids drawn at 50% probability level.

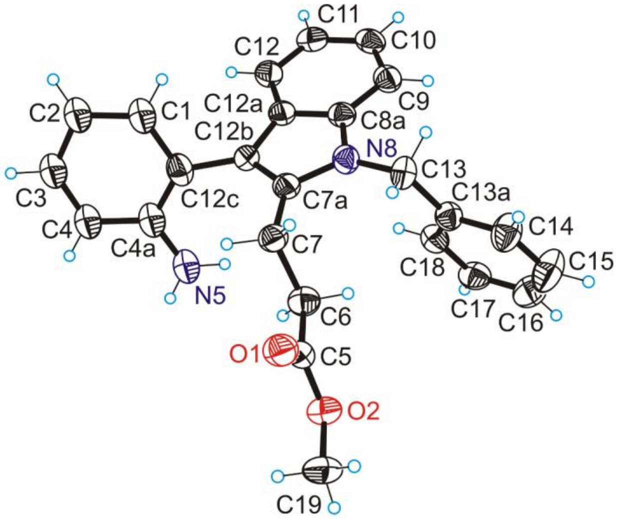

Figure S5: ORTEP view of **2c** with thermal ellipsoids drawn at 50% probability level.

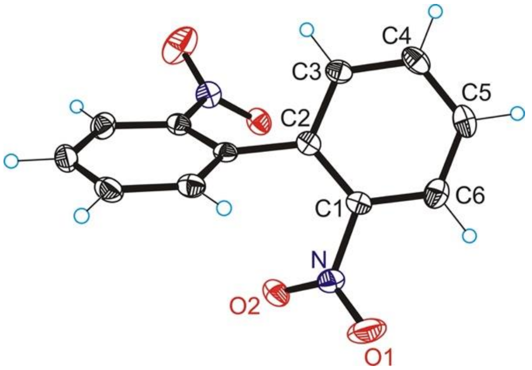

Figure S6: ORTEP view of **24** with thermal ellipsoids drawn at 50% probability level. This X-ray diffraction structure was determined at 100 K. The same structure with diffraction data collected at room temperature is available in Cambridge Structural Database with reference code ZZZJPA.

NMR-spectra

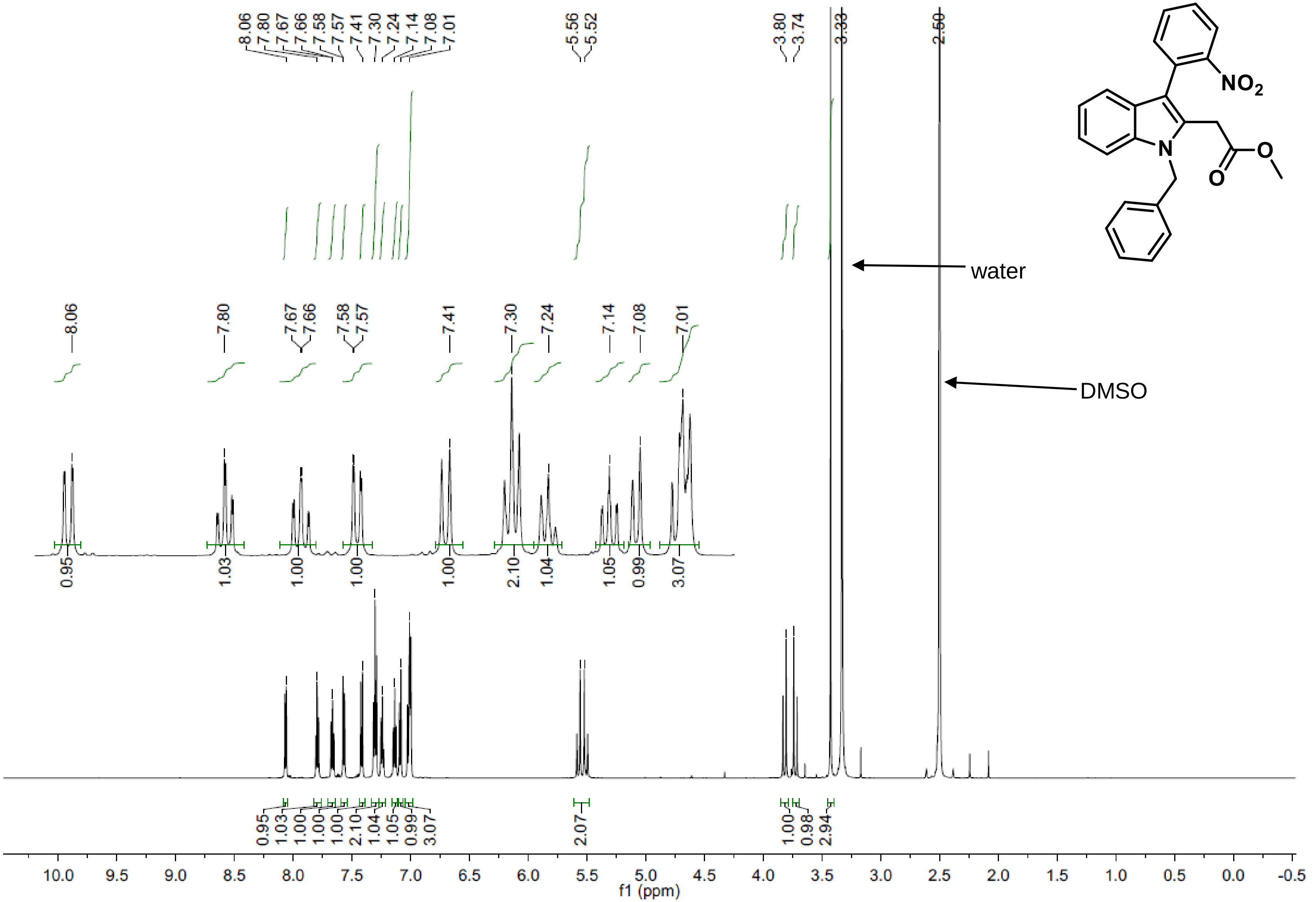

Figure S7: ^{1}H NMR spectrum of **1a** in DMSO (600 MHz).

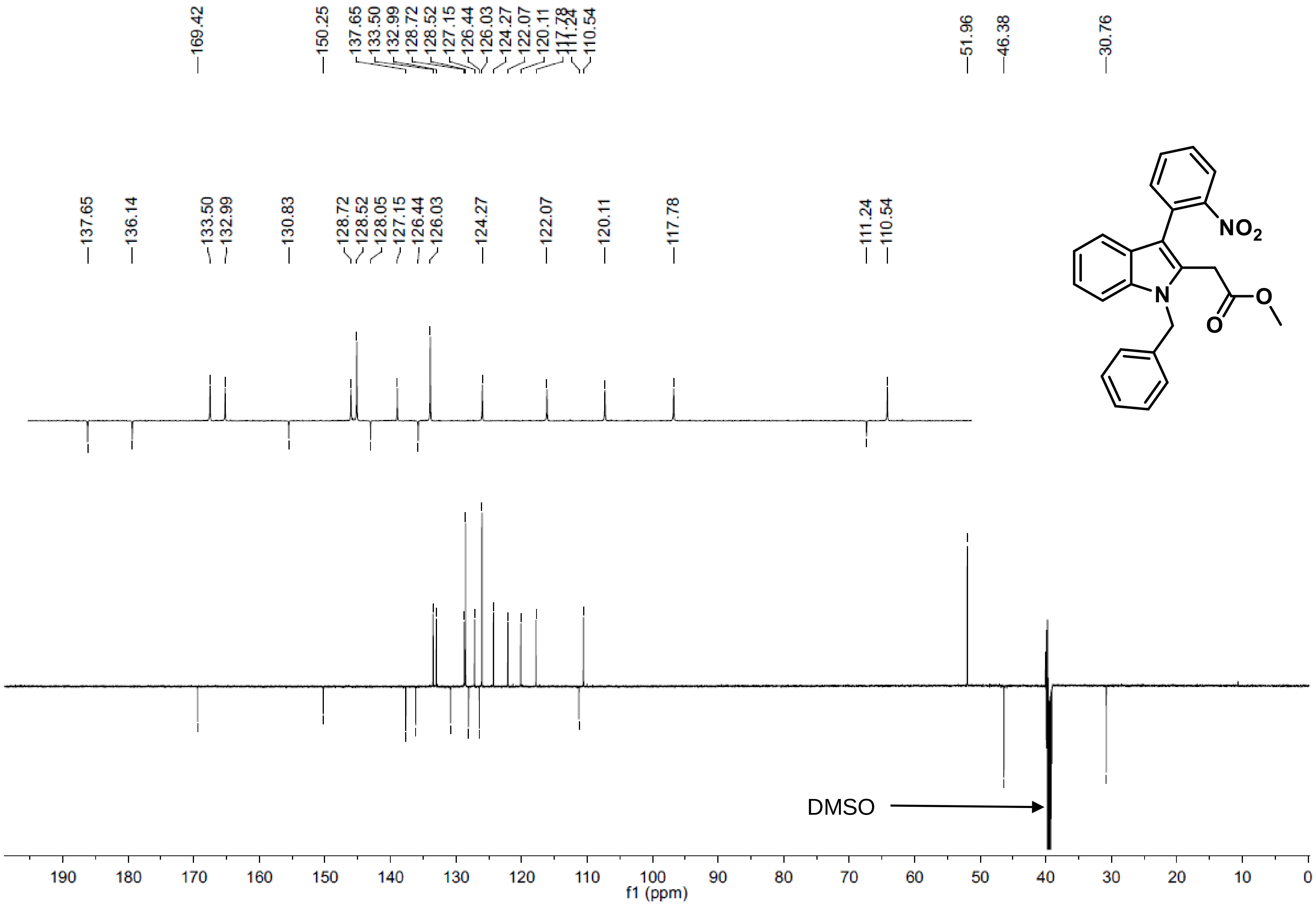

Figure S8: ^{13}C NMR spectrum of **1a** in DMSO (151 MHz).

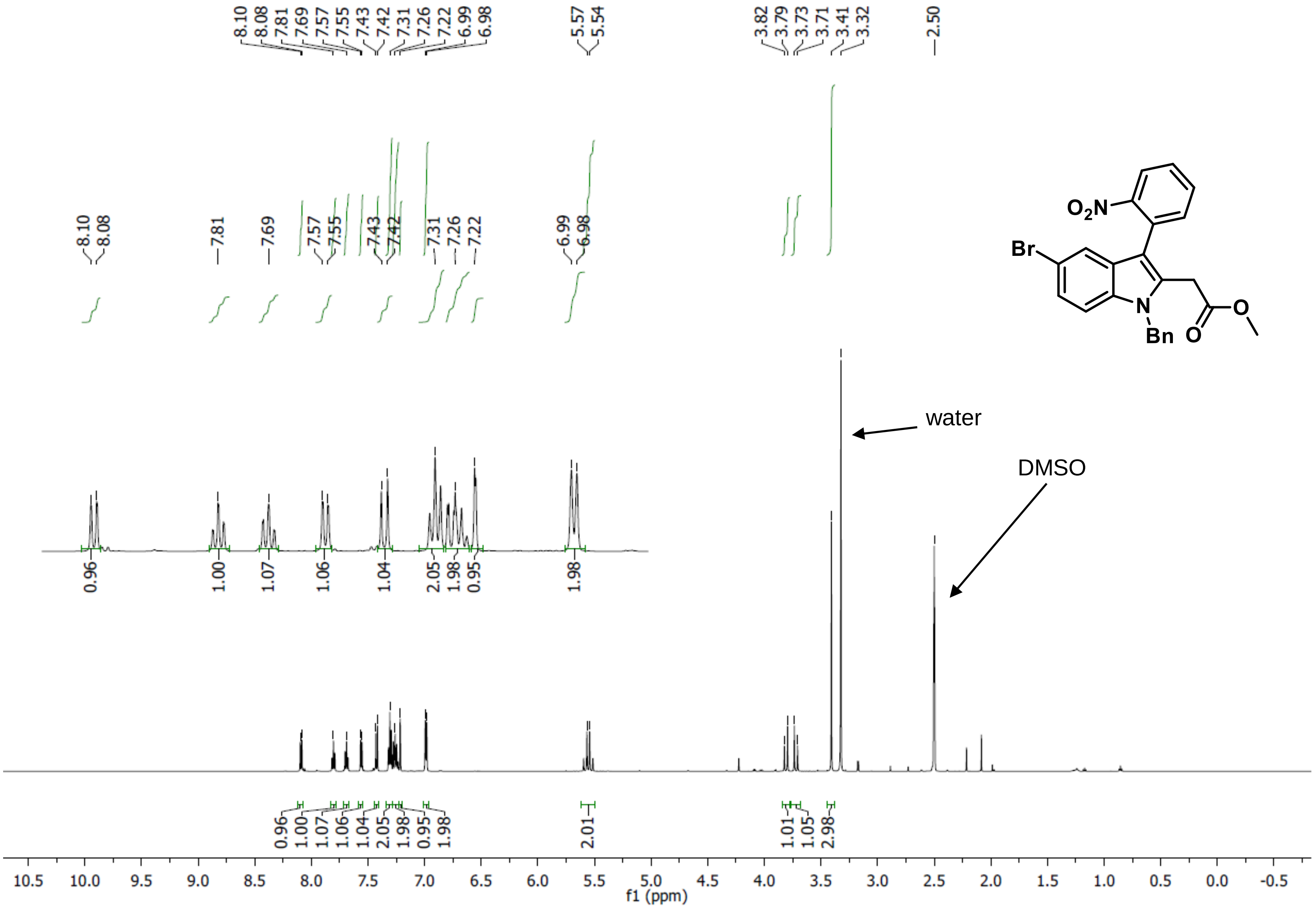

Figure S9: ^{1}H NMR spectrum of **1b** in DMSO (500 MHz).

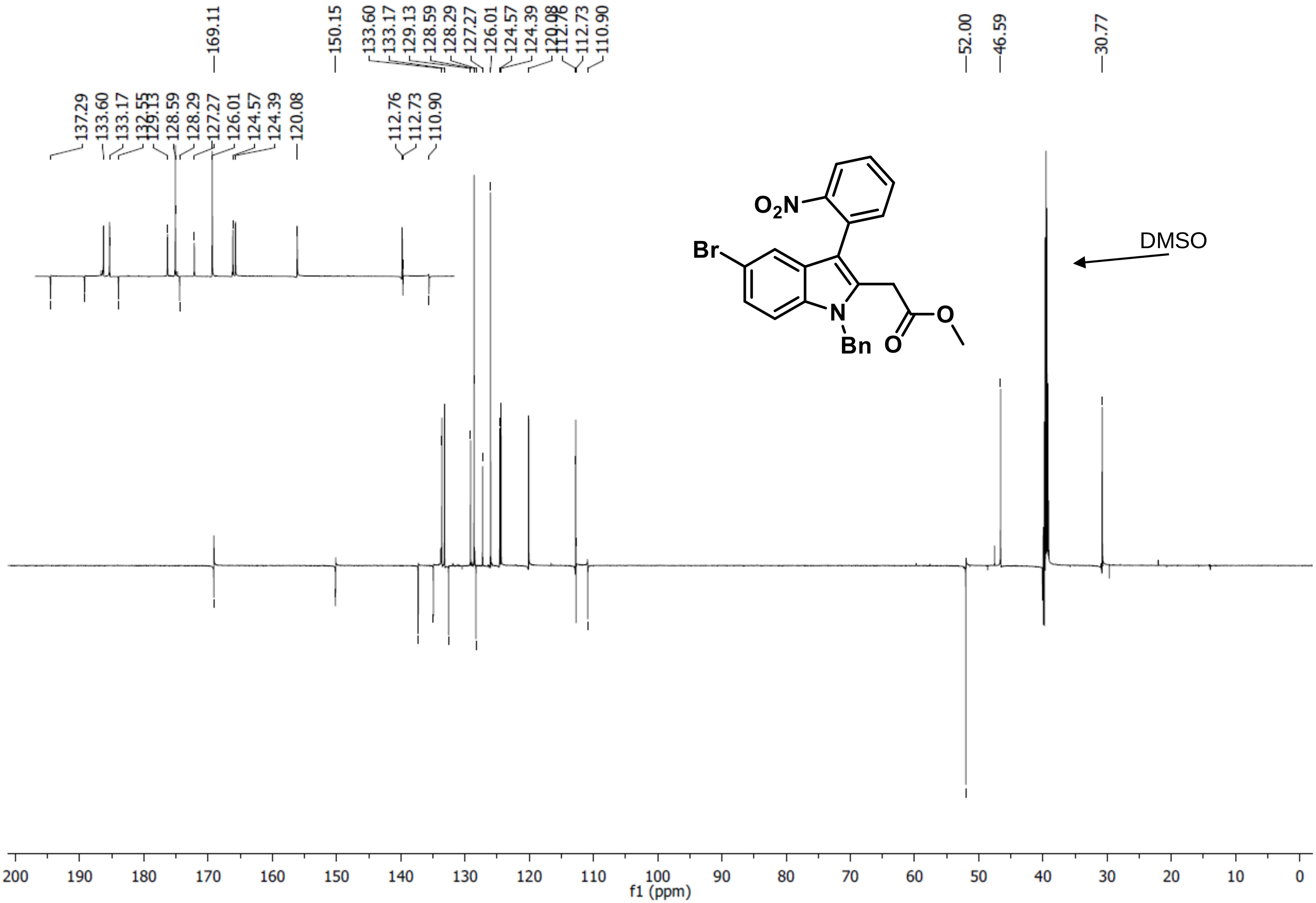

Figure S10: ^{13}C NMR spectrum of **1b** in DMSO (126 MHz).

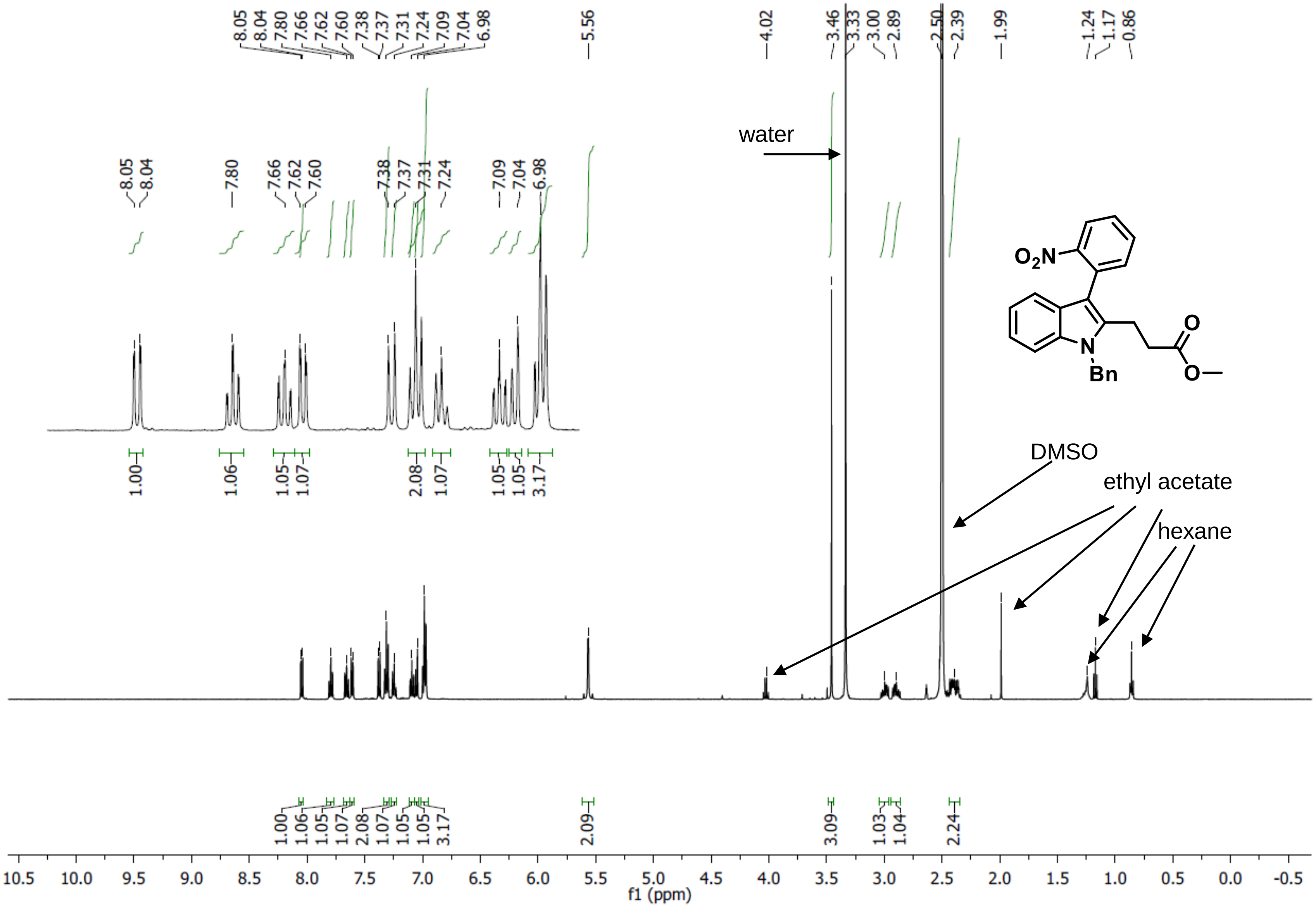

Figure S11: ^{1}H NMR spectrum of **1c** in DMSO (500 MHz).

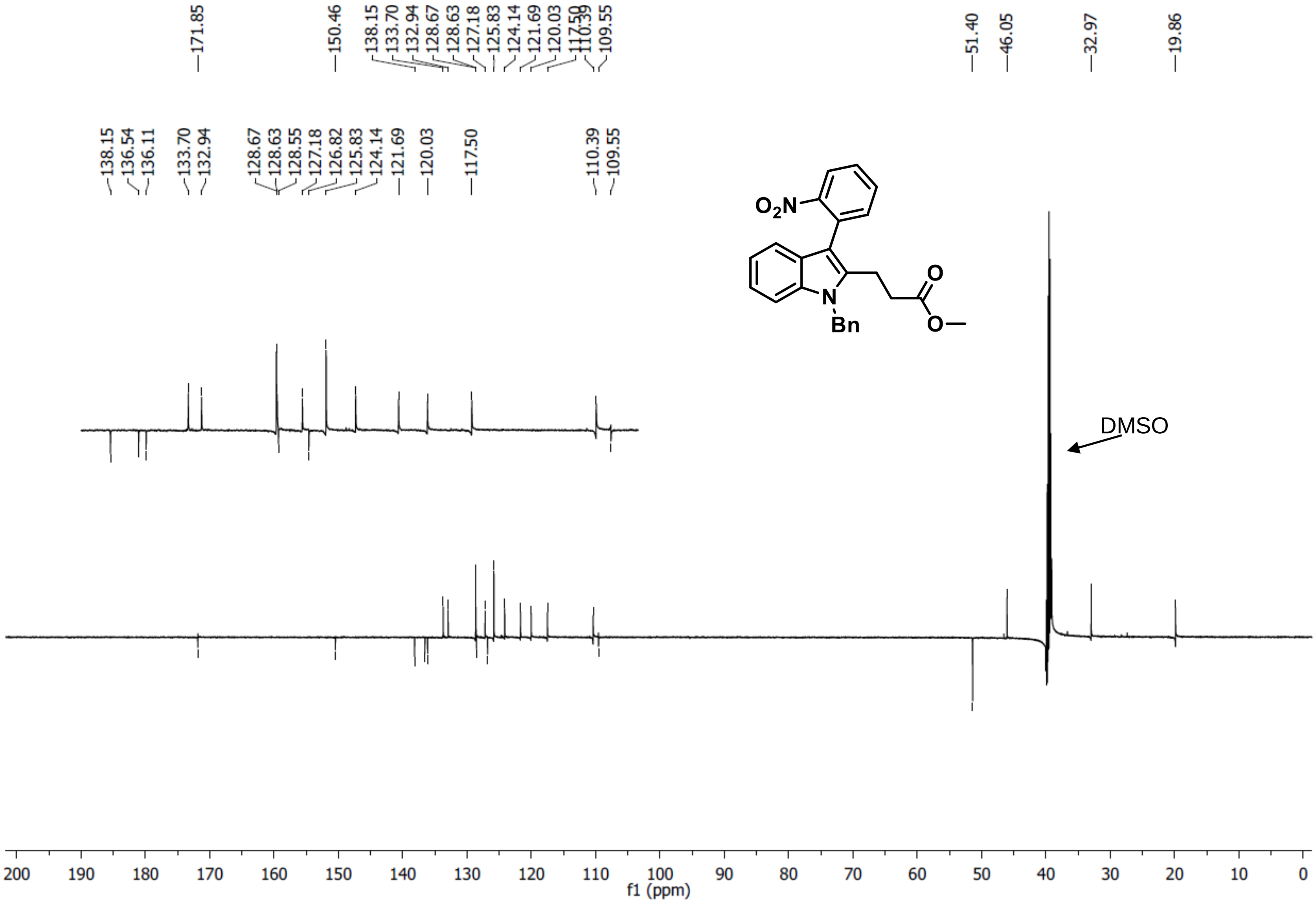

Figure S12: ^{13}C NMR spectrum of **1c** in DMSO (126 MHz).

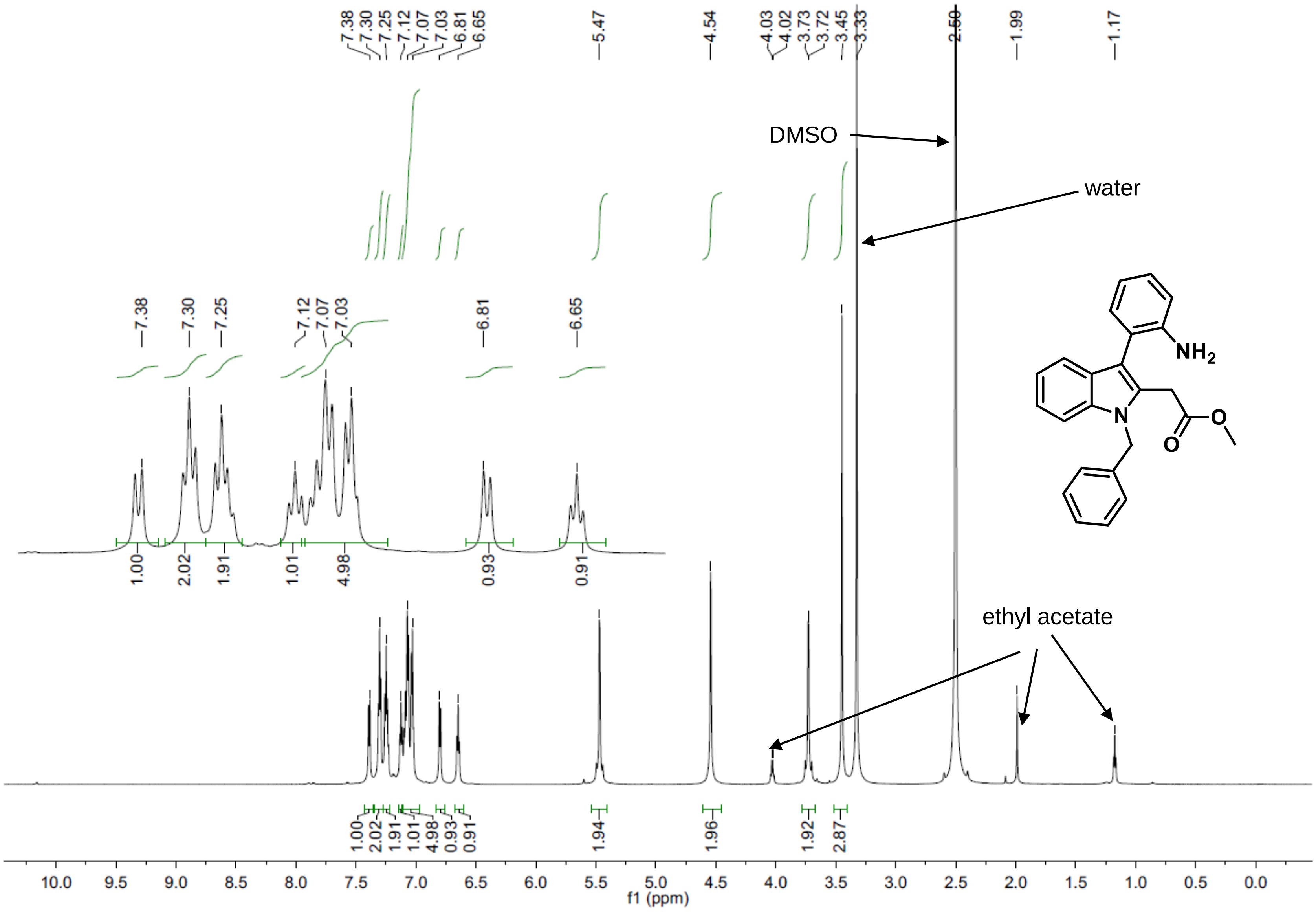

Figure S13: ^{1}H NMR spectrum of **2a** in DMSO (700 MHz).

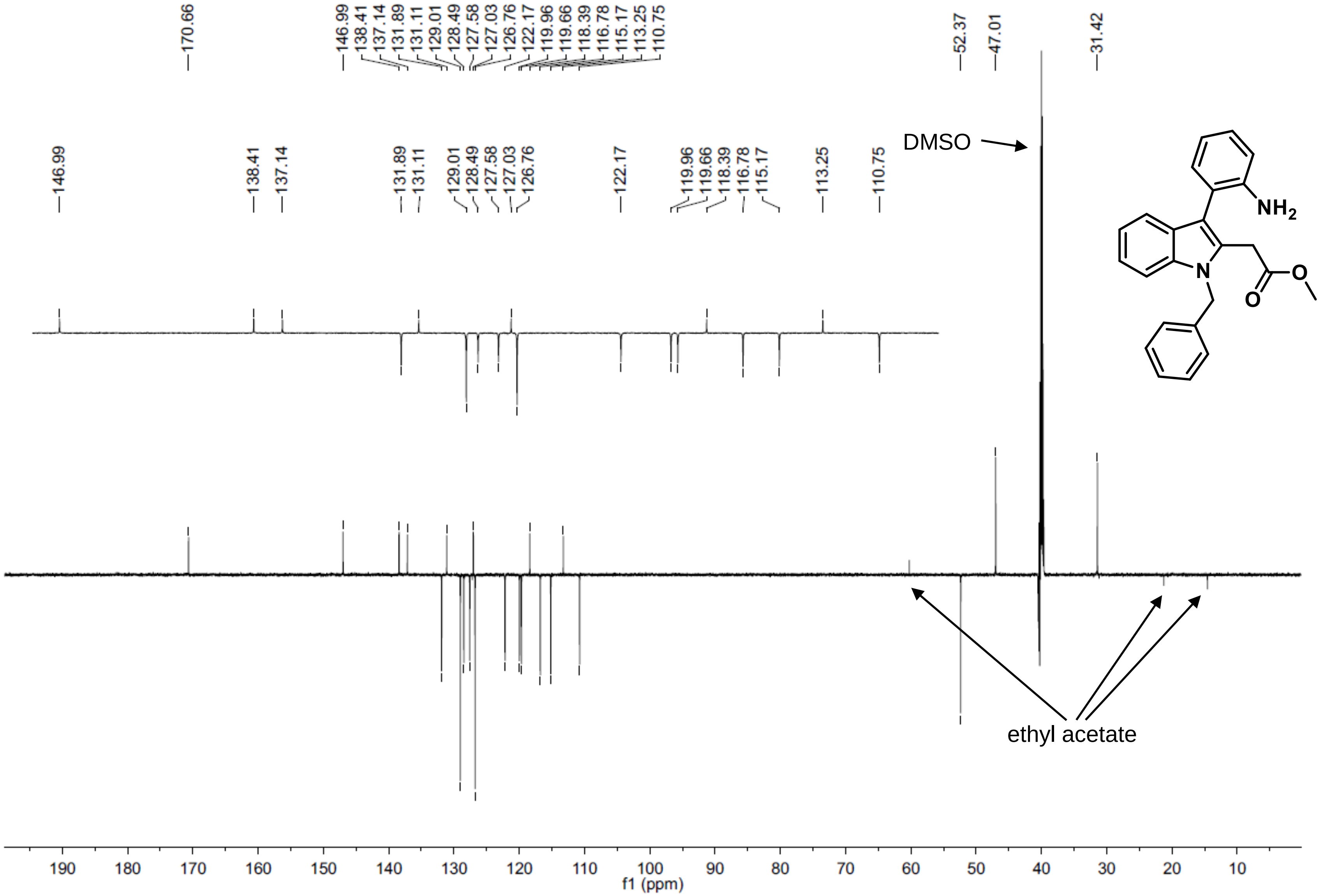

Figure S14: ^{13}C NMR spectrum of **2a** in DMSO (176 MHz).

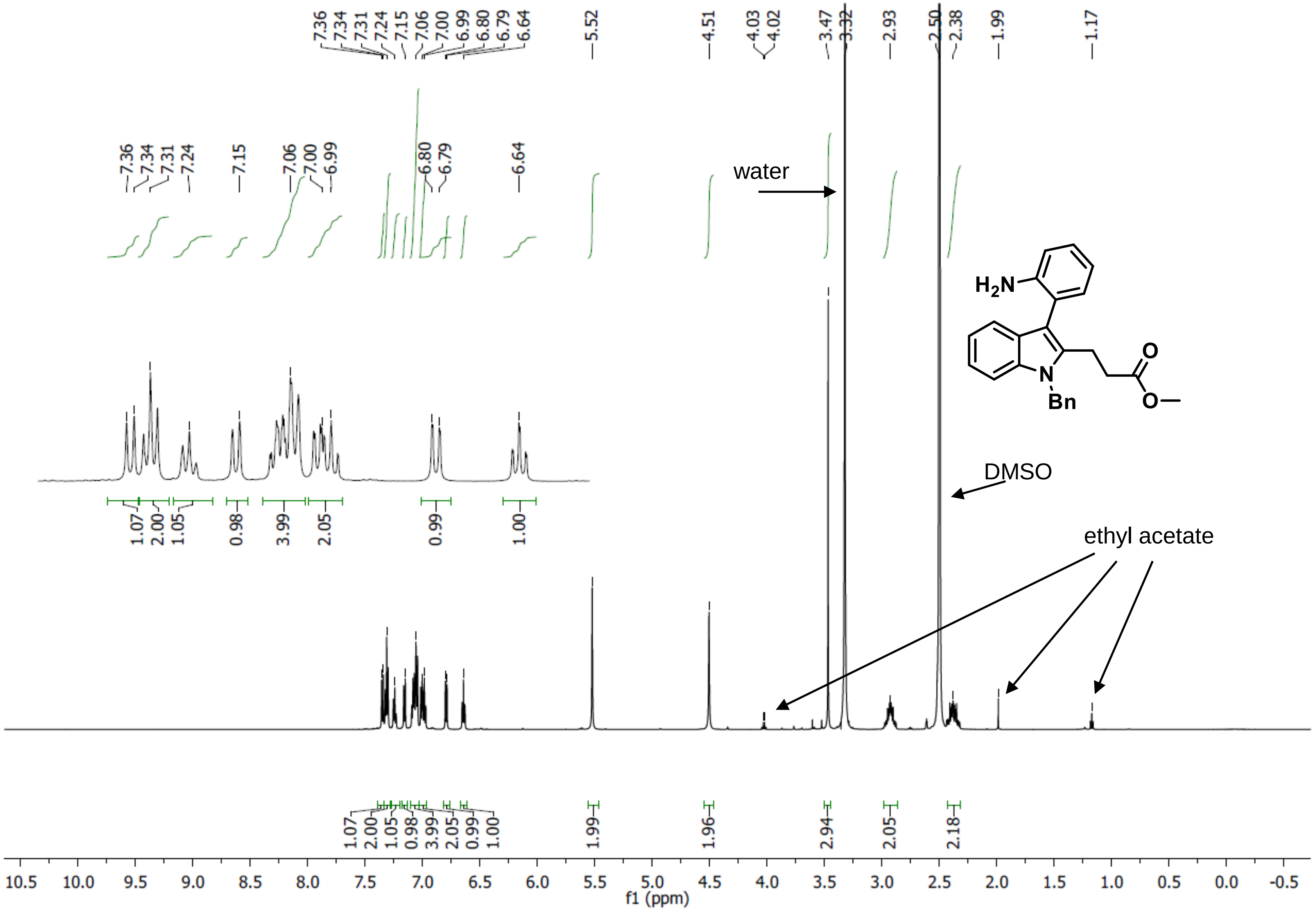

Figure S15: ^{1}H NMR spectrum of **2c** in DMSO (500 MHz).

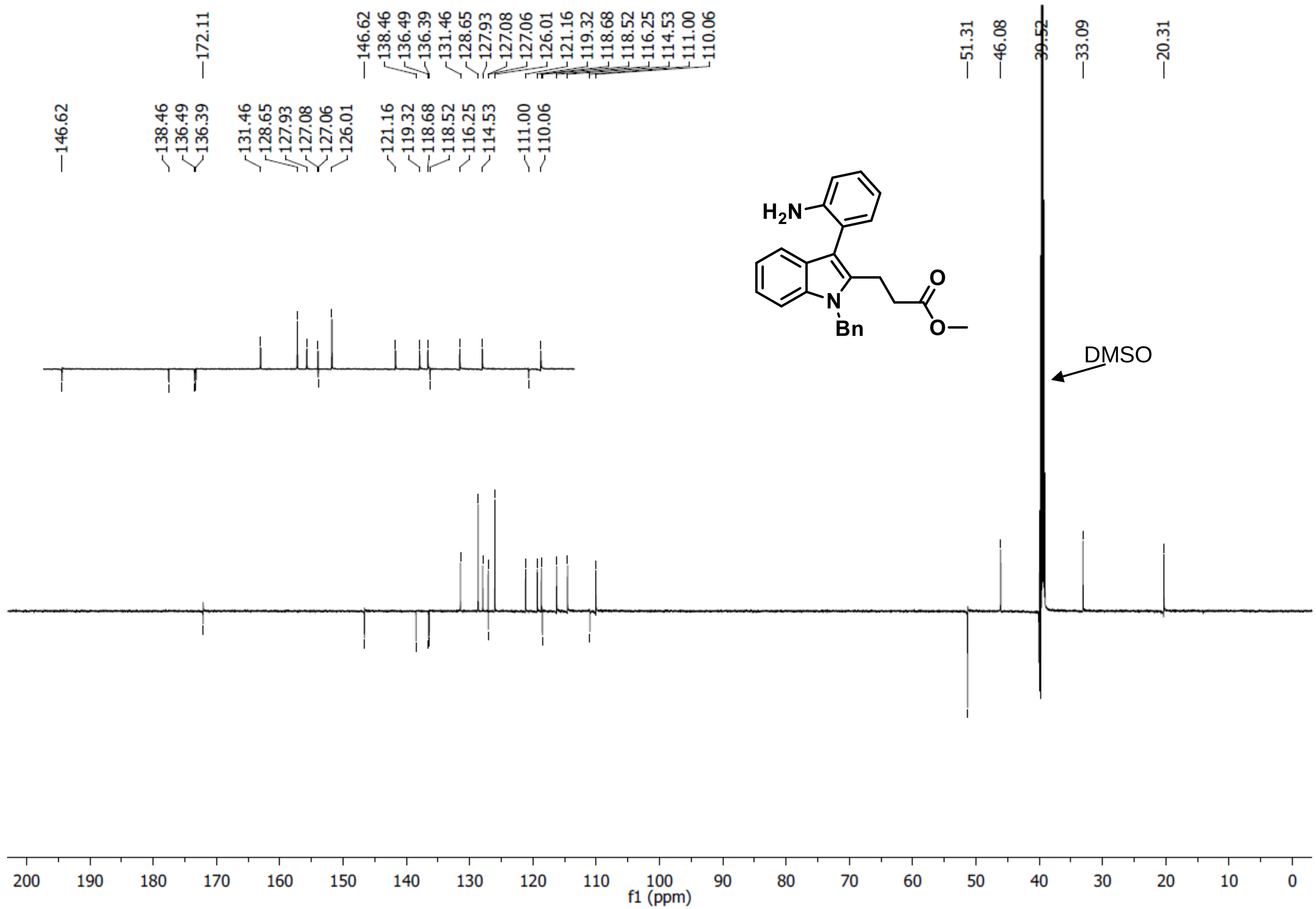

Figure S16: ^{13}C NMR spectrum of **2c** in DMSO (126 MHz).

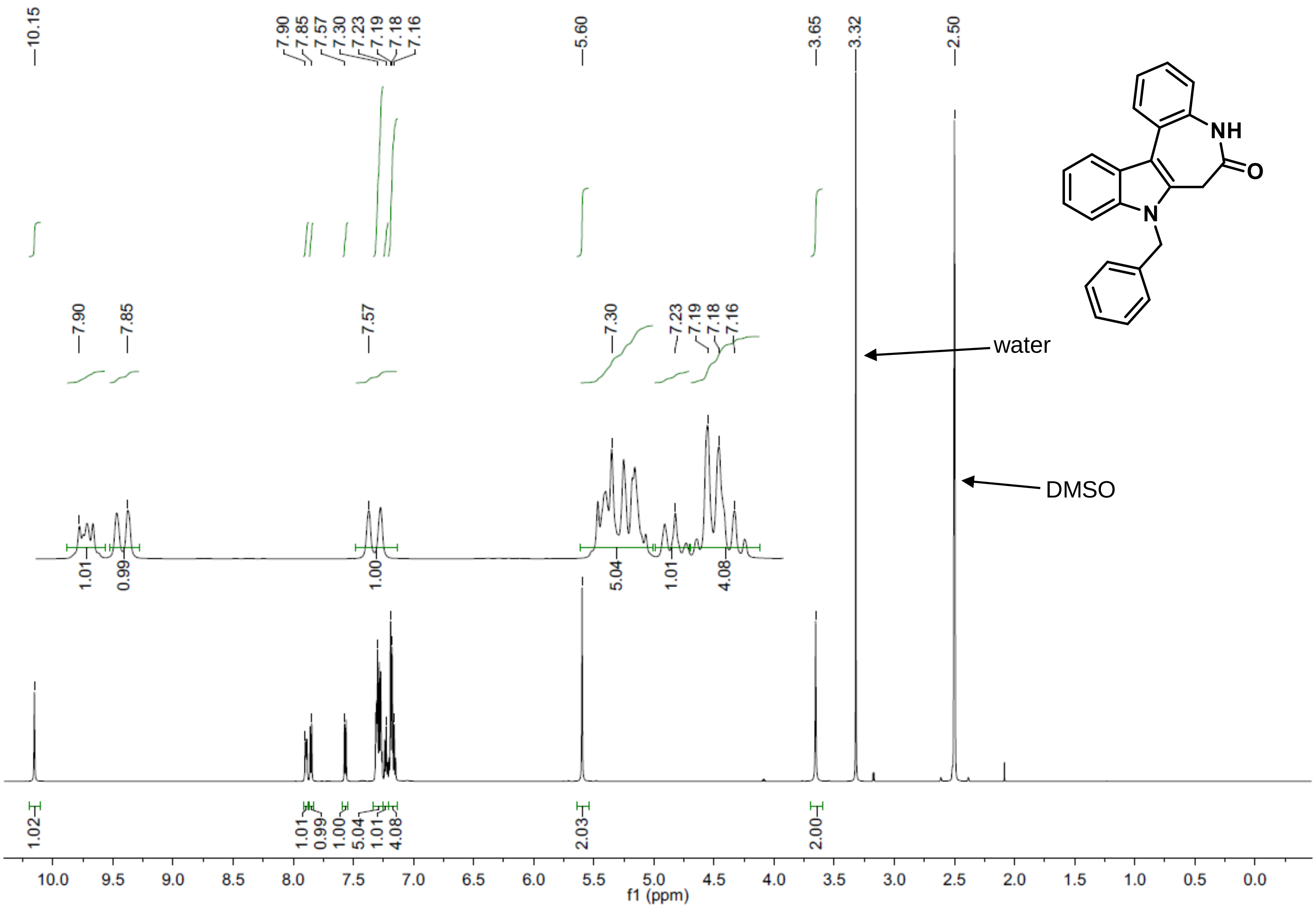

Figure S17. ^{1}H NMR spectrum of **3a** in DMSO (600 MHz).

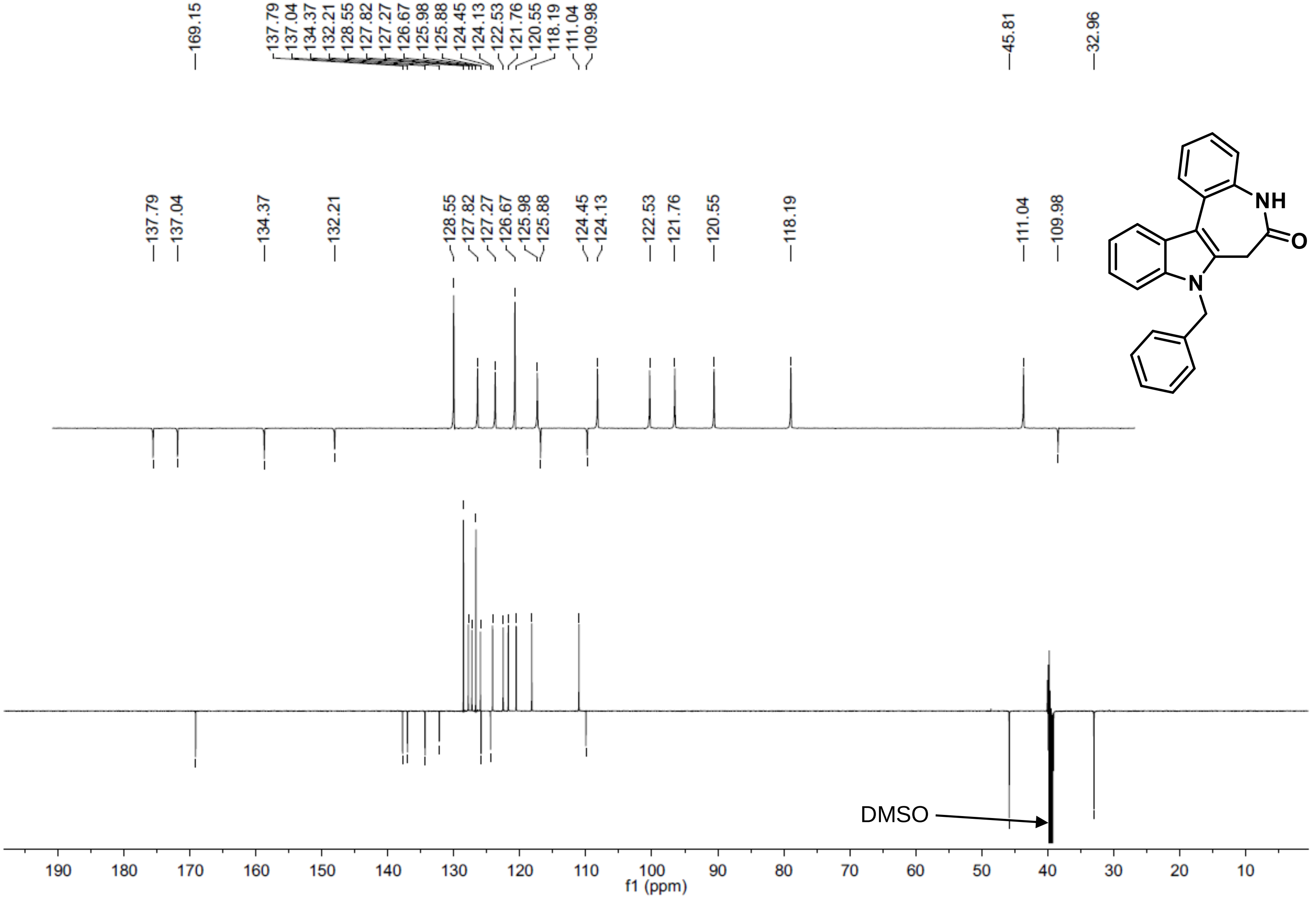

Figure S18: ^{13}C NMR spectrum of **3a** in DMSO (151 MHz).

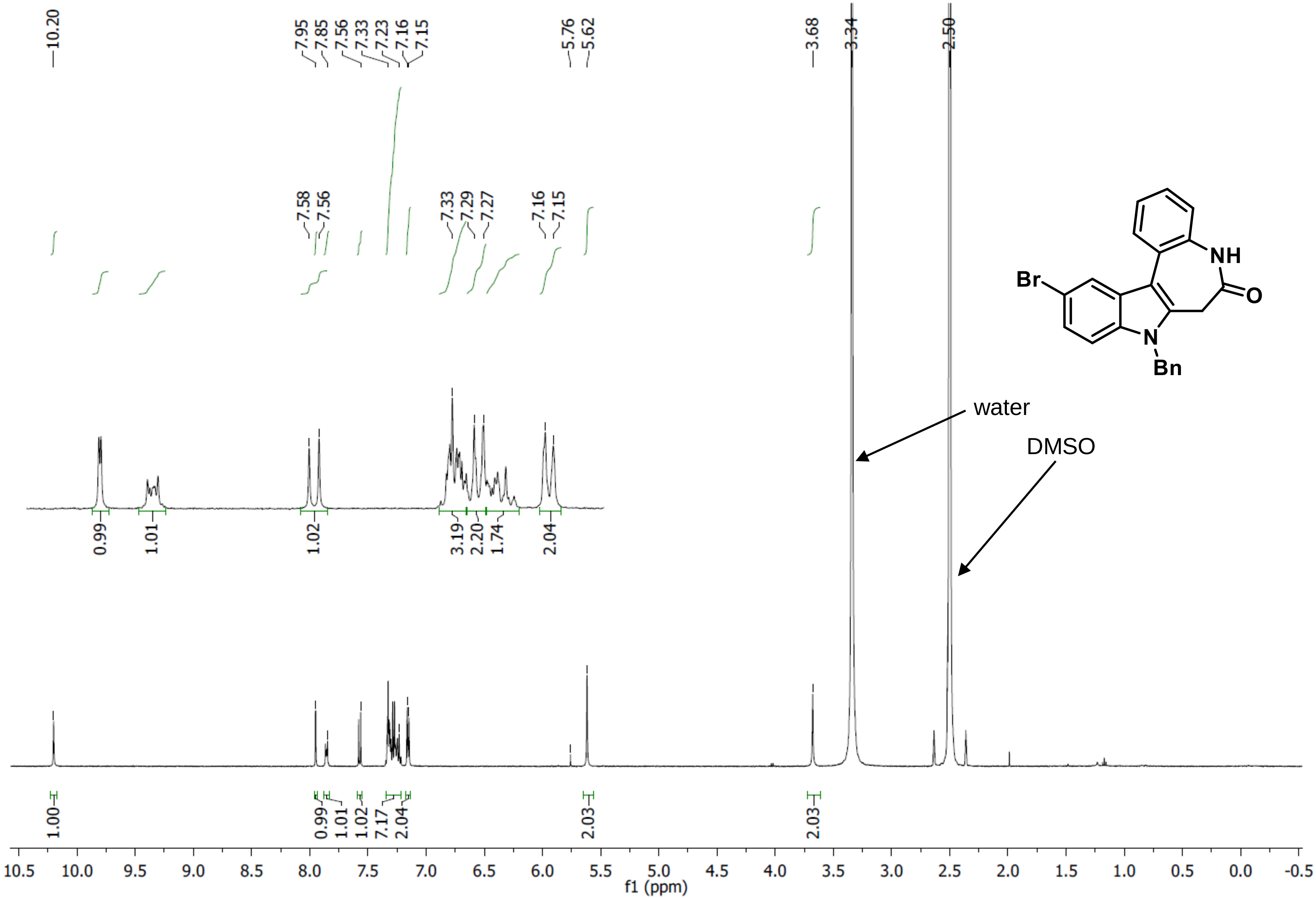

Figure S19. ^{1}H NMR spectrum of **3b** in DMSO (500 MHz).

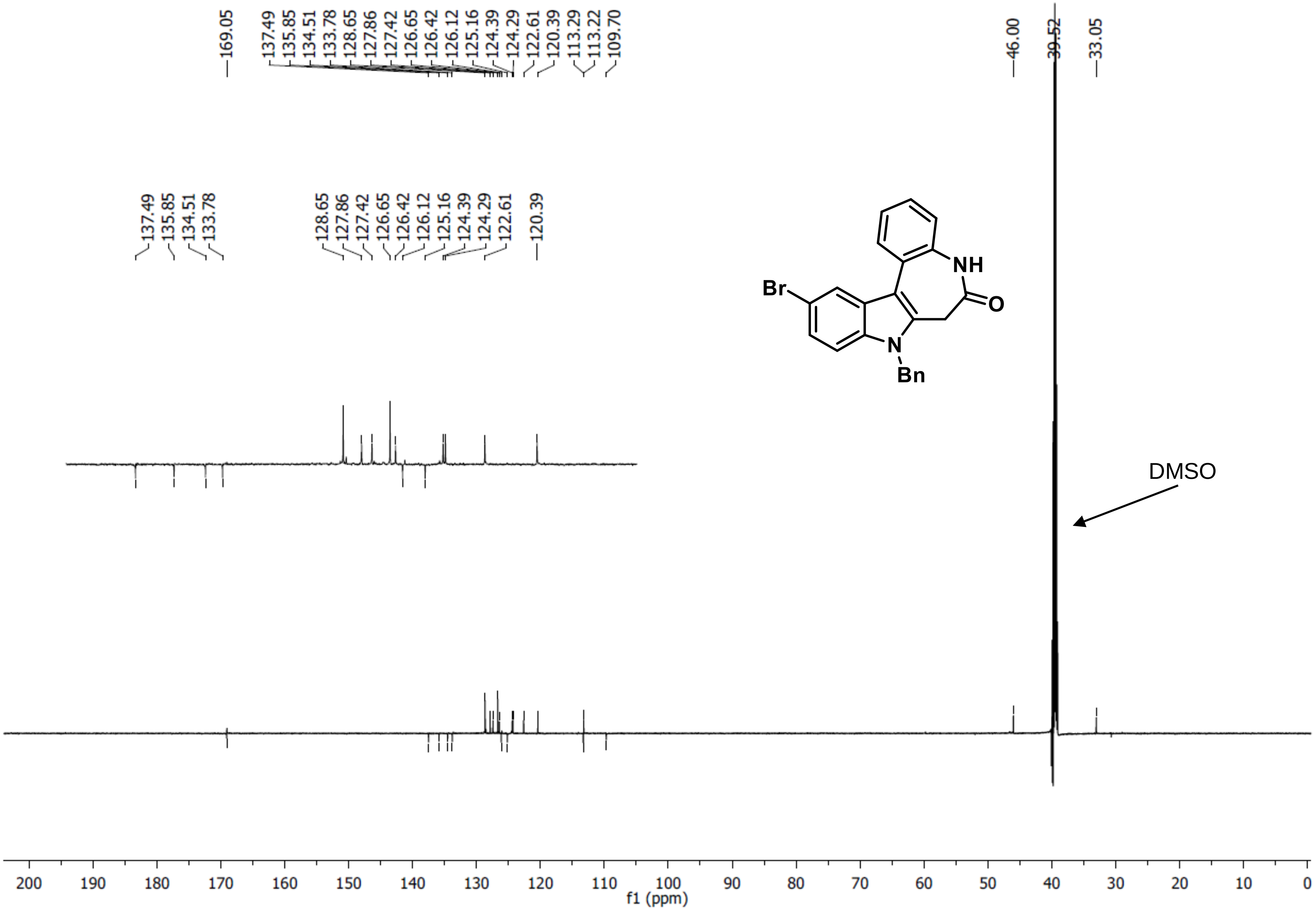

Figure S20: ^{13}C NMR spectrum of **3b** in DMSO (126 MHz).

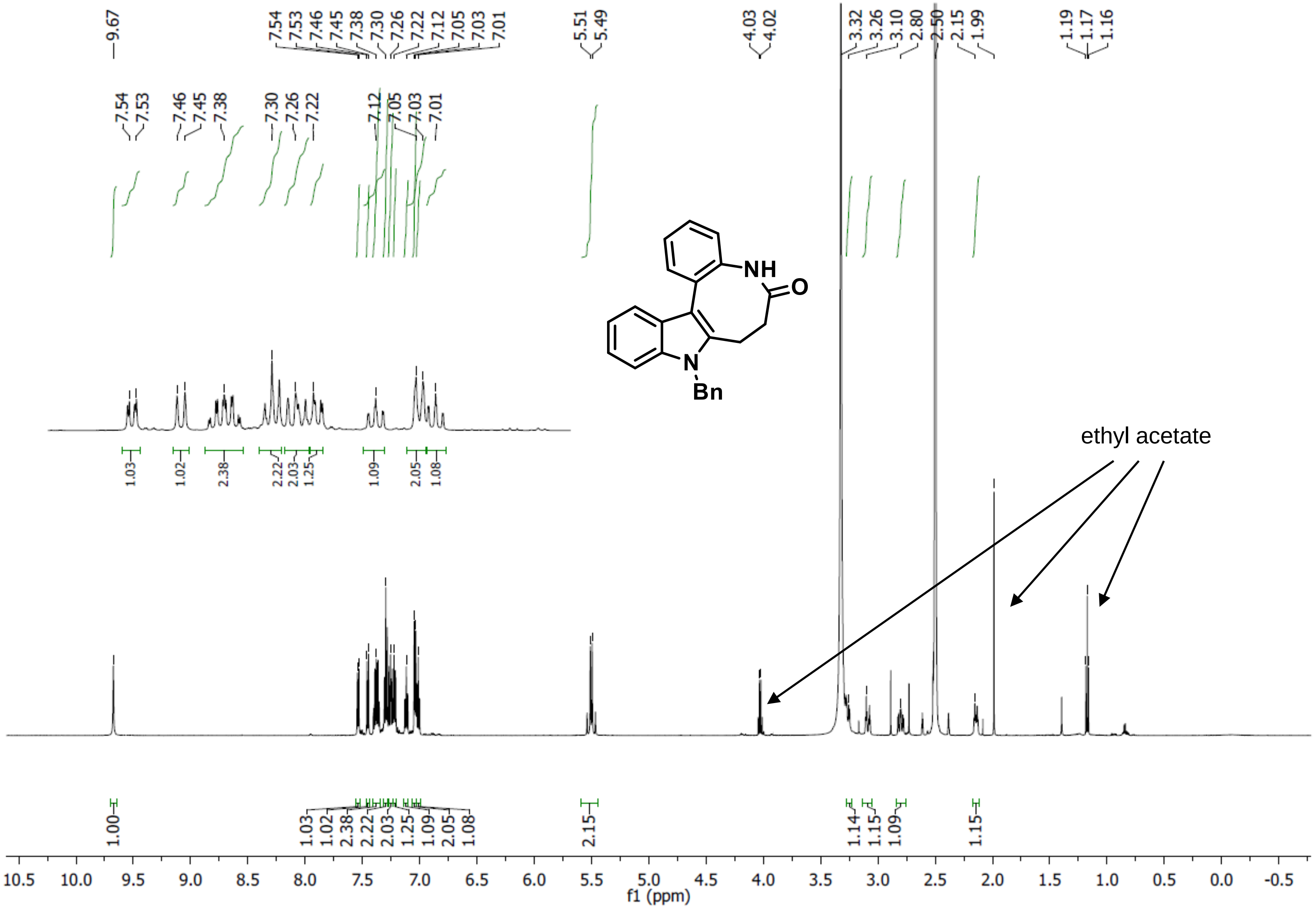

Figure S21. ^{1}H NMR spectrum of **3c** in DMSO (500 MHz).

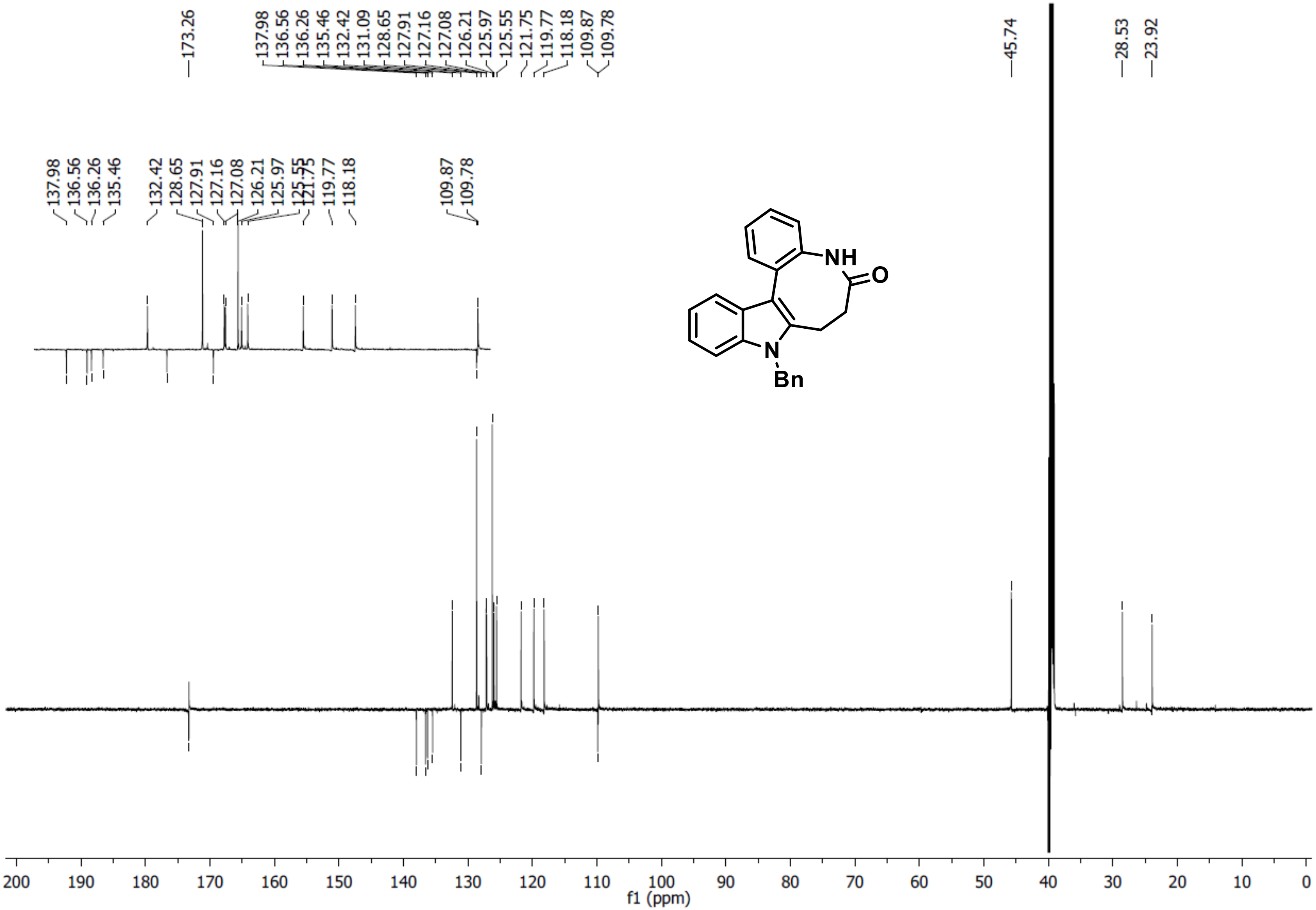

Figure S22: ^{13}C NMR spectrum of **3c** in DMSO (126 MHz).

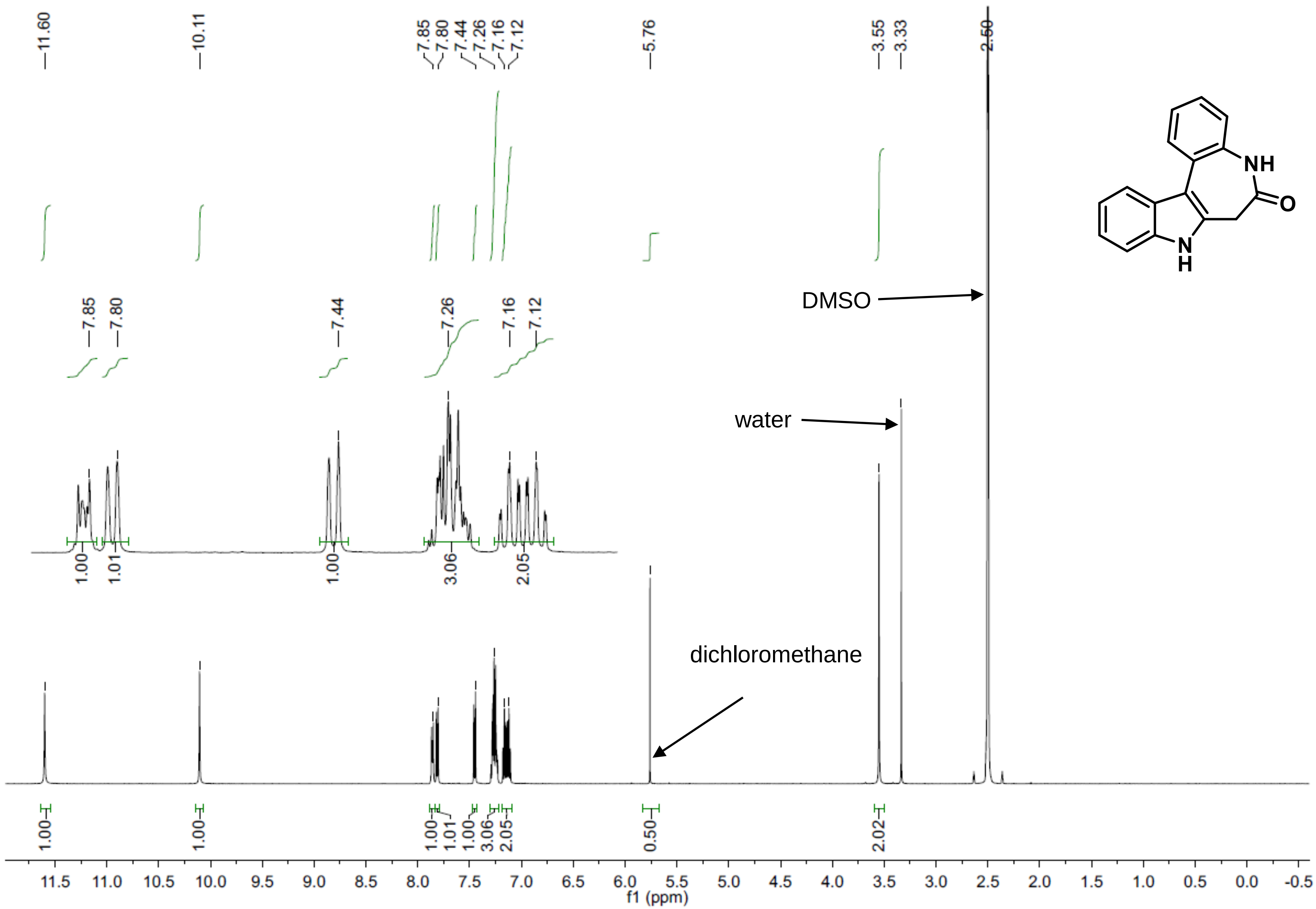

Figure S23: ^{1}H NMR spectrum of **C** in DMSO (500 MHz).

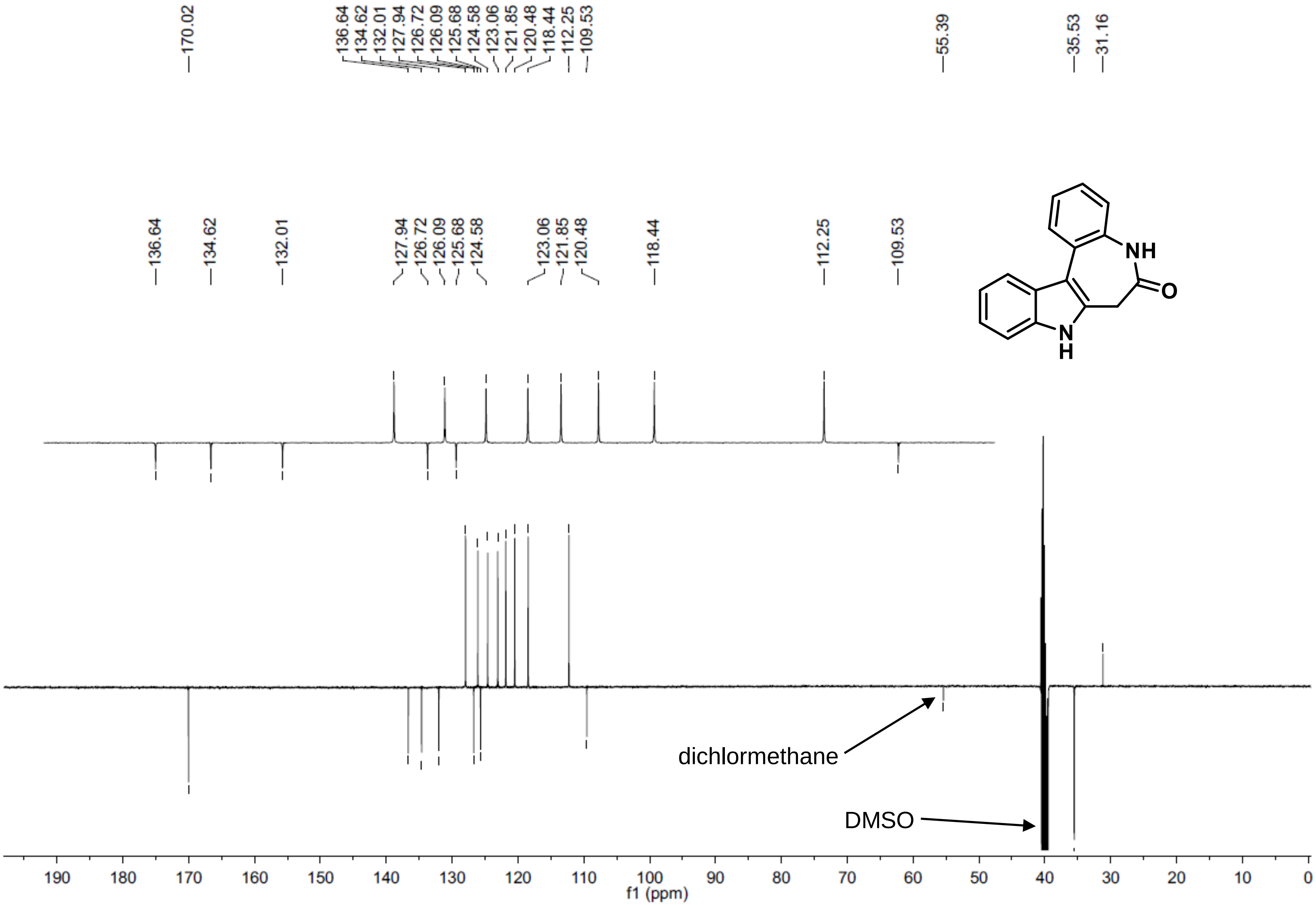

Figure S24: ^{13}C NMR spectrum of **C** in DMSO (126 MHz).

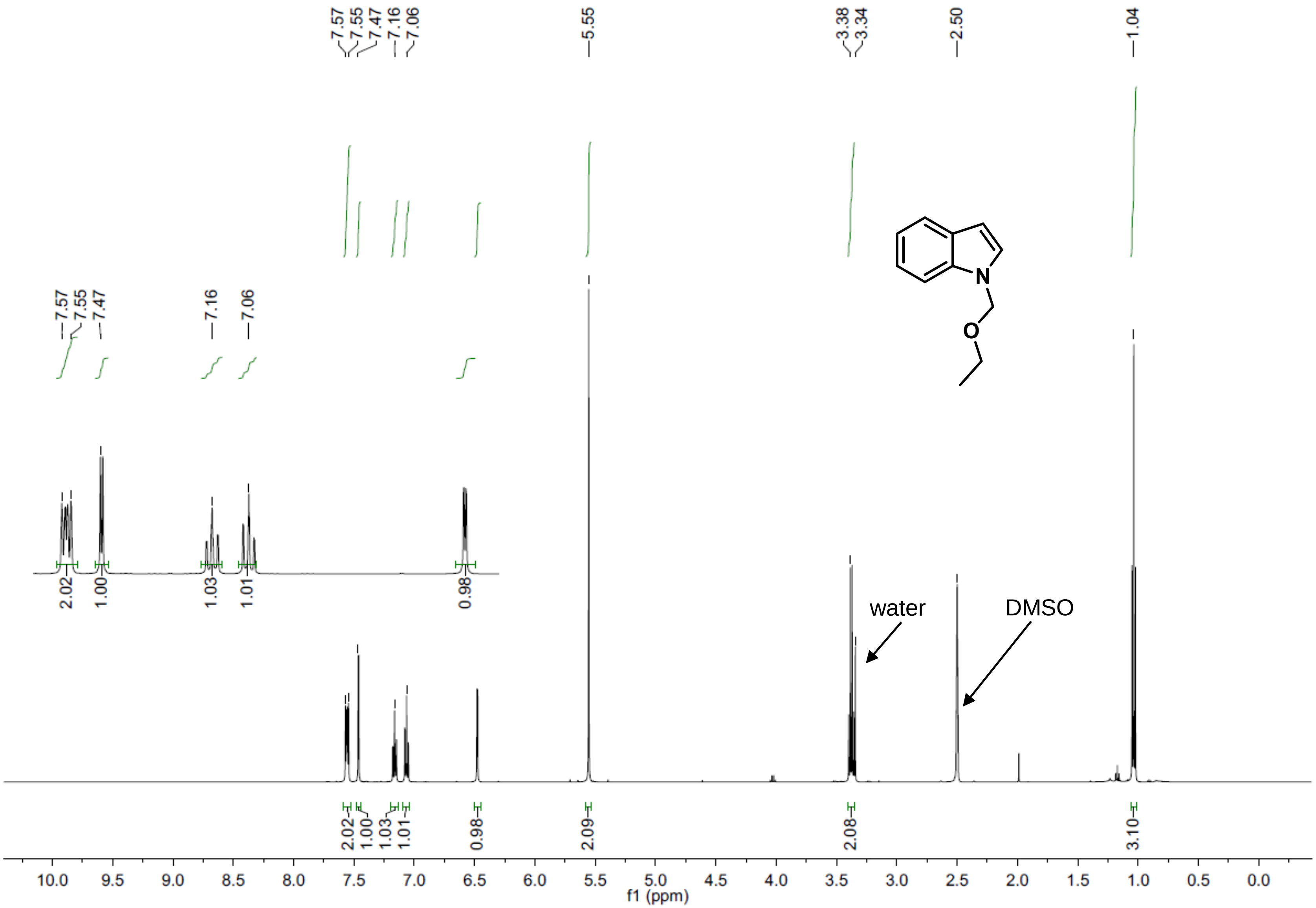

Figure S25: ^{1}H NMR spectrum of **4** in DMSO (500 MHz).

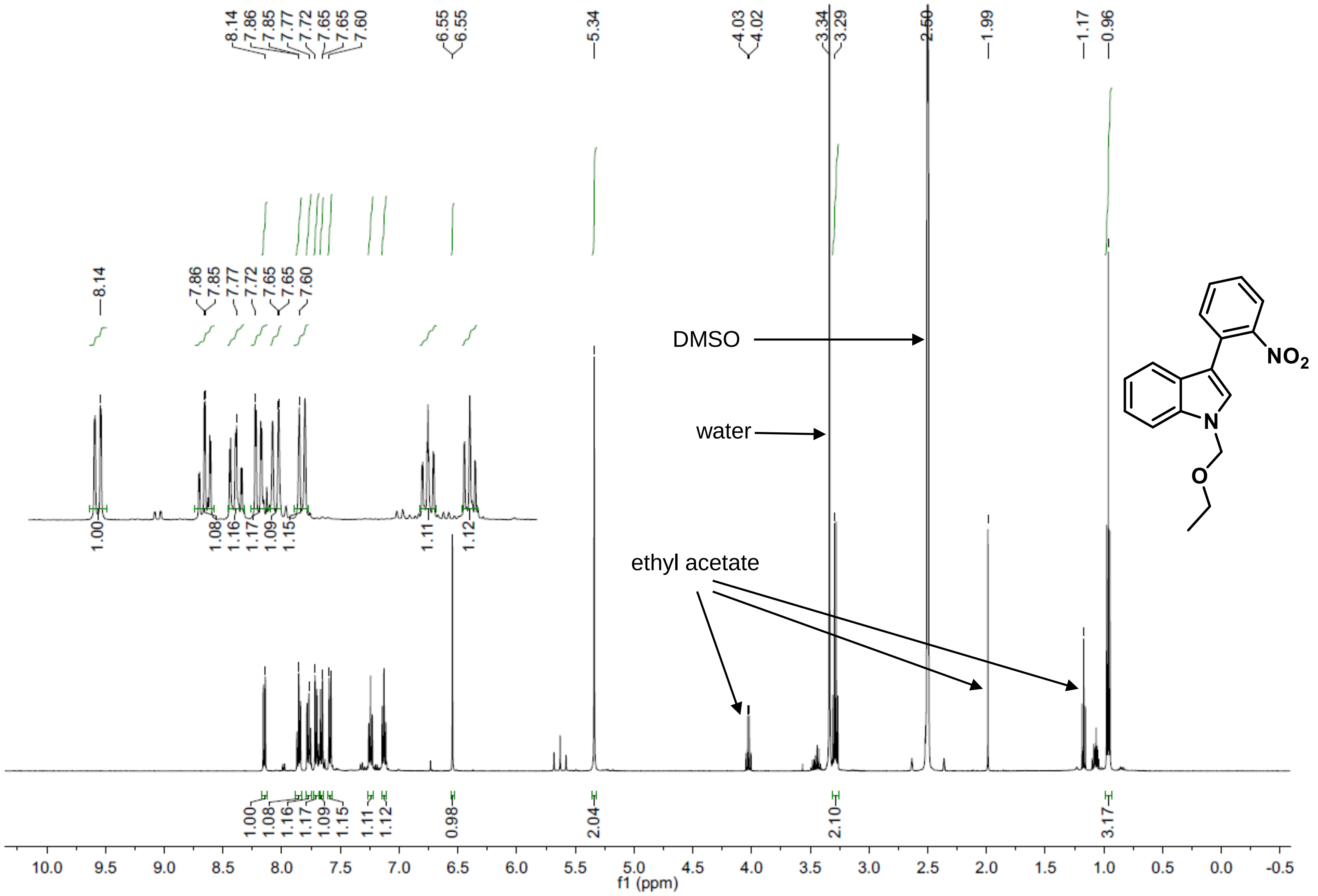

Figure S26. ^{1}H NMR spectrum of **5** in DMSO (500 MHz).

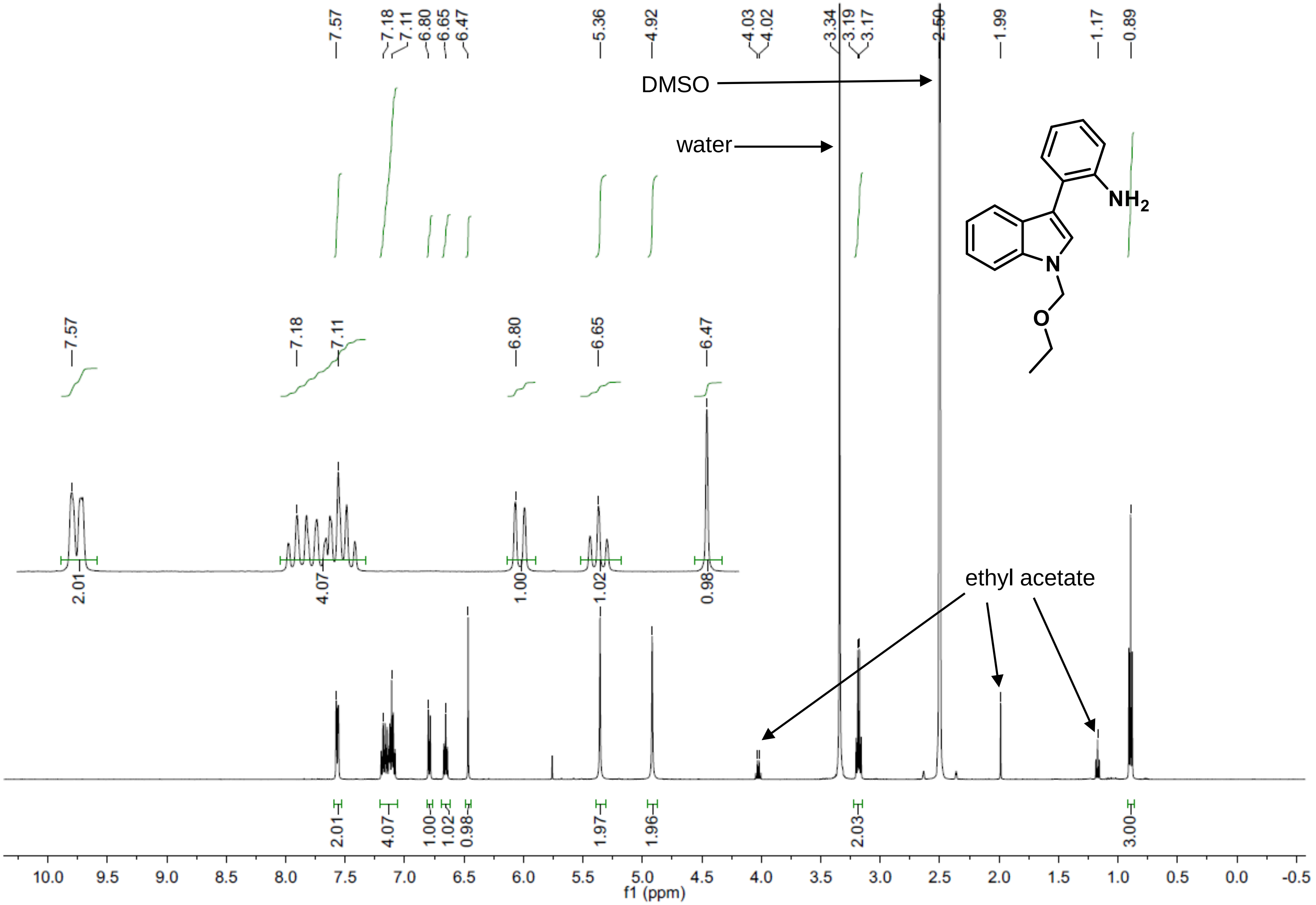

Figure S27: ^{1}H NMR spectrum of **6** in DMSO (500 MHz).

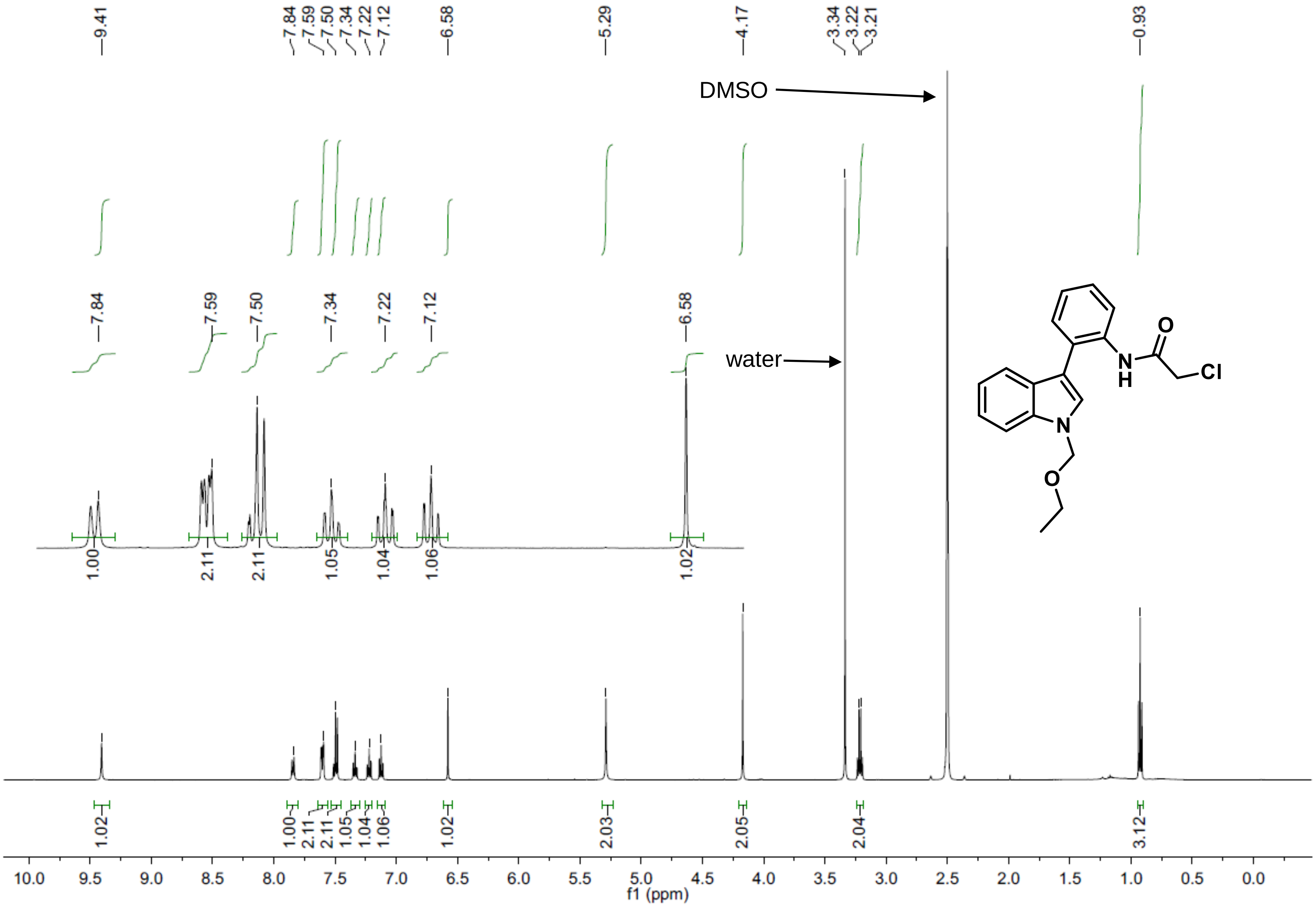

Figure S28: ^{1}H NMR spectrum of **7** in DMSO (500 MHz).

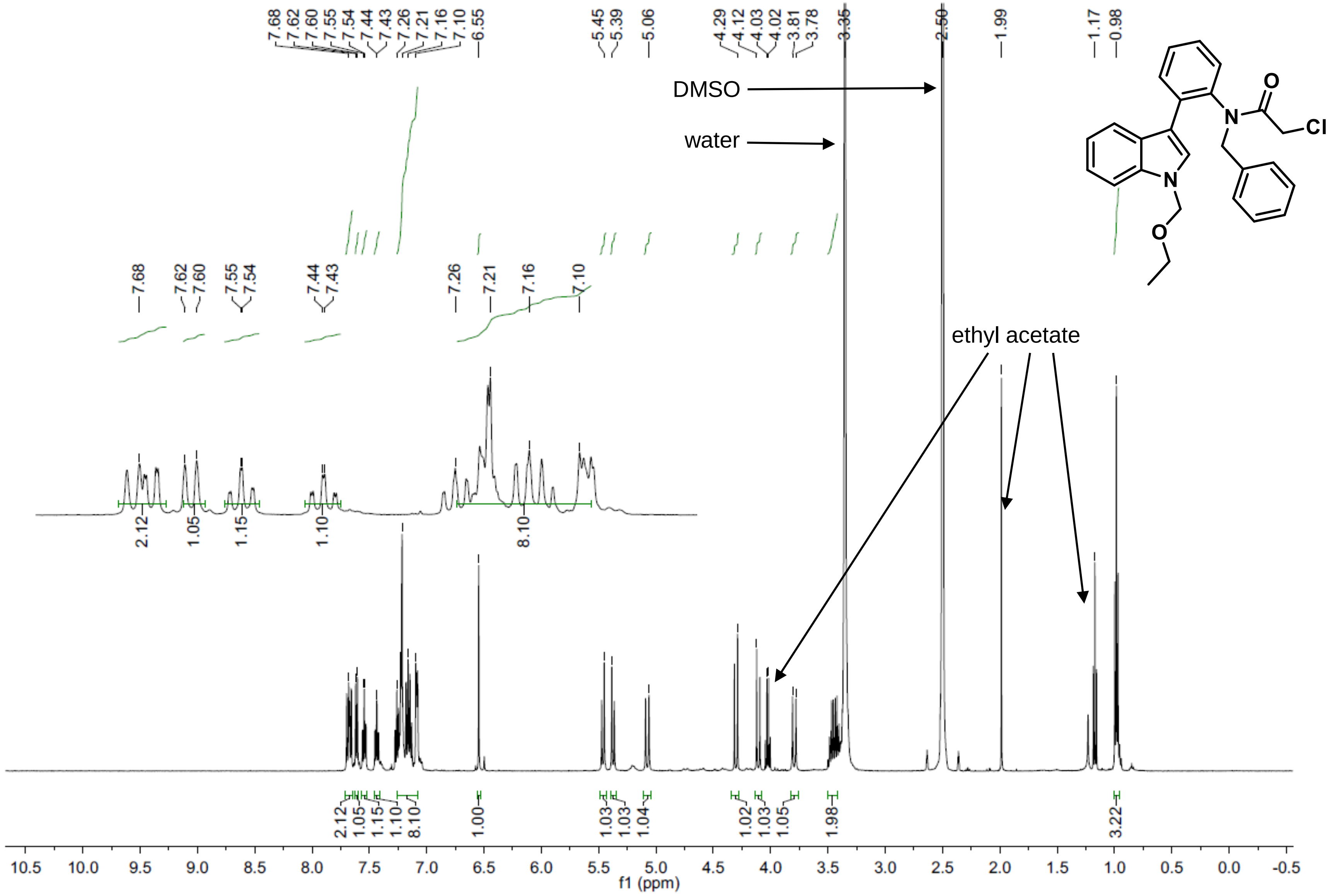

Figure S29: ^{1}H NMR spectrum of **8** in DMSO (500 MHz).

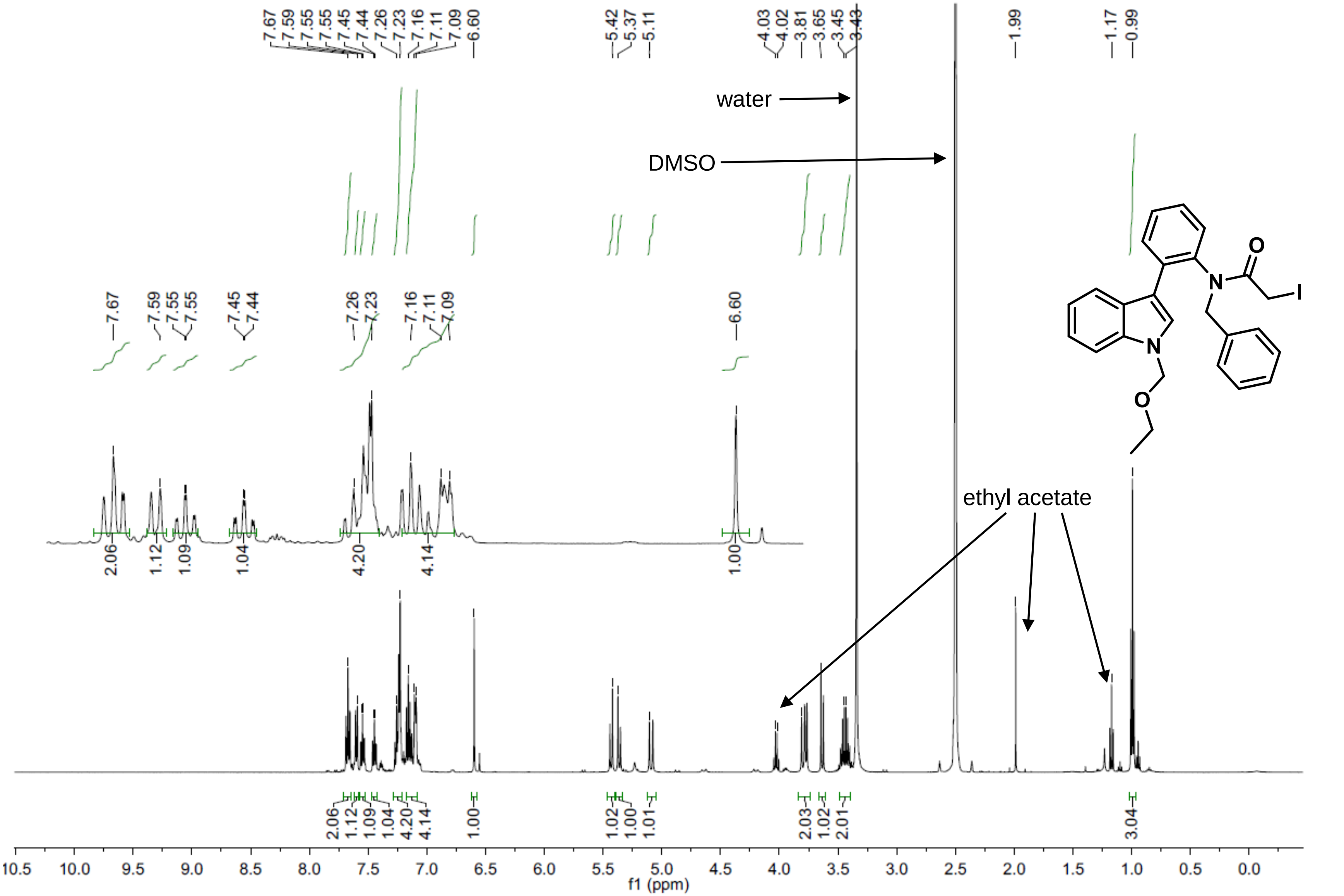

Figure S30: ^{1}H NMR spectrum of **9** in DMSO (500 MHz).

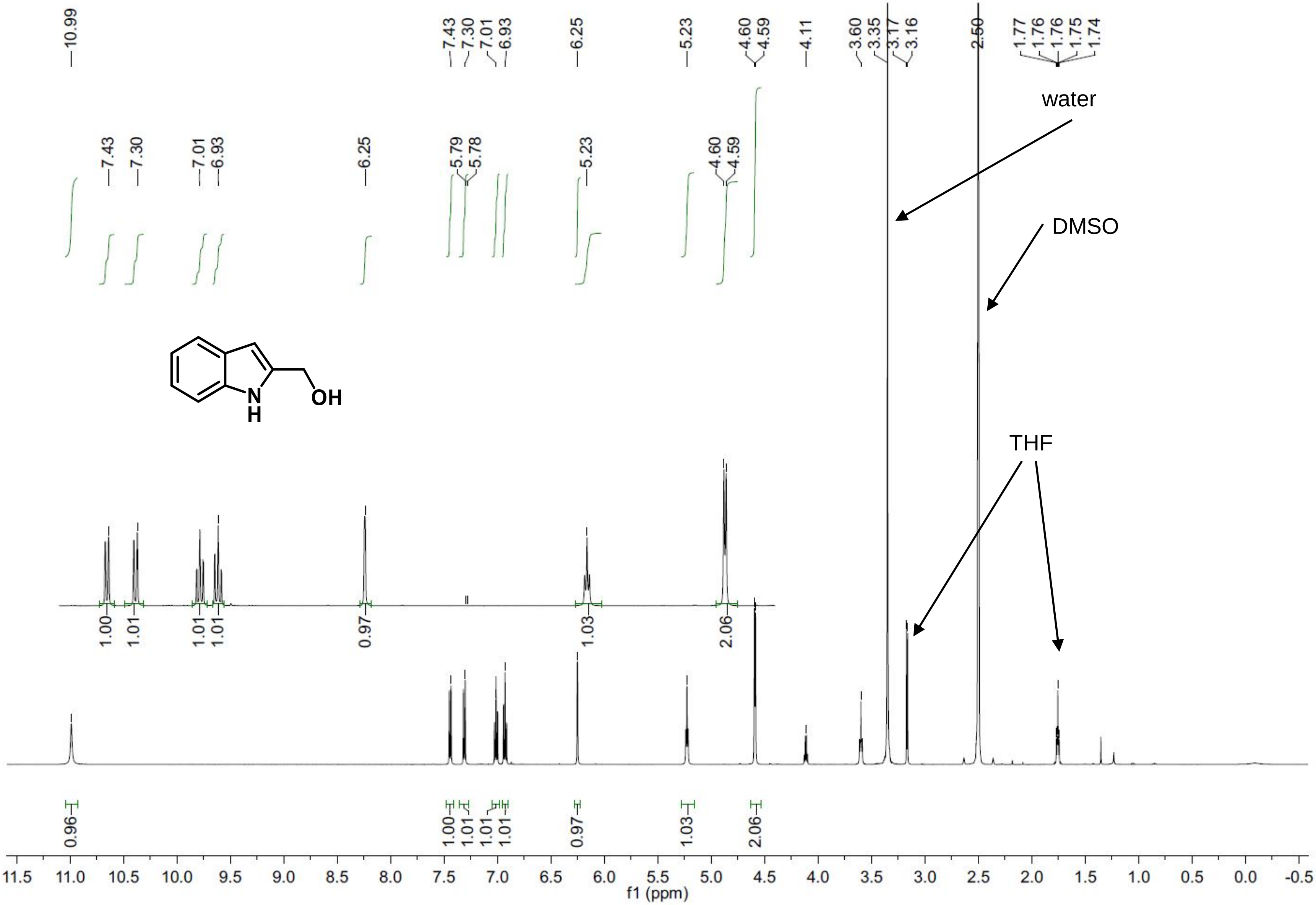

Figure S31: ^{1}H NMR spectrum of **10** in DMSO (500 MHz).

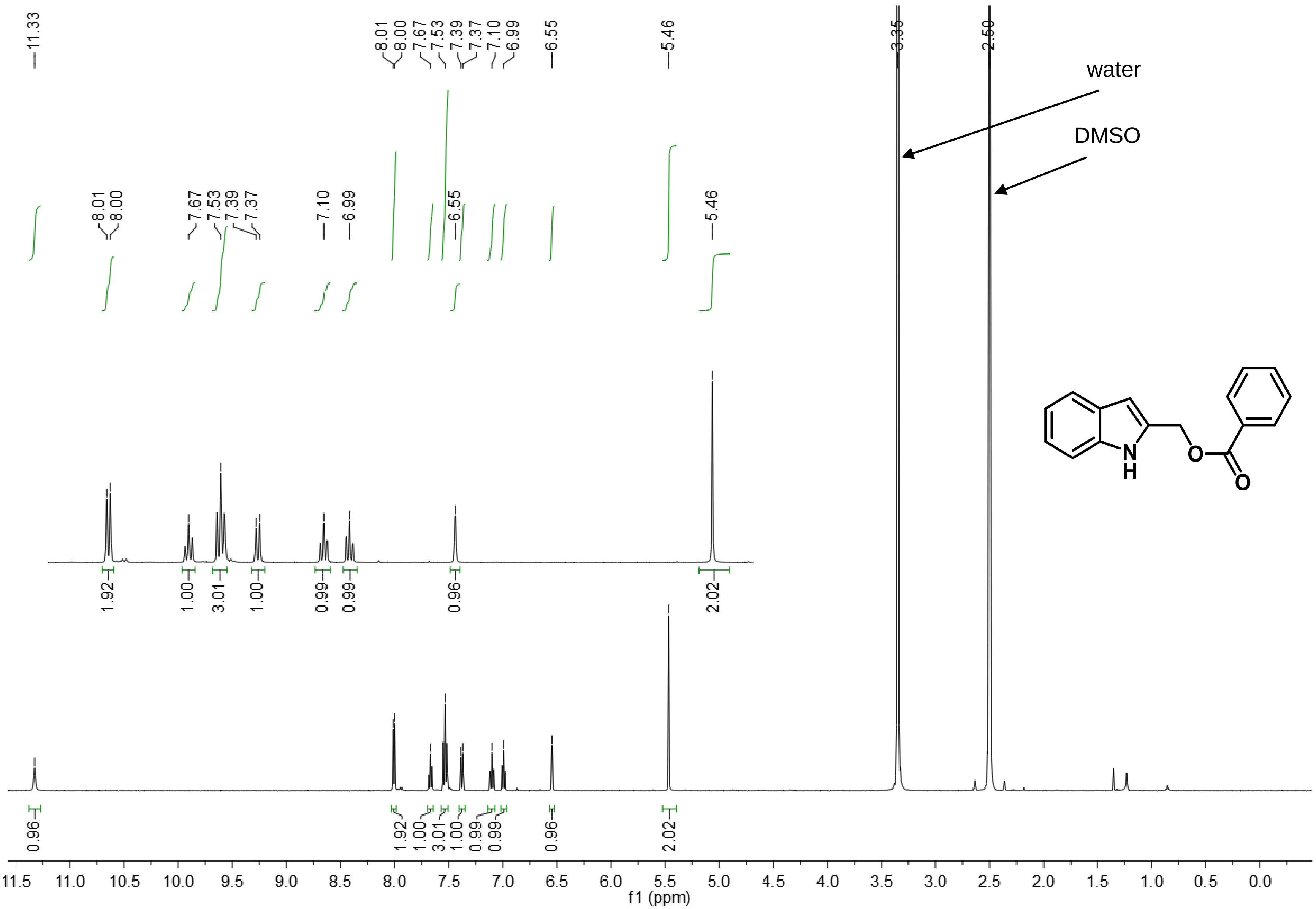

Figure S32: ^{1}H NMR spectrum of **11** in DMSO (500 MHz).

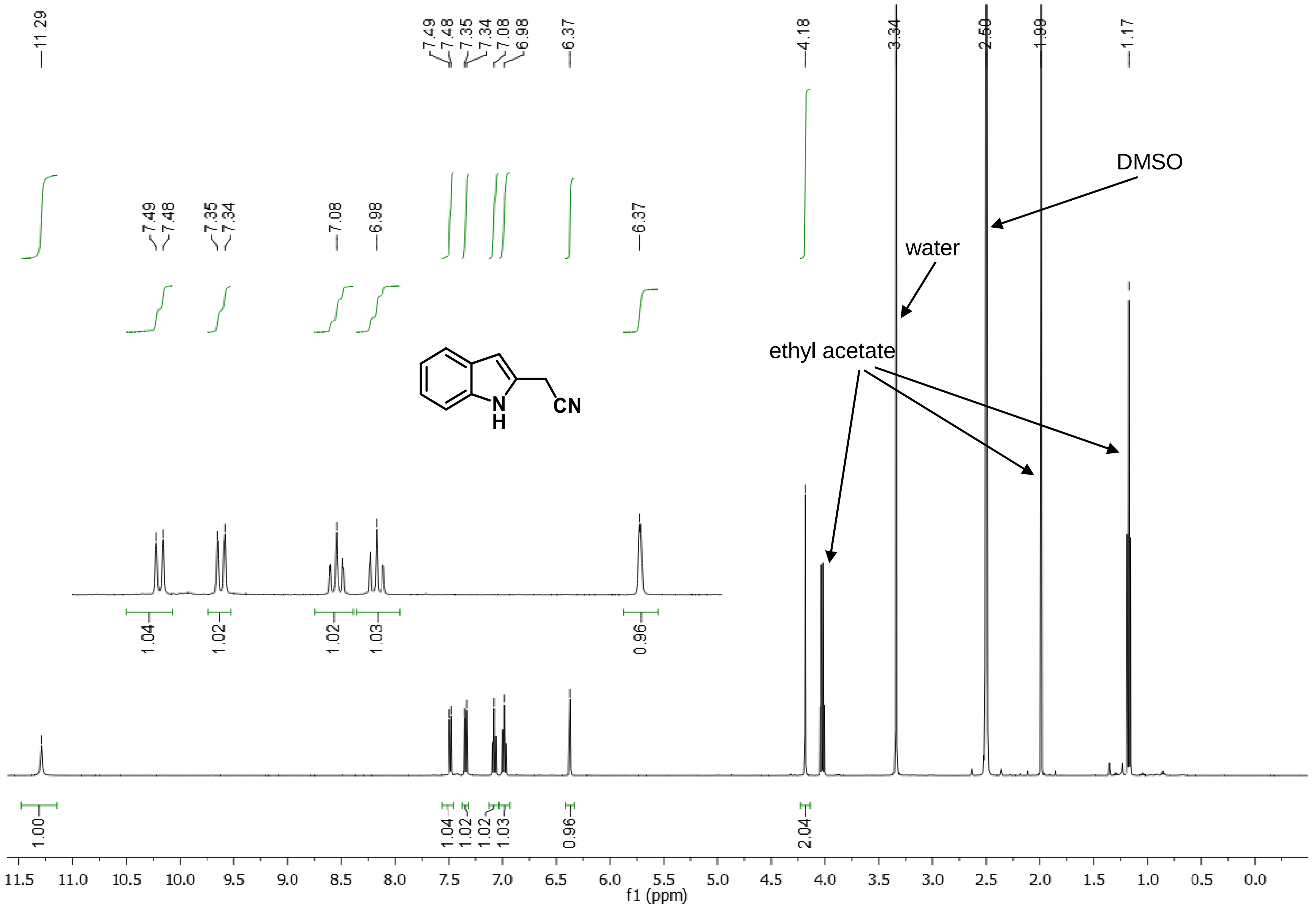

Figure S33: ^{1}H NMR spectrum of **12** in DMSO (500 MHz).

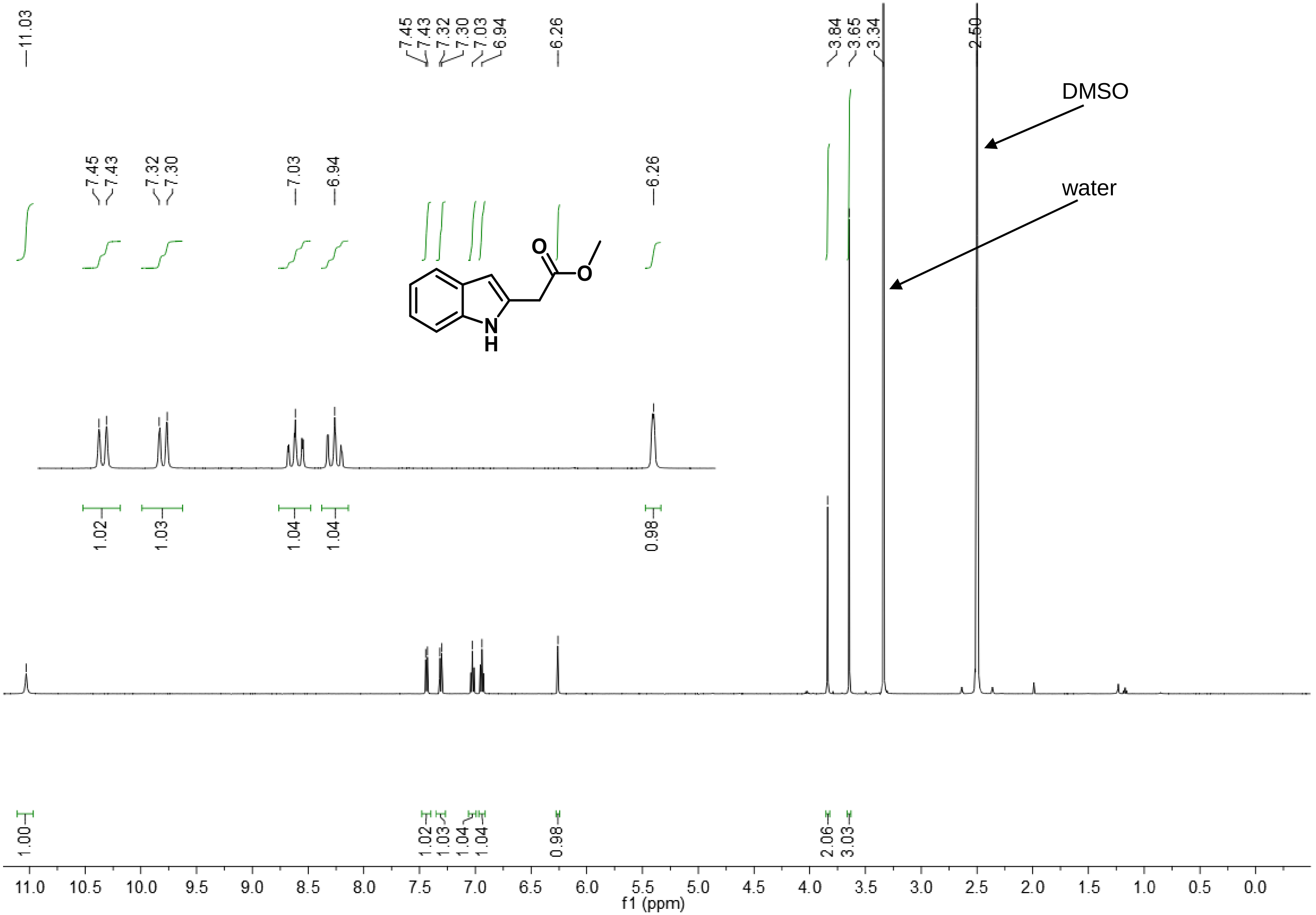

Figure S34: ^{1}H NMR spectrum of **13** in DMSO (500 MHz).

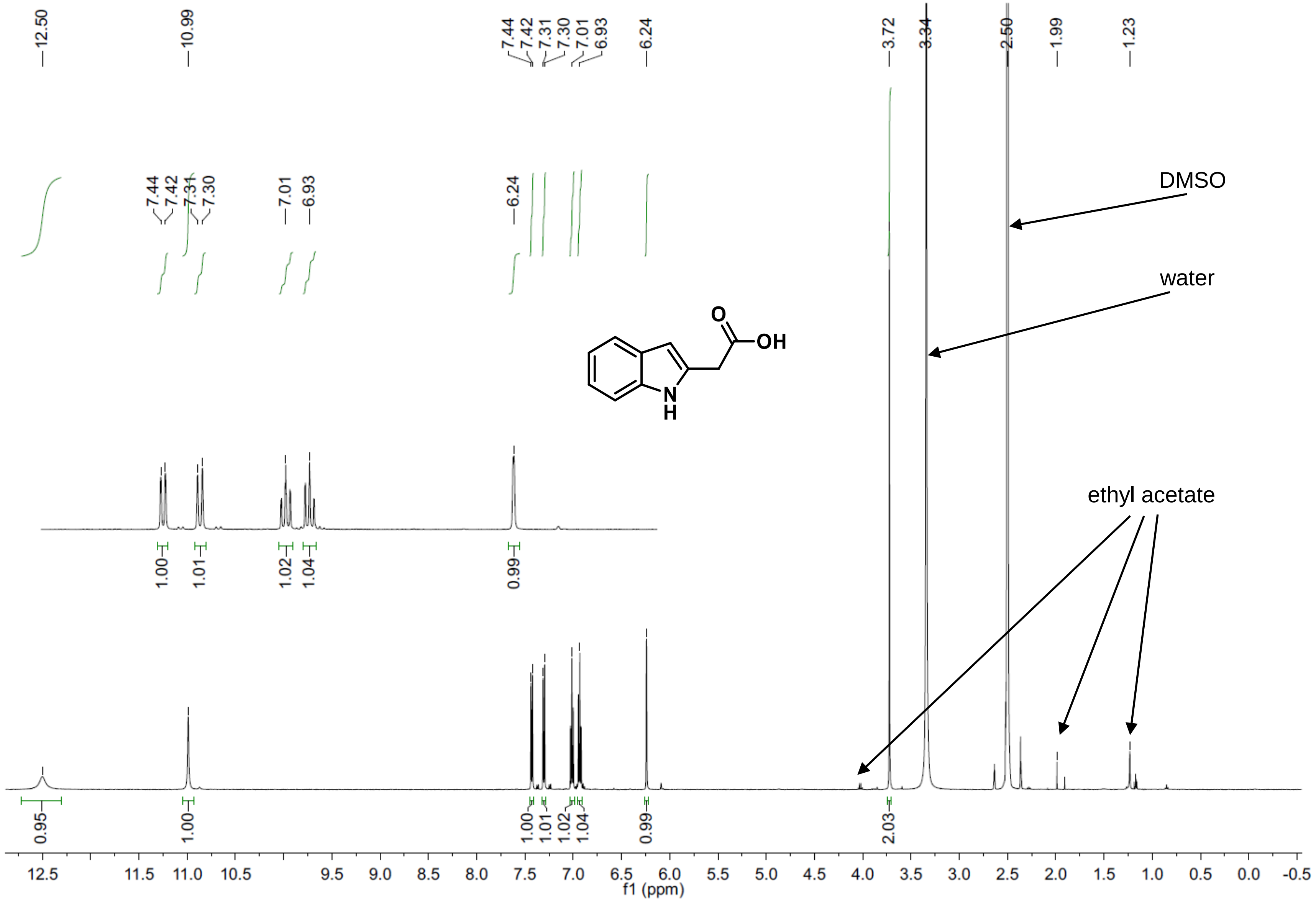

Figure S35: ^{1}H NMR spectrum of **14** in DMSO (500 MHz).

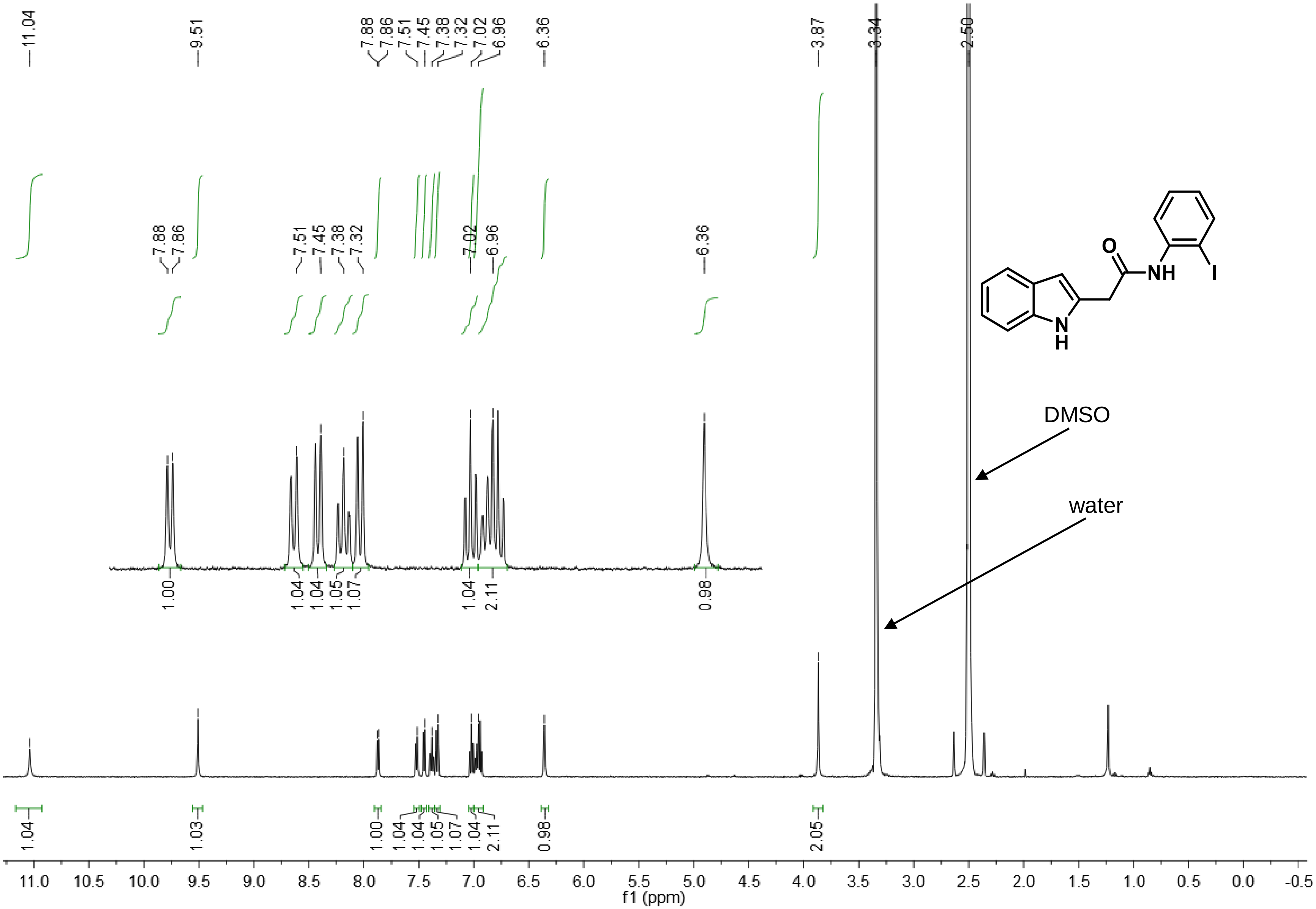

Figure S36: ^{1}H NMR spectrum of **15** in DMSO (500 MHz).

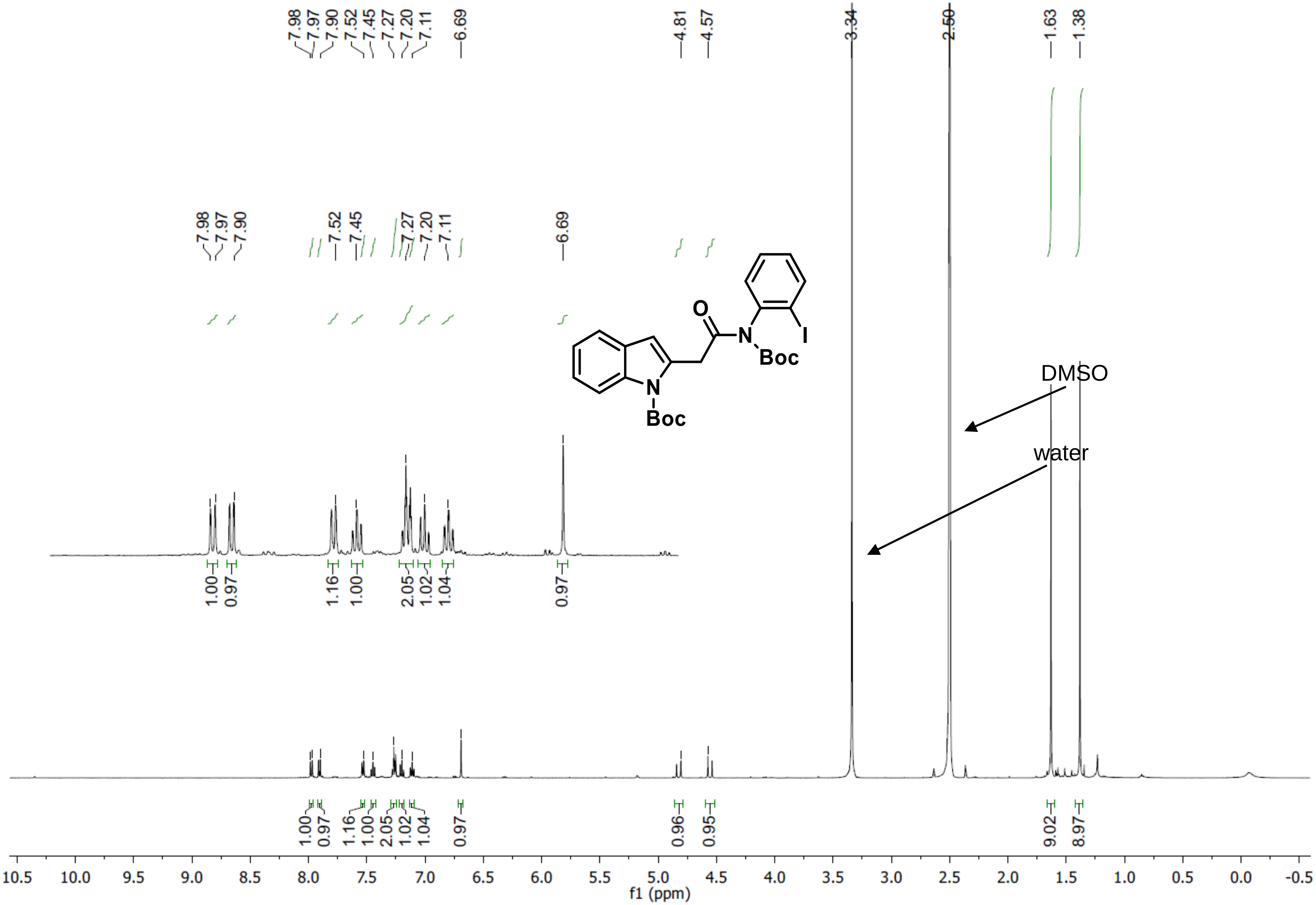

Figure S37: ^{1}H NMR spectrum of **16** in DMSO (500 MHz).

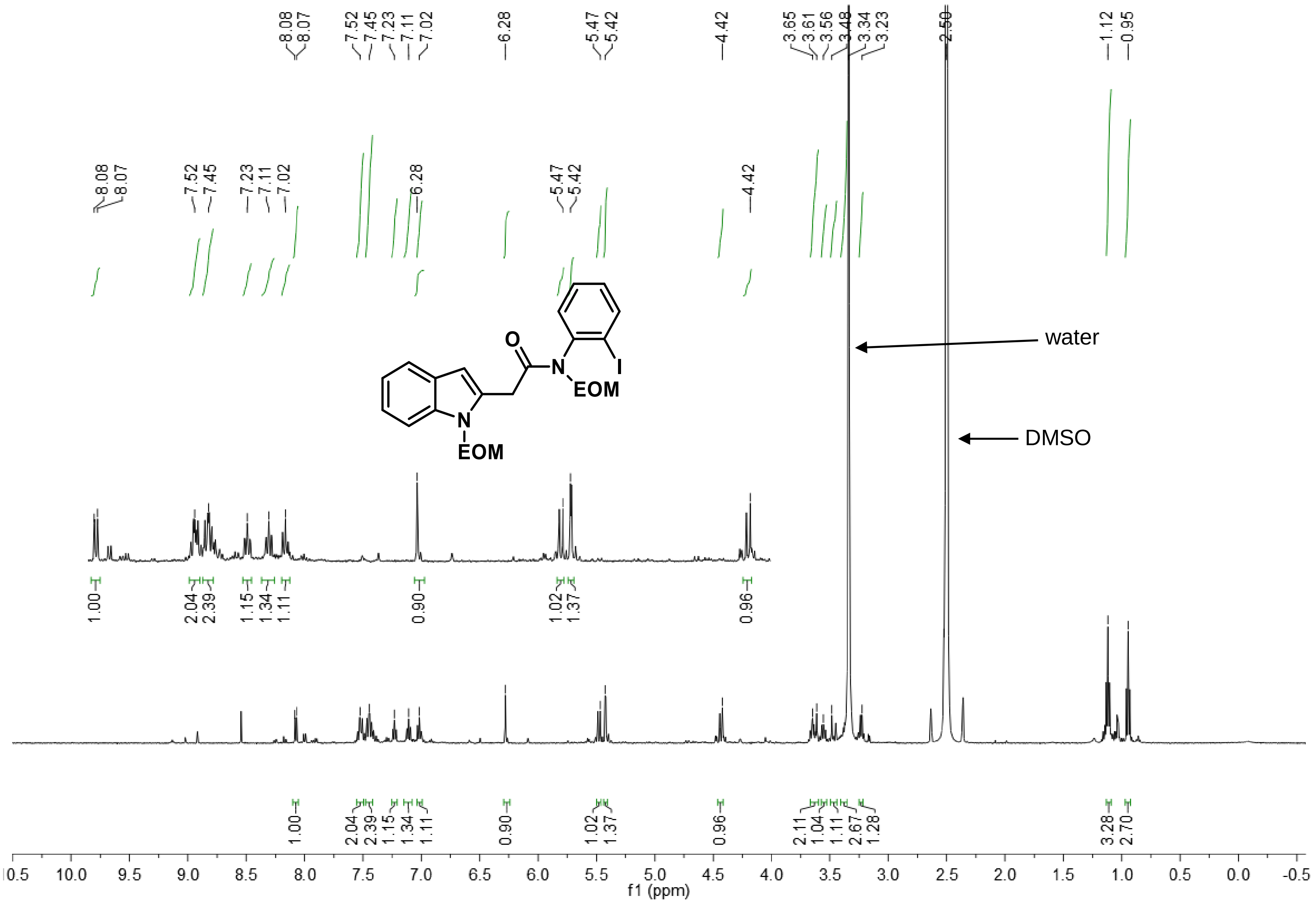

Figure S38: ^{1}H NMR spectrum of **17** in DMSO (500 MHz).

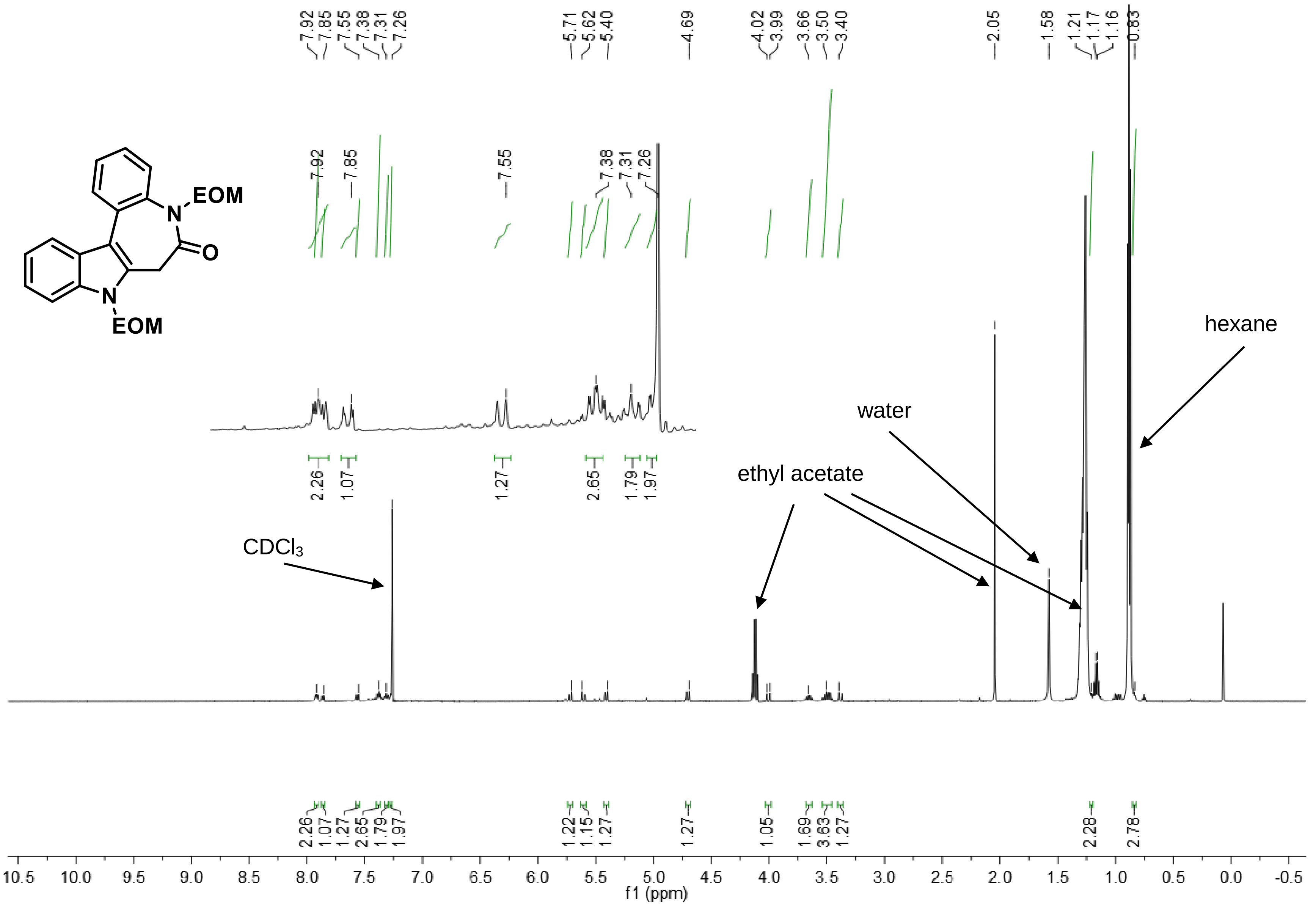

Figure S39: ^{1}H NMR spectrum of **18** in $CDCl_3$ (500 MHz).

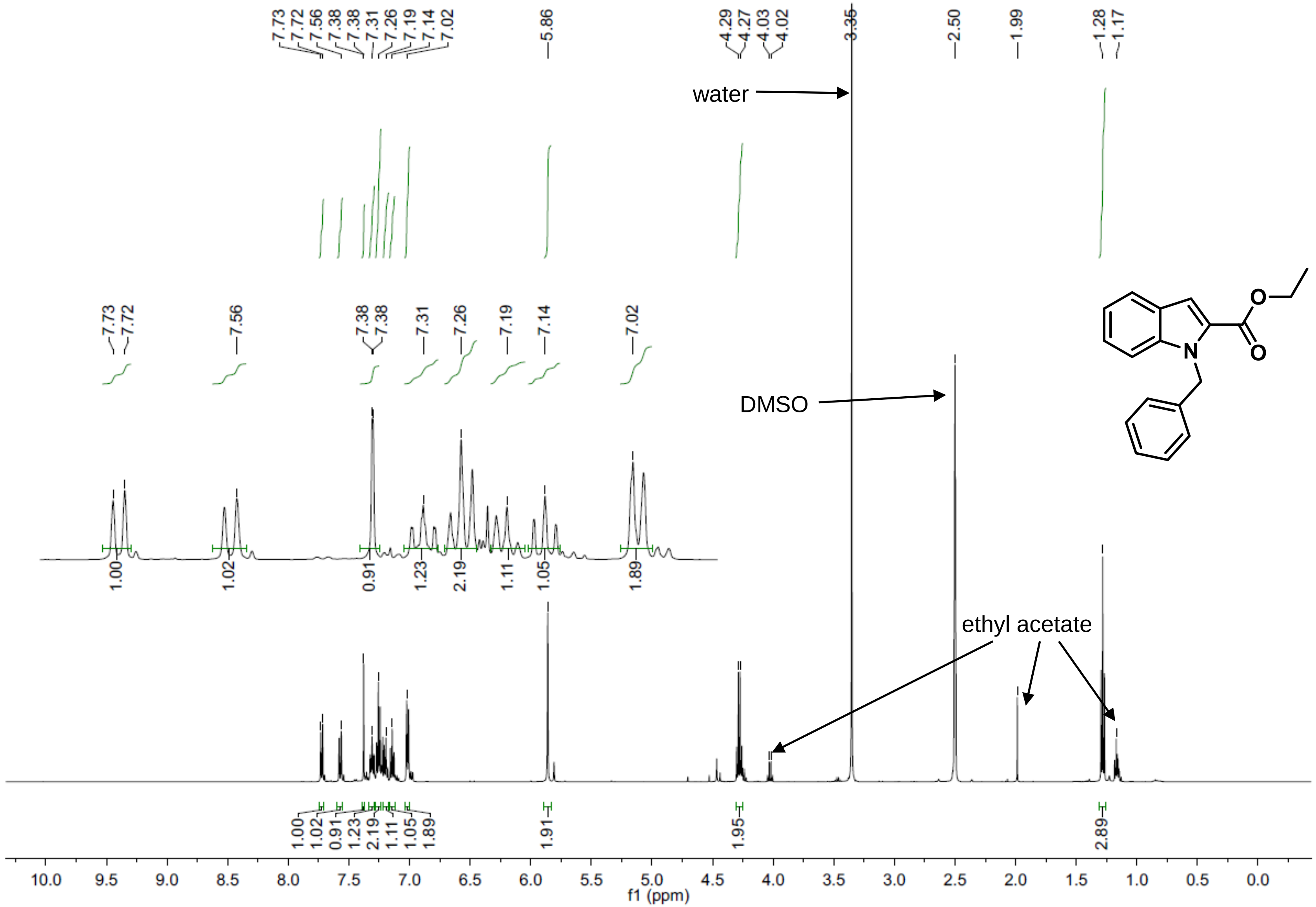

Figure S40: ^{1}H NMR spectrum of **19** in DMSO (500 MHz).

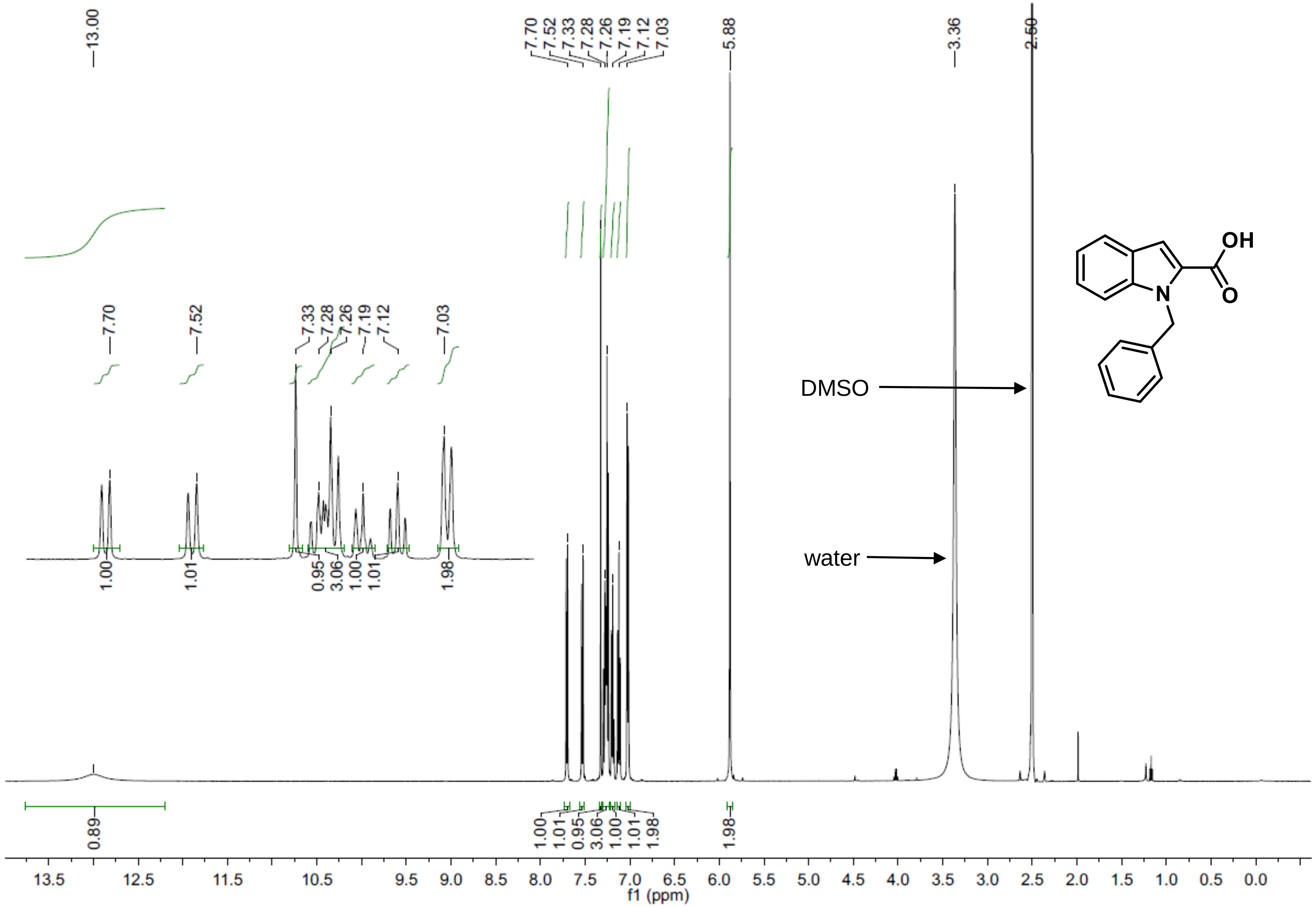

Figure S41: ^{1}H NMR spectrum of **20** in DMSO (500 MHz).

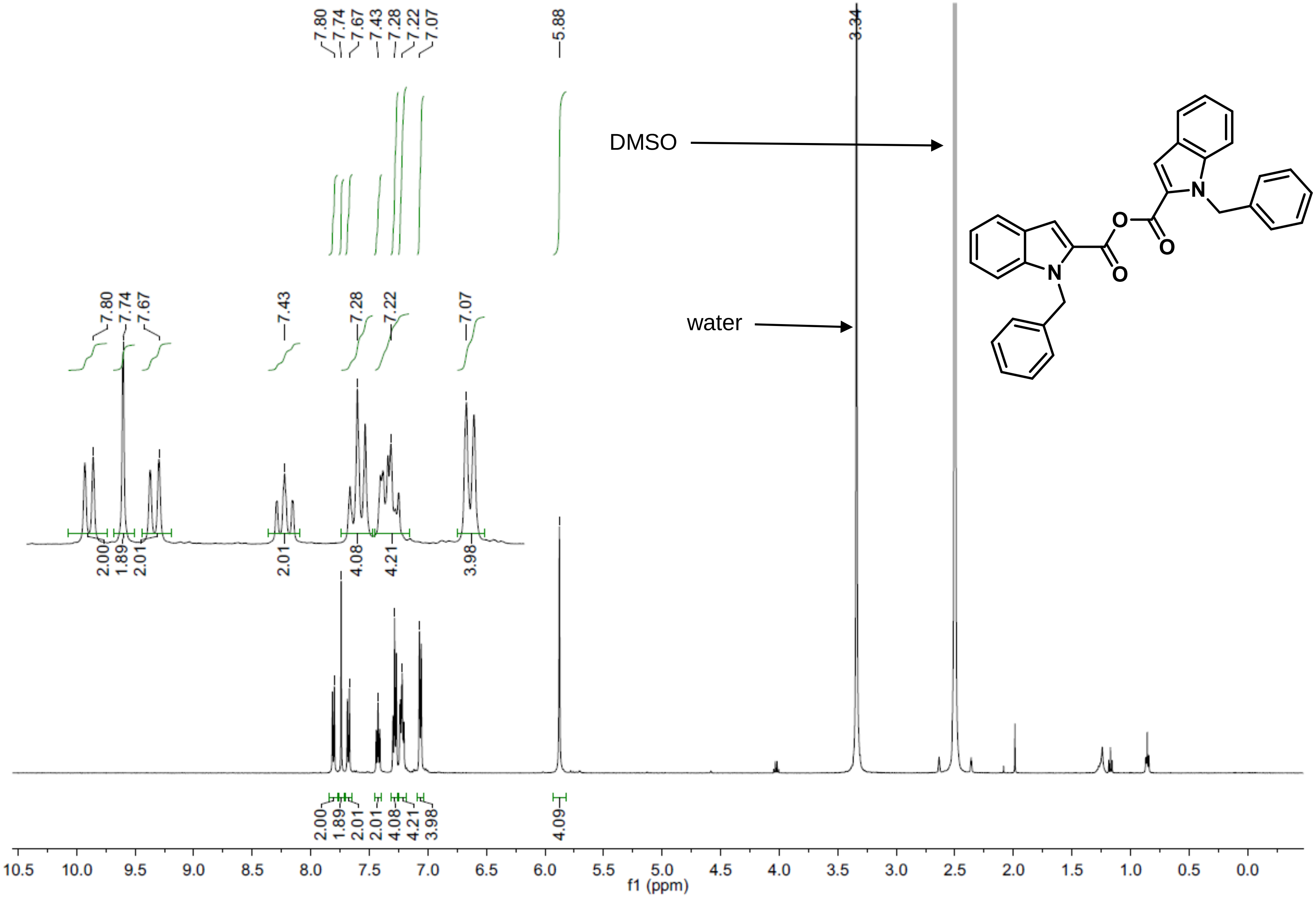

Figure S42: ^{1}H NMR spectrum of **21** in DMSO (500 MHz).

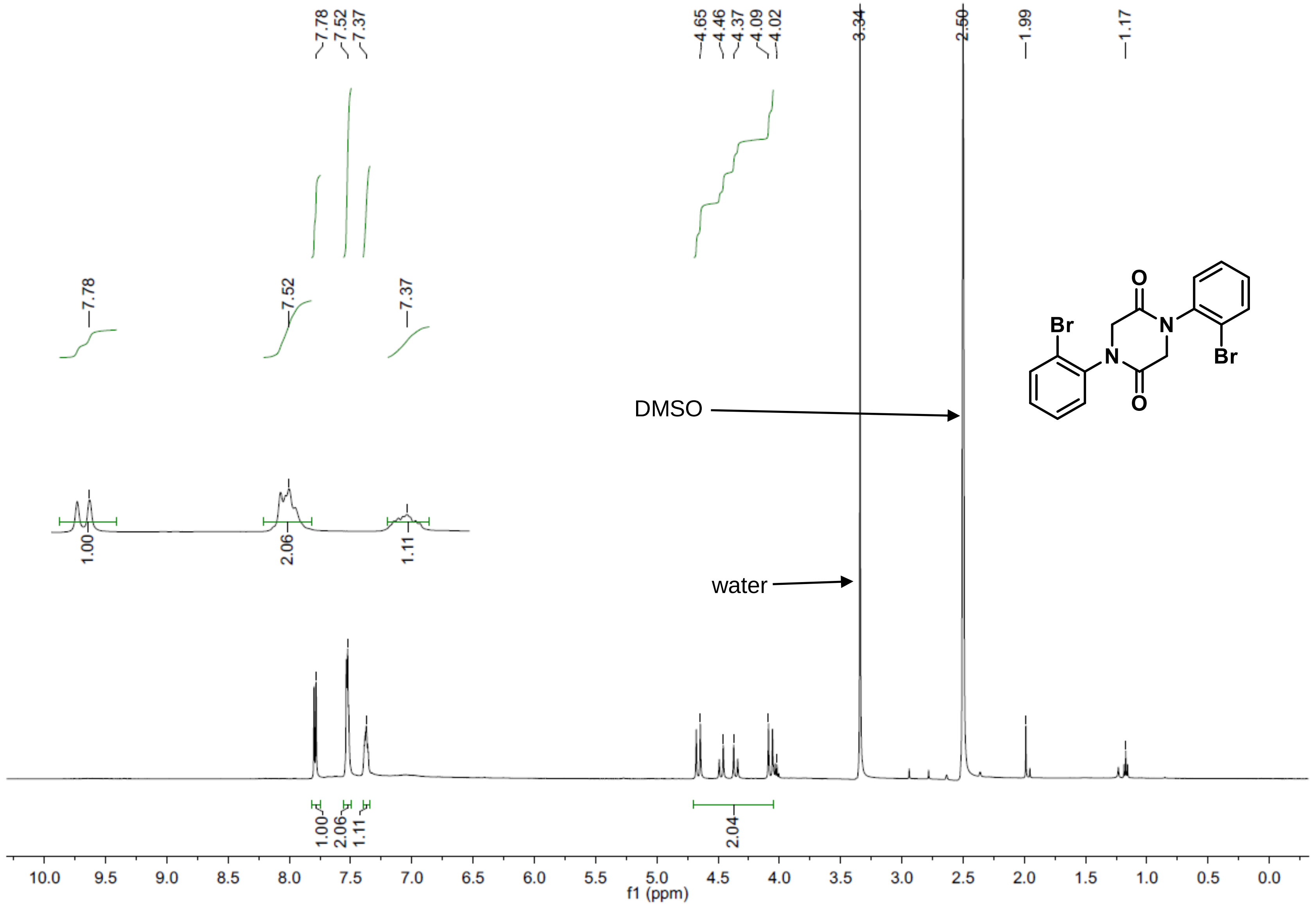

Figure S43: ^{1}H NMR spectrum of **22** in DMSO (500 MHz).

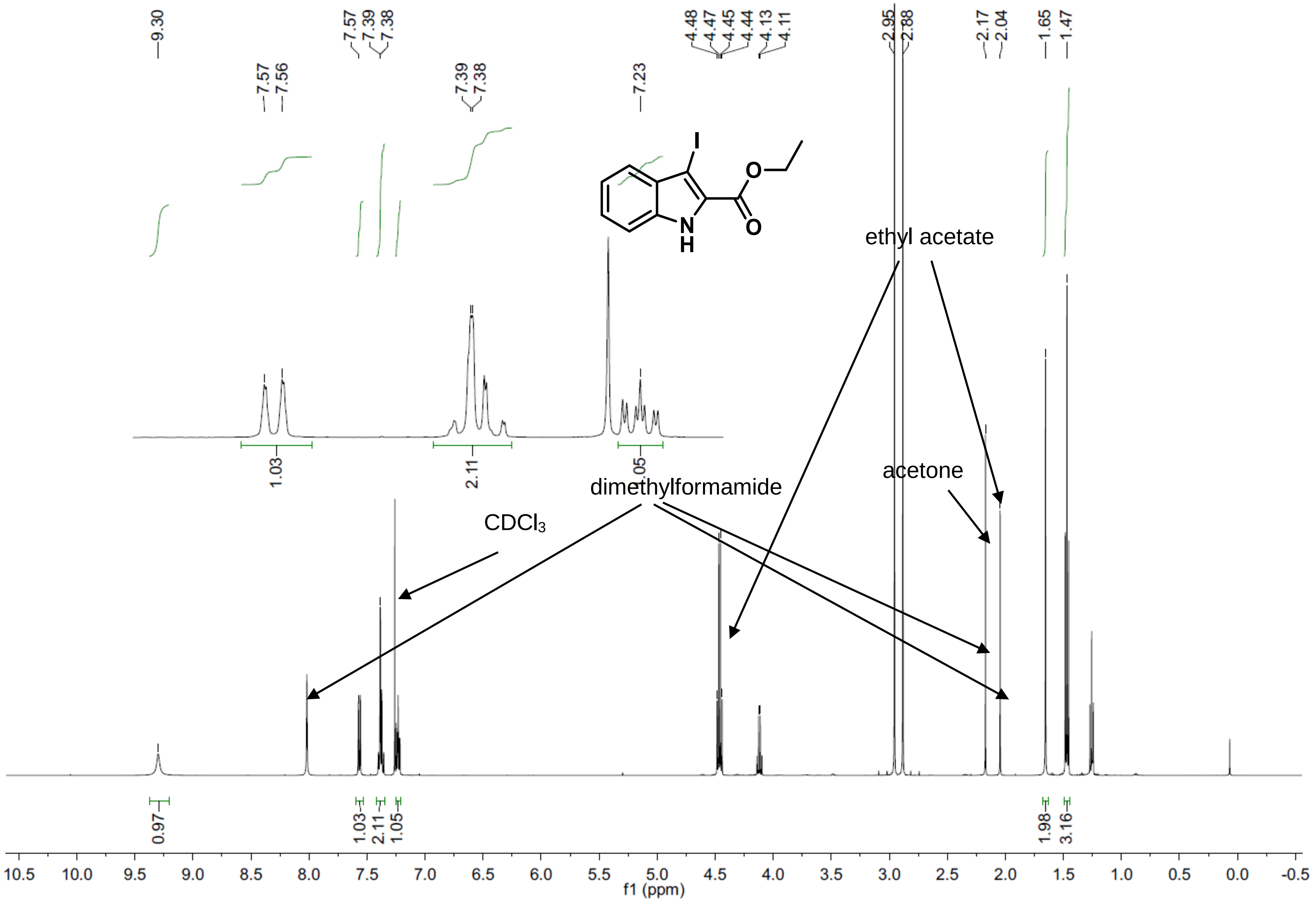

Figure S44: ^{1}H NMR spectrum of **23** in $CDCl_3$ (500 MHz).

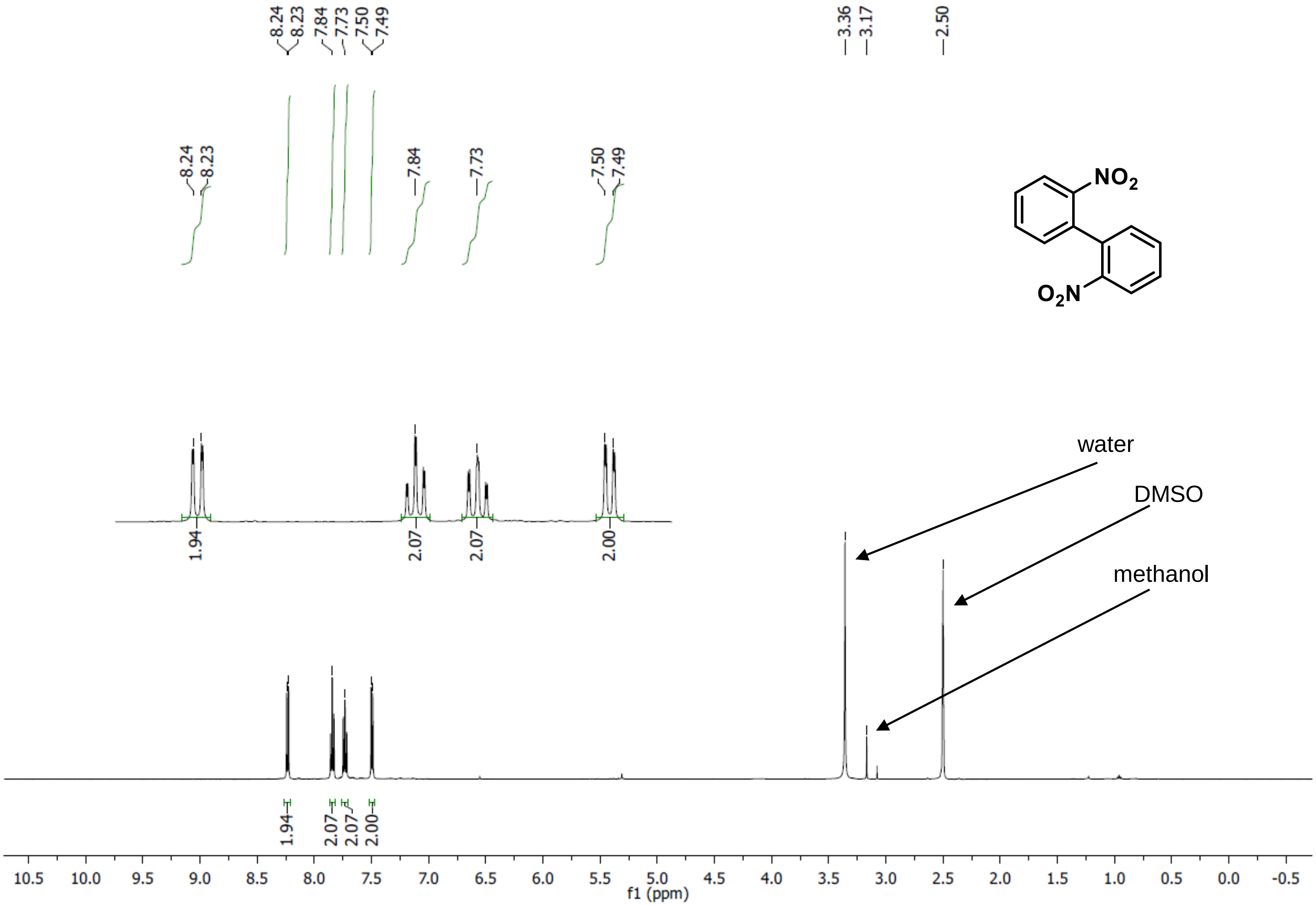

Figure S45: ^{1}H NMR spectrum of **24** in DMSO (500 MHz).

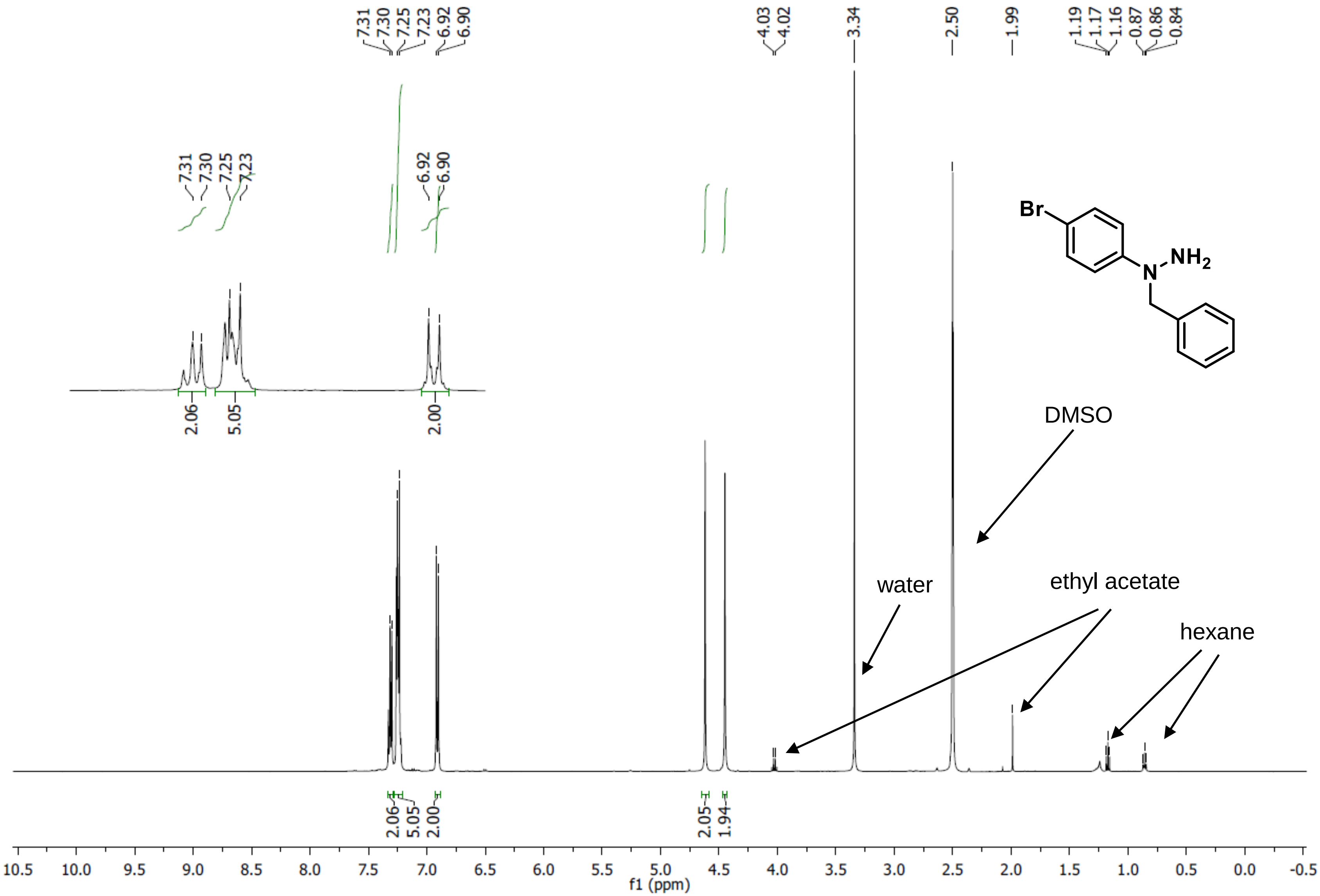

Figure S46: ^{1}H NMR spectrum of 1-benzyl-1-(4-bromophenyl)hydrazine in DMSO (500 MHz).

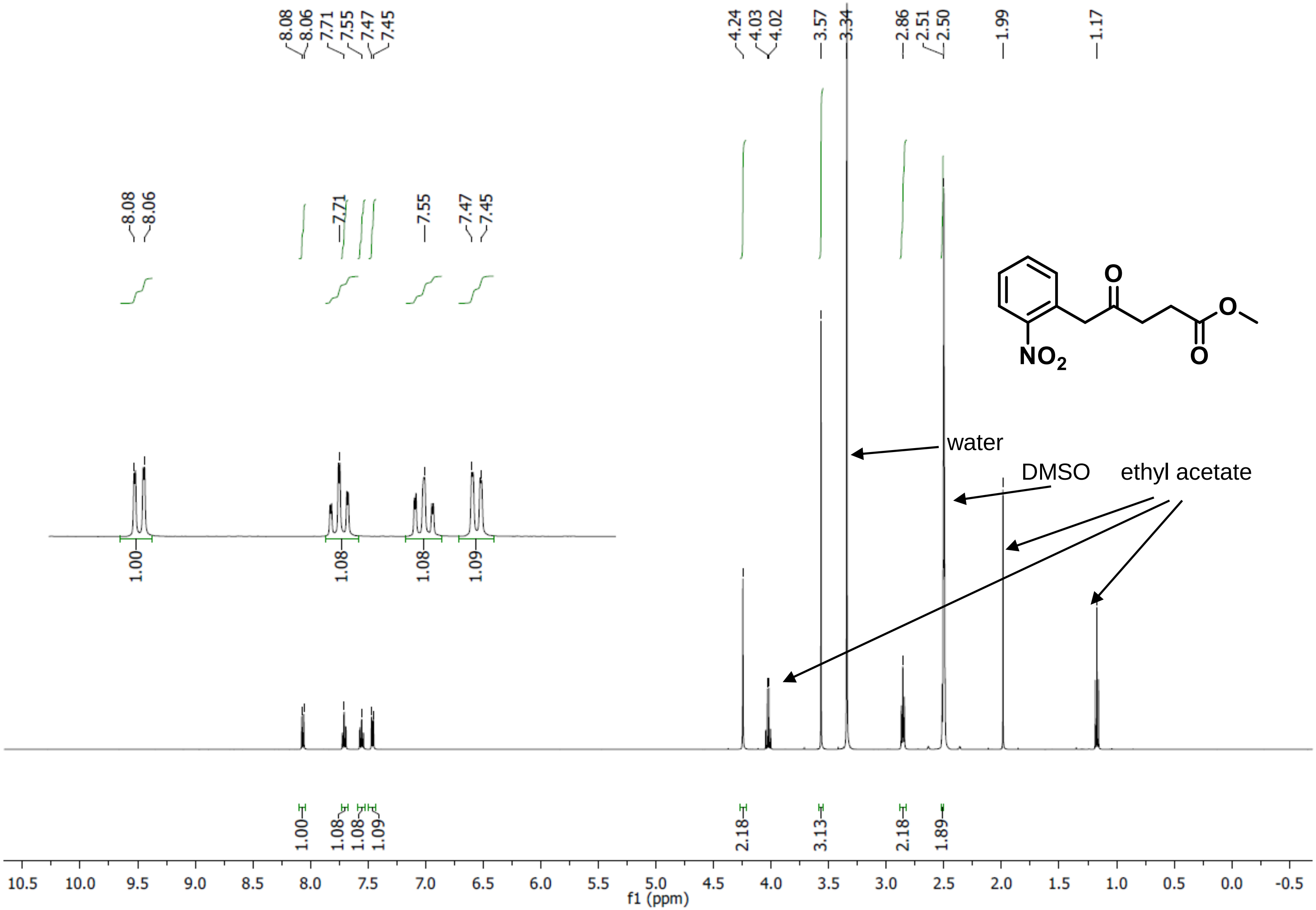

Figure S47: ^{1}H NMR spectrum of methyl 5-(2-nitrophenyl)-4-oxopentanoate in DMSO (500 MHz).

References

(1) Sheldrick, G. M. *Acta Crystallogr. A* **2008**, *64* (1), 112–122. doi:10.1107/S0108767307043930

(2) Peng, L.; Zhang, T.; Li, Y.; Li, Y. *Synth. Commun.* **2002**, *32* (5), 785–791. doi:10.1081/SCC-120002520

(3) Viti, G.; Giannotti, D.; Nannicini, R.; Ricci, R.; Pestellini, V. *J. Heterocycl. Chem.* **1990**, *27* (5), 1369–1375. doi:10.1002/jhet.5570270535

(4) Williams, D. B. G.; Lawton, M. *J. Org. Chem.* **2010**, *75* (24), 8351–8354. doi:10.1021/jo101589h

(5) Bunce, R. A.; Nammalwar, B. *J. Heterocycl. Chem.* **2009**, *46* (2), 172–177. doi:10.1002/jhet.22

(6) Chaudhary, P.; Gupta, S.; Sureshbabu, P.; Sabiah, S.; Kandasamy, J. *Green Chem.* **2016**, *18* (23), 6215–6221. doi:10.1039/C6GC02444K

(7) Dvořák, Z.; Kopp, F.; Costello, C. M.; Kemp, J. S.; Li, H.; Vrzalová, A.; Štěpánková, M.; Bartoňková, I.; Jiskrová, E.; Poulíková, K.; Vyhlídalová, B.; Nordstroem, L. U.; Karunaratne, C. V.; Ranhotra, H. S.; Mun, K. S.; Naren, A. P.; Murray, I. A.; Perdew, G. H.; Brtko, J.; Toporova, L.; Schön, A.; Wallace, B. D.; Walton, W. G.; Redinbo, M. R.; Sun, K.; Beck, A.; Kortagere, S.; Neary, M. C.; Chandran, A.; Vishveshwara, S.; Cavalluzzi, M. M.; Lentini, G.; Cui, J. Y.; Gu, H.; March, J. C.; Chatterjee, S.; Matson, A.; Wright, D.; Flannigan, K. L.; Hirota, S. A.; Sartor, R. B.; Mani, S. *EMBO Mol. Med.* **2020**, *12* (4). doi:10.15252/emmm.201911621

(8) Singla, R.; Gupta, K. B.; Upadhyay, S.; Dhiman, M.; Jaitak, V. *Eur. J. Med. Chem.* **2018**, *146*, 206–219. doi:10.1016/j.ejmech.2018.01.051

(9) Mo, F.; Li, F.; Qiu, D.; Zhang, Y.; Wang, J. *Chin. J. Chem.* **2012**, *30* (10), 2297–2302. doi:10.1002/cjoc.201200672

(10) Nowacki, M.; Wojciechowski, K. *Beilstein J. Org. Chem.* **2018**, *14*, 194–202. doi:10.3762/bjoc.14.14

(11) Mendoza-Figueroa, H. L.; Serrano-Alva, M. T.; Aparicio-Ozores, G.; Martínez-Gudiño, G.; Suárez-Castillo, O. R.; Pérez-Rojas, N. A.; Morales-Ríos, M. S. *Med. Chem. Res.* **2018**, *27* (6), 1624–1633. doi:10.1007/s00044-018-2177-x

(12) *Org. Synth.* **1922**, *2*, 27. doi:10.15227/orgsyn.002.0027

(13) Weber, D.; Berger, C.; Eickelmann, P.; Antel, J.; Kessler, H. *J. Med. Chem.* **2003**, *46* (10), 1918–1930. doi:10.1021/jm0210921

(14) Sharpe, R. J.; Johnson, J. S. *J. Org. Chem.* **2015**, *80* (19), 9740–9766. doi:10.1021/acs.joc.5b01844

(15) Lepri, S.; Ceccarelli, M.; Milani, N.; Tortorella, S.; Cucco, A.; Valeri, A.; Goracci, L.; Brink, A.; Cruciani, G. *Proc. Natl. Acad. Sci.* **2017**, *114* (16), E3178–E3187. doi:10.1073/pnas.1618881114

(16) Yao, C.-H.; Hsieh, T.-C.; Song, J.-S.; Lee, J.-C. *Future Med. Chem.* **2020**, *12* (3), 183–192. doi:10.4155/fmc-2019-0276

(17) Rajasekar, S.; Anbarasan, P. *Org. Lett.* **2019**, *21* (9), 3067–3071. doi:10.1021/acs.orglett.9b00652

(18) Singla, R.; Prakash, K.; Bihari Gupta, K.; Upadhyay, S.; Dhiman, M.; Jaitak, V. *Bioorganic Chem.* **2018**, *79*, 72–88. doi:10.1016/j.bioorg.2018.04.002

(19) Zhang, K.; Xu, X.; Zheng, J.; Yao, H.; Huang, Y.; Lin, A. *Org. Lett.* **2017**, *19* (10), 2596–2599. doi:10.1021/acs.orglett.7b00914

(20) Singla, R.; Gupta, K. B.; Upadhyay, S.; Dhiman, M.; Jaitak, V. *Bioorg. Med. Chem.* **2018**, *26* (1), 266–277. doi:10.1016/j.bmc.2017.11.040
(21) Parker, A. N.; Martin, M. C.; Shenje, R.; France, S. *Org. Lett.* **2019**, *21* (18), 7268–7273. doi:10.1021/acs.orglett.9b02498
(22) Sasho, Setsuya; Koshimura, Hirokazu; Kamisaka, Noriaki; Miki, Ichiro; Kuno, Yukako; Kumazawa, Toshiaki; Sekine, Shin. Preparation and formulation of thiazole derivatives as cell adhesion inhibitors. JP 09087282 A 19970331, 1997
(23) Jiao, L.; Bach, T. *J. Am. Chem. Soc.* **2011**, *133* (33), 12990–12993. doi:10.1021/ja2055066
(24) Jana, G. K.; Sinha, S. *Tetrahedron* **2012**, *68* (35), 7155–7165. doi:10.1016/j.tet.2012.06.027
(25) Santra, S.; Hota, P. K.; Bhattacharyya, R.; Bera, P.; Ghosh, P.; Mandal, S. K. *ACS Catal.* **2013**, *3* (12), 2776–2789. doi:10.1021/cs400468h
(26) Banwell, M. G.; Lupton, D. W.; Ma, X.; Renner, J.; Sydnes, M. O. *Org. Lett.* **2004**, *6* (16), 2741–2744. doi:10.1021/ol0490375
(27) Yasamut, K.; Jongcharoenkamol, J.; Ruchirawat, S.; Ploypradith, P. *Tetrahedron* **2016**, *72* (40), 5994–6000. doi:10.1016/j.tet.2016.07.063
(28) Hulme, R. *Acta Crystallogr.* **1962**, *15* (6), 619–619. doi:10.1107/S0365110X62001620
